# Microbiome and Human Health: Current Understanding, Engineering, and Enabling Technologies

**DOI:** 10.1021/acs.chemrev.2c00431

**Published:** 2022-11-01

**Authors:** Nikhil Aggarwal, Shohei Kitano, Ginette Ru Ying Puah, Sandra Kittelmann, In Young Hwang, Matthew Wook Chang

**Affiliations:** † NUS Synthetic Biology for Clinical and Technological Innovation (SynCTI), National University of Singapore, Singapore 117456, Singapore; ‡ Synthetic Biology Translational Research Programme, Yong Loo Lin School of Medicine, National University of Singapore, Singapore 117456, Singapore; § Wilmar-NUS (WIL@NUS) Corporate Laboratory, National University of Singapore, Singapore 117599, Singapore; ∥ Wilmar International Limited, Singapore 138568, Singapore; ⊥ Department of Biochemistry, Yong Loo Lin School of Medicine, National University of Singapore, Singapore 117596, Singapore; # Singapore Institute of Technology, Singapore 138683, Singapore

## Abstract

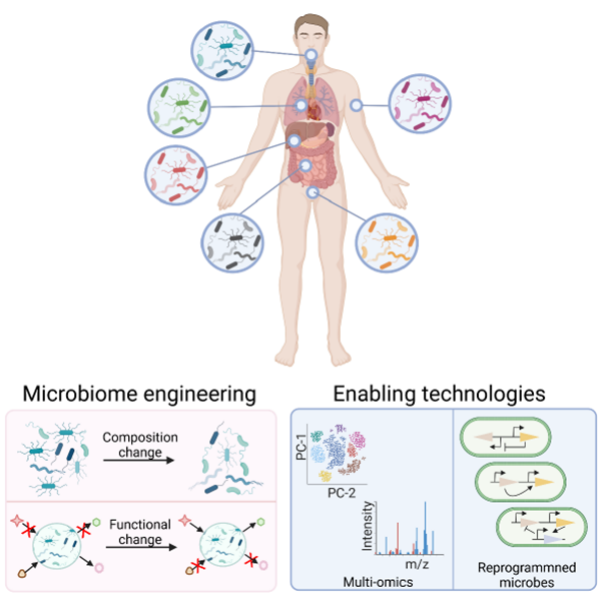

The human microbiome is composed of a collection of dynamic microbial communities that inhabit various anatomical locations in the body. Accordingly, the coevolution of the microbiome with the host has resulted in these communities playing a profound role in promoting human health. Consequently, perturbations in the human microbiome can cause or exacerbate several diseases. In this Review, we present our current understanding of the relationship between human health and disease development, focusing on the microbiomes found across the digestive, respiratory, urinary, and reproductive systems as well as the skin. We further discuss various strategies by which the composition and function of the human microbiome can be modulated to exert a therapeutic effect on the host. Finally, we examine technologies such as multiomics approaches and cellular reprogramming of microbes that can enable significant advancements in microbiome research and engineering.

## 1 Introduction

With advances that have enabled the sequencing of the whole genomes of organisms, we have since acquired an exponential amount of genome sequencing information from microbes. Over 130 000 complete or near-complete bacterial genomes have been sequenced. Meanwhile, there are more than 20 000 metagenomic projects publicly available, and many terabytes of sequencing data have been produced. This spectacular expansion of information regarding the genomic architecture of microbes has laid the foundation for truly revolutionary advances in our knowledge of microbial systems. We are now able to understand the interacting networks of biological molecules—including genes and proteins—at the systems level, and on the basis of this understanding, we can effectively engineer complex biological systems that perform desired functions. This technological advancement, along with the development of other key enabling techniques like gene synthesis, has contributed to the birth of the new interdisciplinary research field named synthetic biology.

However, microbes in the natural world are rarely found on their own; they invariably form a microbial community with each occupying a given niche. In addition, their habitats cover a wide range of abiotic and biotic environments. Through a long evolutionary cohabitation with the human body, this community of microbes, termed the microbiome, has established a profound role in its host’s physiological functions such as metabolism, immune development, and behavioral responses (section 2). Due to the intricate relationship between microbial communities and the living host, unsurprisingly a disruption in one often results in the disruption of the other. That is, a disturbed microbiome—known as dysbiosis—can be observed in an array of the host’s disease states, ranging from metabolic to immune and mood disorders. There has been a dramatic increase in human microbiome research and its association with different diseases in recent years. As the importance of the relationship between human-associated microbial communities and disease development becomes evident, there is a growing interest in engineering microbiomes to reshape and reprogram the composition and function of the gut microbiome as a novel therapeutic modality.

In general, modulating the function of the microbiome, or performing “microbiome engineering”, can be achieved by altering the gut microbial composition or its metabolomic function (section 3). Such alterations are reported to be largely mediated by providing a specific microbe (or consortia of microorganisms), prebiotics, or bioactive metabolites to elicit a change in the composition and functions of the microbiome to correct the disrupted metabolic function. In addition, engineered probiotics or synthetic consortia of microbes can be used to provide a more rational and precise therapeutic intervention. Since the early days of engineering probiotics for such interventions, various genetic tools have been identified and developed for the more precise and complex execution of therapeutic activities (section 4).

The goal of this Review is to provide a comprehensive understanding of advances in the microbiome–host relationship for human health. Additionally, this Review aims to provide a nonexhaustive list of studies covering the manipulation of the human microbiome to prevent or treat human disease, with a special focus on multiomics approaches and the cellular reprogramming of microbes to enable in-depth microbiome research and robust microbiome engineering.

## 2 Human Microbiome

Microbiome research has advanced rapidly over the past few decades and has now become a topic of great scientific and public interest. Historically, the field of microbiome research emerged from environmental microbiome research and later evolved into viewing eukaryotes as inseparable from the microbial community with which they share space. After all, the human body is an ecosystem where trillions of tiny organisms coexist with the host. The scientific term “microbiome” therefore refers to the set of genes of all microorganisms that inhabit almost all human body parts. The microbiome is thus considered as a second genome that has a symbiotic relationship with the host. This relationship may be positive or beneficial, negative or pathogenic, or neutral; hence, microbiome interactions play a key role in human health. The complex and diversified microbiome operates as a functional expansion of host genomes with an estimate of 50- to 100-fold more genes.^1^ These extra genes contribute to the regulation of host physiology by possessing various types of enzymatic proteins, influencing the produced metabolites and thus affecting host metabolism.^1^

Over the years, instead of looking into the relationship between one specific microorganism with its host, a holistic approach based on the holobiont theory has been applied.^2,3^ The beneficial interplay of the host and its microbiome is responsible for maintaining the host’s health, whereas disease development is often correlated with microbial dysbiosis, or a shift in the microbiota. As such, pathogens therefore represent only a tiny fraction of microorganisms, whereby the altered composition of the microbiome promotes the emergence and outbreak of pathogens.^2,3^ The vast majority of microbes are crucial for ecosystem functioning as well as beneficial interactions with other microbes, contributing to population dynamics and functional activities. Thus, opportunistic pathogens show that host–microbe interactions depend not only on the host but also on the entire microbiome.

The microbiota comprises all living members that form the microbiome, which encompasses bacteria, archaea, fungi, algae, and small protists. The members of microbiome also extend to viruses, phages, and mobile genetic elements—one of the most controversial inclusions in the definition of a microbiome.^4^ However, the microbiome has since been further defined to pertain to not only the community of microorganisms but also the whole spectrum of molecules produced by microorganisms, including their structural elements, metabolites, and molecules produced by the coexisting host.

Generally, microbial composition varies among different anatomical parts, and it is highly personalized as the microbiome’s composition also varies among individuals. The exact definition of a healthy microbiota has yet to be defined, but studies have shown that the use of probiotics, prebiotics, and synbiotics are beneficial by maintaining healthy body flora or by altering the microbiome toward a healthy microbial ecosystem.

Therefore, defining the core microbiota is crucial as it facilitates the discrimination of an intermittent or temporal microbiome that is affected by specific environmental conditions.^4^ The core microbiota is the microbial community that is constantly associated with a given host genotype or a specific environment, whereas transient microbiota changes over time. By identifying these differences, an appropriate experimental, methodological, and statistical design can be applied to refine the approach taken in microbiome studies for therapeutic applications.

### 2.1 Factors Influencing the Human Microbiome

Microorganisms reside in their preferred environment depending on their optimal growth conditions. They can be found on the human body’s external and internal parts as well as entrance sites. The external sites that house microorganisms include the skin, eyes, and even the exposed sites under the nails. The portals of entry for microorganisms are the respiratory tract (mouth and nose), gastrointestinal tract (oral cavity), urogenital tract, and breaks in the skin surface. Meanwhile, the internal parts of the body that are occupied by microbes include the lungs, gut, bladder, kidneys, and vagina.

Microbes tend to thrive in an environment that is suitable for them. Hence, these microorganisms are predicted to have mechanisms for adapting to conditions in the human microbiome that resemble their preferred natural environment. Environmental factors such as temperature, pH, oxygen concentration, pressure, osmolarity, and nutrient source contribute to the diversity and abundance of microorganisms at different sites of the body. For instance, our body temperature is optimal for housing many different types of microbes. Other factors, such as the presence of nutrient sources like sebum, change the skin’s pH and also act as a carbon source, facilitating the growth of certain groups of microbes.^5^ Interestingly, the dense layer of mucus that covers the intestinal epithelium not only serves as a carbon source for microbes but also provides attachment sites for bacterial adhesion.^6^

The abundance and diversity of the human microbiota is dependent on intrinsic and extrinsic factors. Intrinsic factors include the nature of body environments, as previously described, as the physiology of habitat sites facilitates the growth of some microbes. Other intrinsic factors that contribute to the microbiome’s composition include genetics, ethnicity, gender, and age. The human microbiome is generally stable and resistant once the microorganism has adapted to the environment. On top of intrinsic factors that may cause a shift in the microbiome over time, extrinsic factors such as diet, lifestyle, medication, geographic location, climate, and seasonality may cause changes to the microbial community. Moreover, the mode of delivery during birth has been shown to influence the microbiome. For example, newborns delivered via the vaginal versus Caesarean delivery possess different groups of dominating gut microbiome. However, at the age of 3, the gut microbiome changes to resemble that of the adult’s gut microbiome.^7^ As people reach beyond the age of 70, the ability to digest food and absorb nutrients in the gut changes, affecting the composition of the gut microbiome. With decreasing immune activity in older adults, this also contributes to changes in the overall microbiome as they are more susceptible to pathogens—thereby influencing the core microbiome. As *Bifidobacterium* spp. stimulates the immune system and metabolic processes, a decrease in Bifidobacteria may result in malnutrition and low systemic inflammatory status in older adults.^8^ Altogether, the human microbiome thrives in optimal growth conditions, depending on the natural environment of the body. When the natural environment of the body is altered, this results in microbial composition and diversity shifting to adapt to the changing environment, potentially resulting in disease.

### 2.2 Microbiota in Different Body Parts and Its Relationship with Health/Disease

#### 2.2.1 Digestive System

##### 2.2.1.1 Oral

The human oral cavity harbors one of the most versatile microbiomes, including bacteria, fungi, viruses, and protozoa, among others. There are two regions in the oral cavity colonized by microorganisms—dentures, or the hard surfaces of the teeth, and the soft tissue of the oral mucosa. The main bacterial genera of oral cavities include *Streptococcus*, *Granulicatella*, *Gemella*, *Actinomyces*, *Corynebacterium*, *Rothia*, *Veillonella*, *Fusobacterium*, *Prevotella*, *Porphyromonas*, *Capnocytophaga*, *Neisseria*, *Haemophilus*, *Treponema*, *Eikenella*, *Leptotrichia*, *Lactobacillus*, *Peptostreptococcus*, *Staphylococcus*, *Eubacterium*, and *Propionibacterium*.^9^ Meanwhile, predominant fungal genera include *Candida*, *Cladosporium*, *Saccharomyces*, *Fusarium*, *Aspergillus*, and *Cryptococcus*.^10^ Disease-related viruses such as mumps, rabies, and human papillomaviruses^11^ are also found in the mouth, as well as protozoa such as *Trichomonas tenax* and *Entamoeba gingivalis*.^12^

The oral cavity is the principal entry point to the human body, and thus, microbes residing in this area can potentially spread to different body sites and cause disease. The composition of the oral microbiome therefore plays a vital role in providing immunity for human health. For instance, nitrate metabolism by the microbiome reduces nitrate to nitrite. Nitrite is then converted to nitric oxide, which has an antimicrobial effect and is crucial for vascular health.^10^ Some oral microorganisms such as *Streptococcus salivarius* strain K12 contribute to host defense by creating an unfavorable environment that prevents the colonization of pathogenic bacteria. It produces a bacteriocin that restrains the growth of Gram-negative species associated with periodontitis disease.^13^

The most prevalent oral disease is dental caries, commonly known as tooth decay. The bacteria involved in dental caries are *Streptococcus mutans*, *Streptococcus sobrinus*, and *Lactobacillus acidophilus*. Other species such as *Veillonella*, *Bifidobacterium*, *Propionibacterium*, *Actinomyces*, *Atopobium*, and *Scardovia* have also been found to be associated with dental caries.^14,15^ Dental caries manifest when acid-producing bacteria residing in the oral cavity interact with the fermentable carbohydrate found in food. When the supragingival biofilm matures, it creates a low pH environment, demineralizing the tooth and eventually leading to cavitation.^16−18^ Without adequate oral hygiene, certain microorganisms produce pathogenic characteristics, causing gingivitis. When this condition persists through chronic bacterial infections, the subgingival plaque accumulation rearranges the microflora from a healthy to diseased state, affecting the gingiva and causing damage to the supporting connective tissue and the bone that fixes the teeth to the jaws.^17,19,20^

The oral microbiome has been recognized as a vital player in systemic health, with the disruption of the oral microbiome potentially contributing to several chronic diseases such as endocarditis, osteoporosis, and rheumatoid arthritis.^21−23^ Oral health has also been found to play a role in the development and progression of noncommunicable diseases (NCDs) such as obesity, diabetes, cancers,^24−26^ and neuropsychiatric disorders (NPDs).^27−30^ Thus, it has been proposed that the oral microbiome could potentially be used to assess the risk for certain diseases. Similar to the widely studied gut microbiome, oral microbiome research is shifting to a holistic, systems-level understanding of its functions and interactions with the human body.^31−33^ Future studies will likely shed light on how the oral microbiome can be restored to a healthy state.

##### 2.2.1.2 Gastric

The stomach was previously believed to be a sterile organ due to its inhospitality to bacteria. Such factors include its acidic environment, reflux of bile acids, thickness of the mucus layer, and conversion of food to nitrite by *Lactobacilli* present in the oral cavity, which then transforms into the antimicrobial nitric oxide. However, the lack of simple and reliable diagnostic tests has hampered the study of the gastric microbiome.^34,35^ With the discovery of *Helicobacter pylori* by Barry Marshall and Robin Warren in 1982, this notion has since been refuted. The most highly represented phyla in the gastric mucosa under normal conditions are Proteobacteria, Firmicutes (recently renamed to Bacillota^36^), Bacteroidetes (recently renamed to Bacteroidota^36^), Actinobacteria, and Fusobacteria.^37−39^ The gastric juice has a diverse microbial community that differs from the gastric mucosa. The dominating phyla in gastric juice are Firmicutes, Actinobacteria, and Bacteroidetes, whereas Proteobacteria and Firmicutes are dominant in the gastric mucosa.^37,40,41^ Furthermore, bacteria found in the oral cavity and duodenum such as *Veillonella*, *Lactobacillus*, and *Clostridium* can transiently colonize the stomach.^40,42^

Unsurprisingly, *H. pylori* is the predominant bacterium in the stomach of *H. pylori*-infected patients,^43^ and most *H. pylori* strains can modulate the gastric environment, thus altering the habitat of resident microorganisms.^44^ Furthermore, alterations in the gastric microbiome community can increase the risk for developing gastric cancer.^39^ It was also reported that eradicating *H. pylori* increased microbial diversity in the stomach.^45^ Even though interactions between *H. pylori* and commensal bacteria in the stomach are not fully understood, the discovery of its direct effect on the healthy gastric microbiome may shed some light on ways to modulate the gastric microbiome to prevent progression to severe disease.

##### 2.2.1.3 Intestines

The gut is the most densely and diversely colonized organ, with a bacterial-to-host cell ratio of 1:1. A vast majority of commensal bacteria reside in the colon, whereas a lower bacterial population is found in the stomach and small intestine. The main bacterial phyla present in the gut are Firmicutes and Bacteroides, which make up 90% of the gut microbiota.^46^ Other phyla that exist in the gut environment are Actinobacteria, Proteobacteria, Fusobacteria, and Verrucomicrobia.^46^ Notably, there are 200 different genera found under the Firmicutes phylum, with some examples including *Bacillus*, *Lactobacillus*, *Enterococcus*, *Clostridium*, and *Ruminococcus*. Although lactobacilli are beneficial to health, some Firmicutes species such as *Staphylococcus aureus* and *Clostridium perfringens* are harmful to the body when overgrown. Meanwhile, the predominant genera in Bacteroidota are *Bacteroides* and *Prevotella*. The less abundant Actinobacteria phylum is largely represented by *Bifidobacterium*, and this genus is known to have a positive impact on health. Under the Proteobacteria phylum, some well-known pathogens include *Enterobacter*, *Helicobacter*, *Shigella*, *Salmonella*, and *Escherichia coli*.

The composition of the gut microbiota changes at three stages in life: from birth to weaning; from weaning to obtaining a normal diet; and finally, during old age. Facultative anaerobes are the first to colonize the gut at birth, and these bacteria create anaerobic conditions that promote the growth of obligate anaerobes, starting with *Bifidobacterium* and *Bacteroides* spp., within 2 weeks.^47^ Infants born naturally are inoculated by the mother’s vaginal and fecal microbiota during birth, whereas those born by Caesarean section are initially exposed to the skin microbiota as well as the microbiome found in the environment.^48^ At 3 days, infants who were naturally delivered possessed a greater abundance and variety of *Bifidobacterium* spp. than the Caesarean-born babies.^49,50^ Moreover, babies who were solely breastfed until weaning were observed to generally have a more stable and less diverse bacterial community, with higher proportions of bifidobacteria than babies fed by formula milk.^51−54^ After being introduced to solid food, their gut microbiome diversified and the abundance of Firmicutes increased.^54−56^ The microbiomes of breastfed and formula-fed babies become indistinguishable by around 18 months of age. By the age of 3, their microbiomes resemble that of an adult.^7,54^ At old age, there is reportedly a decline in microbiota diversity, with reduced numbers of Bifidobacteria and an increase in Enterobacteriaceae.^57,58^ Likewise, the abundance of Bacteroidetes increases, whereas the Firmicutes becomes less abundant in elderly adults (>65 years).^59^

Apart from age, gut microbiome composition is also greatly influenced by the environment in different anatomical locations. The large intestine has slow flow rates, and the pH level ranges from mildly acidic to neutral. Thus far, it comprises the largest microbial community dominated by obligate anaerobes. The large intestine comprises several microenvironments wherein microorganisms reside. The epithelial surface and inner mucin layer harbors minimal colonization during the healthy state, whereas the diffuse mucin layer has specialist colonizers such as *Akkermansia muciniphila*. The liquid phase of the gut lumen comprises a diversity of microorganisms and specialized primary colonizers like *Ruminococcus* spp. depending upon the dietary fibers found in the gut lumen.^60^ Given that the small intestine has a fairly short transit time of ∼3–5 h in digestion, the presence of high bile concentrations that possess antimicrobial activity^61^ makes the small intestine a challenging environment for microbial colonizers.^61^ Molecular analysis has revealed that the jejunal and ileal components comprise mainly facultative anaerobes, including the bacterial phyla Proteobacteria and Bacteroides and the *Streptococci*, *Lactobacilli*, and *Enterococci* species.^62,63^

Gut bacteria are crucial for regulating digestion along the gastrointestinal tract. The commensal bacteria play a key role in processing nutrients and metabolites such as short-chain fatty acids (SCFAs), bile acids, amino acids, etc.^64^ By doing so, some of these bacteria facilitate host energy harvesting and metabolic efficiency.^65^ Some of these members also play an important immune function against pathogenic bacteria and prevent bacterial invasion by maintaining intestinal epithelium integrity.^66^ Although the composition of the microbiome species performs a key role in metabolism, the community’s metabolic output is also dependent on the availability of substrates to the microbiota^67,68^ or when extrinsic factors such as diet influence the gut microbiome. Microbe-synthesized metabolites potentially mediate crosstalk between the metabolic, immune, and neuroendocrine systems, thus governing host wellness.^69^

In addition to regulating digestion, dominant, nonpathogenic gut microorganisms occupy a specific niche, suppressing pathogenic colonization and growth. However, when the balance of the gut microbiome is perturbed, gut permeability increases. This change in permeability allows opportunistic pathogens to invade and colonize empty niches, changing the gut environment. This may lead to the production of dysregulated metabolites that are potentially harmful to the host, causing a range of diseases. Increased gut permeability also permits the entrance of microbe-derived products such as metabolites, virulence factors, and other luminal components, disrupting the gut microbiome’s normal function and contributing to aberrant immune-inflammatory responses such as inflammation, allergy, and autoimmune disorders mediated by molecular mimicry and a dysregulated T cell response.^70^

Sometimes the source of the opportunistic pathogens comes from the resident site of the microbiome, and this occurs when the healthy nondisease state of the gut microbiome is disturbed, causing the failure of colonization resistance against the pathogenic member. An example is *Clostridium difficile*, which exists in the normal gut microbiota but becomes pathogenic when the healthy nondisease microbiome state is disrupted. *C. difficile* may damage the cytoskeleton and colonic epithelial barrier integrity, inducing aberrant inflammatory response and cell death.^71^*C. difficile* infection (CDI)-associated symptoms include diarrhea, pseudomembranous colitis, sepsis, and death.^71^ It is proposed that the dominant gut microbiota in the healthy nondisease state confers protection to the host by preventing the overgrowth of *C. difficile* as it is often related to antibiotic-associated diarrhea compared to other pathogens such as *Salmonella* species.^72,73^

Another commonly studied gut microbiome-associated disease is inflammatory bowel disease (IBD). It is a group idiopathic, chronic, and relapsing gastrointestinal inflammation with two common forms: ulcerative colitis (UC) and Crohn’s disease (CD).^74^ Inflammation occurs at any location along the entire GI tract in CD. Meanwhile in UC, inflammation is restricted to the large intestine. Both conditions are associated with recurring fever, diarrhea, and abdominal pain. It was suggested that dysbiosis in the gut potentially contributes to IBD pathogenesis.^75^ An example is a reduction in the abundance of Firmicutes such as *Faecalibacterium prausnitzii* and *Roseburia* spp.^76−78^ These are butyrate-producing bacteria, with butyrate being the primary energy substrate for colonocytes. Thus, a decrease in Firmicutes could heighten local inflammation by decreasing anti-inflammatory cytokines.^78,79^ As such, *F. prausnitzii* has been explored as a probiotic for therapeutic use.^76^

Aside from IBD, other intestinal disorders associated with the dysbiosis of the gut microbiota include irritable bowel syndrome (IBS), celiac disease, and colorectal cancer (CRC). A study of fecal samples from IBS patients exhibited a significant reduction in the concentration of *Lactobacillus* species as compared to healthy controls.^80^ Other studies have revealed that there is an increase in the ratio of Firmicutes to Bacteroidetes in IBS patients as compared to healthy individuals.^81,82^ There was also a decrease in some Firmicutes families such as *Lactobacilli* and *Faecalibacterium*, as well as *Bifidobacteria* and *Collinsella* under the Actinobacteria population. In IBS patients, there was an increase in abundance in some Firmicutes families (*Veillonella*, *Streptococci*, and *Ruminococcus* spp.) and in Proteobacteria (*Enterobacteriaceae* spp.). These findings reveal that there is a loss of microbes associated with epithelial barrier function in IBS patients.^81,82^ Although many diseases are hypothesized to have an association or correlation with the microbiome, some studies have also suggested the causation factors of the disease based on microbial activities. With this information, therapeutics advances can be developed.

Other microorganisms that live in the gut are viruses and bacteriophages that make up the vast majority of gut microbiota’s viral components. Dominant archaeal species such as *Methanosphaera stadtmanae* and *Methanobrevibacter smithii* are also found in the gut microbiome.^83^ Longitudinal studies of the gut have shown that specific species of an individual’s microbiota are very stable and persist for a year or more.^84,85^ The specific communities of human gut microbiomes are influenced by interindividual and intraindividual variation throughout the life cycle. Examples of some factors that affect variations in the microbiome include the intestine’s anatomical regions, mode of delivery, method of milk feeding, weaning period, age, diet, and antibiotic treatments. The gut environment varies between different anatomical regions in terms of physiology, digesta flow rates, substrate availability, host secretions, pH, and oxygen tension.

#### 2.2.2 Respiratory System (Nasal, Airway, and Lungs)

##### 2.2.2.1 Nasal

The nasal cavity is an essential interface to the external environment. During inhalation, the airways are exposed to the environment, which comprises microorganisms, pollutants, aeroallergens, and more. A wide variety of potential pathogenic and harmless bacteria reside in the nose, and this diversity may be attributed to localized factors such as temperature and humidity. The position in the respiratory tract may also contribute to the diversity of the nasal microbiome. For instance, the anterior nares have decreased levels of microbiome biodiversity in comparison to the middle meatus and sphenoethmoidal recesses. The anterior nares are lined with keratinized squamous epithelium and sebaceous glands that produce sebum and may impact bacterial diversity.^86^ However, a recent study did not detect any significant differences in bacterial diversity among the middle meatus, inferior turbinate, and anterior nares from healthy individuals,^87^ and thus, further studies may be required to obtain comparable information.

The microbiome of the anterior nares in healthy adults has been observed to be dominated by three phyla: Actinobacteria, Firmicutes, and Proteobacteria.^88^ The anterior nares are further classified into four distinct genus profiles comprising *Staphylococcus*, *Propionibacterium*, *Corynebacterium*, or *Moraxella*.^89^ The middle meatus possesses a high abundance of *Staphylococcus aureus*, *Staphylococcus epidermidis*, and *Propionibacterium acnes*.^90^ The nasal microbiome in the unhealthy disease state has not been well-characterized, making further research necessary. Thus far, *Staphylococcus aureus* has been identified as one bacterial species that potentially functions in the development of the nasal disease chronic rhinosinusitis (CRS). Colonization of the nasal cavity and sinus with *S. aureus* may be associated with the presence of nasal polyps or disease severity in CRS.^91^ An increased abundance of *S. aureus* has been observed in CRS participants with nasal polyps, compared to participants without the polyps.^90^ With this preliminary information in hand, further studies on the clinical relevance of the nasal microbiome in CRS and the functional role of *S. aureus* in CRS development should be explored in future research.

##### 2.2.2.2 Pharynx, Larynx, and Trachea

The respiratory tract has long been thought to be sterile, largely due to the difficulty of culturing bacteria from the tract. However, microbes from the environment may first enter the upper tract (pharynx and larynx) followed by the lower tract (trachea) through the oral or nasal routes. As such, the upper respiratory tract has a greater abundance of bacteria compared to the lower region.^92,93^ Given the current ease of sample collection, future respiratory tract microbiome studies may be explored further to obtain a consistent microbiome among healthy individuals. Nevertheless, studies have shown that healthy individuals have a lower abundance of Proteobacteria as compared to patients with mild asthma.^94^ It was also reported that asymptomatic neonates whose throats are colonized with *Streptococcus pneumoniae*, *Haemophilus influenzae*, or *Moraxella catarrhalis* are at an increased risk for recurrent wheezing and asthma early in life.^95^ These bacteria have consistently been associated with exacerbations of both asthma^96^ and chronic obstructive pulmonary disease (COPD).^97^ So far, there are still limited studies on the respiratory tract microbiome, and further research is required.

##### 2.2.2.3 Lungs

In many textbooks, it is commonly held that the lungs are normally sterile. However, during respiration, the lungs are continuously exposed to a wide range of environmental microbes. In the past, incompatible culture conditions have led to the absence of bacteria in respiratory specimens, supporting the misinterpretation that healthy lungs are free of bacteria.^98^ The invasive procedures involved in obtaining clinical samples also contributed to the delay in the systemic investigation of the lung microbiome.^98^ While the most commonly used approach to study bacterial communities is via high-throughput sequencing of amplicons of the 16S rRNA gene, this technique presents technical challenges when bacteria with a low biomass are unable to mask any potential contaminants.^98^ Healthy lungs contain a highly diverse interkingdom community of bacteria including *Prevotella*, *Veillonella*, *Streptococcus*, *Haemophilus*, *Neisseria*, and *Corynebacteria*.^99−101^ In addition to these, many viruses such as *Adenovirus*, *Rhinovirus*, influenza, Epstein*-*Barr, and measles, among others, as well as fungi species (*Aspergillus* spp., *Candida albicans*, *Candida immitis*, *Candida neoformans*, etc.) are also associated with the respiratory tract.^98^

In every lung disease, the composition of the lung microbiome is altered compared to healthy controls. It is unknown if an altered lung microbiome drives the progression of lung disease or if it is a secondary consequence of the altered growth environment of the lungs. In some disease states, an increased airway wall permeability and mucus production introduces nutrient supply to the normally sparse lung environment. The mucus introduces pockets of increased temperature and decreased oxygen tension, selectively favoring the growth of disease-associated microbes.^102,103^ In the event of enhanced immunogenicity, the airways and alveoli are exposed to pathogen-associated molecular patterns and microbial metabolites that provoke further inflammation, which in turn further alters airway conditions.^104^ The generation of intraalveolar catecholamines and inflammatory cytokines promotes the growth of select bacterial species such as *P. aeruginosa*, *S. pneumoniae*, *Staphylococcus aureus*, and *Burkholderia cepacia* complex, whereas the recruitment and activation of inflammatory cells kills and clears bacteria with variable, species-specific effectiveness.^105−108^

It has been proposed that respiratory exacerbations are acute events of respiratory dysbiosis—that is, the disorder and dysregulation of the respiratory ecosystem—accompanied by a dysregulated host immune response, eliciting negative effects on the host.^98^ This is supported by a study that found that bacterial communities in the patients’ airways shift away from Bacteroidetes—the most abundant phylum in healthy subjects—toward Proteobacteria and other disease-associated bacteria at the time of exacerbation.^109^ Exacerbations are activated by an inflammatory state that initiates a cascade of inflammatory responses that escalates the dysbiosis–inflammation cycle, and homeostasis is only restored after the disconnection of the positive feedback loop.^98^

#### 2.2.3 Skin

The skin is the largest and most exposed organ in the human body. Despite having plenty of transient interactions with the environment, the composition of the skin microbiota remains surprisingly stable. The diversity and relative abundance of the skin’s microbiome varies among individuals and the physiology of the skin sites. Generally, the microbial community has been categorized into three broad groups: oily, moist, and dry.^110^ In some cases, “feet” is separately categorized from the three broad groups because it has a distinct microbial signature and is in regular contact with the ground, constituting unstable microflora.^111^

These characteristics create many possibilities for the skin to house numerous commensal bacteria, fungi, viruses, archaea, and mites.^110^ They exist in different compositions and densities at various skin sites, and altogether these microorganisms are defined as the skin microbiome. The composition and abundance of microorganisms are dependent on the physiology of the skin site. For healthy adults, sebum-rich sites were dominated by lipophilic *Cutibacterium* (formerly *Propionibacterium*) species, whereas bacteria such as *Staphylococcus* and *Corynebacterium* species thrive in humid and moist areas such as the armpit, bends of the elbow, and feet.^112−115^ In contrast to bacteria, the fungal community was not affected by the physiology of the skin. As such, the predominant fungi at the core body and arm sites are the genus *Malassezia*, while the feet’s skin is colonized by a diverse community of *Malassezia* spp., *Aspergillus* spp., *Cryptococcus* spp., *Rhodotorula* spp., *Epicoccum* spp., and others.^115,116^ Across skin sites, bacteria were more abundant compared to fungi; however, as there are less fungal reference genomes compared to bacteria, this may partly contribute to the difference in the abundance.^110^ Unlike bacteria and fungi, the colonization of eukaryotic viruses is not dependent on the anatomical site.^117^ Currently, studies on the interaction of the skin virome with the host and bacteriophages is limited and will benefit from future research. For instance, a study has revealed that a eukaryotic virus may cause a rare but aggressive form of skin cancer.^118^ In contrast, bacterial and fungal communities found at sebum-rich areas were found to be the most stable, whereas those at the foot sites were the least.^117,119^ This instability may be due to the transient presence of fungi in the environment.^110^ Eukaryotic DNA viruses, on the other hand, varied the most over time.^117,119^

The skin has comparatively less nutrients compared to the nutrient-rich environment of the intestines, with its available resources comprising sweat, sebum, and the stratum corneum.^120^ As such, this promotes *Propionibacterium acnes* to thrive in the anoxic sebaceous gland.^121^ This facultative anaerobe also utilizes proteases to obtain amino acids from skin proteins,^121^ as well as lipases to degrade triglycerides that retrieve free fatty acids, facilitating bacterial adherence.^122−125^ In facial samples, the abundance of *Propionibacterium* spp. positively correlates with the cheek’s sebum levels.^126^ Interestingly, auxotrophic species such as *Malassezia* and *Corynebacterium* employ the lipids found in sebum and from the stratum corneum as they are unable to produce their own lipids for certain functional roles.^120^ Thus, this may be one reason for the dominance of the *Malassezia* species in the adult skin mycobiome.^110^ Likewise, *Staphylococcus* spp. harbors strategies for surviving on the skin, including halotolerance and utilizing urea found in sweat as a nitrogen source.^120^*Staphylococcus* spp. also produces proteases that retrieve nutrients from the stratum corneum and adherens that facilitate skin adhesion.^120^

Similar to the association of age with the gut microbiome, the skin microbiome is also significantly affected by age. During puberty, the increased level of hormones stimulates the sebaceous glands to produce additional sebum. This results in the skin of postpubescent individuals favoring the growth of lipophilic microorganisms such as *Propionibacterium* spp., *Corynebacterium* spp.,^127^ and fungal *Malassezia* spp.^128,129^ On the other hand, prepubescent children have a higher abundance of Firmicutes (*Streptococcaceae* spp.), Bacteroidetes, and Proteobacteria (betaproteobacteria and gammaproteobacteria) as well as a more diverse fungal community.^127,128^ This reflects the association between one’s age and the skin microbiome and, hence, relates to the tendency to develop certain diseases at different ages. For instance, in prepubescent children, cases of atopic dermatitis related to *Staphylococcus* dropped, whereas *Malassezia*-related tinea versicolor is more prominent in adults as compared to children.^130−132^

To prevent colonization by pathogens, the skin’s resident microbial members interact with each other. However, in some conditions, bacteria that were originally beneficial may exhibit pathogenicity associated with changes in the microbiota, otherwise known as dysbiosis. For example, the bacterium *P. acnes*, the most abundant microorganism present in the skin of healthy adults, is associated with the acne vulgaris commonly seen among teenagers.^133,134^ Even though *P. acnes* is present in almost all adults, only a minority have acne issues, indicating that the gene expression profile varies at the functional level and that skin physiology—such as the level of sebum production and its secretion rate—correlates with the severity of clinical symptoms.^135,136^ In addition, it was reported that the presence of *P. acnes* in the follicles and its formation of biofilms are associated with acne development.^137^

*S. aureus* is commonly cultured from the skin of individuals with atopic dermatitis (AD),^138^ also known as eczema. There are factors supporting the hypothesis that the skin microbiome has an influential role in disease pathogenesis. In the event of AD flares, it was demonstrated that there is a decline in microbiome diversity and a dramatic increase in the abundance of *S. aureus* compared to the healthy or postflare state.^139−141^ Additionally, the relative abundance of staphylococci advanced closely with the severity of the AD flare. Even though the correlation of *S. aureus* with AD during active disease exacerbation is known, the functional role of staphylococci in driving disease states is still poorly understood. Furthermore, it is also unknown if *S. aureus* contributes to disease initiation due to dysbiosis or if the changes in the microbial community are a consequence of the disease state.

#### 2.2.4 Urinary System

The urinary bladder was traditionally considered sterile as any bacteria found in the bladder was assumed to be pathogenic. However, with the discovery of the existence of nonpathogenic microbes in the human body, this notion has been abolished.^142^ Due to advances in sampling and DNA sequencing techniques, commensal microbes have been identified in the urinary tract.^143^ However, research on the urinary microbiome, or the urobiome, remains limited and understudied. In general, the abundance and diversity of the microbiome in the urine is lower compared to the gut by ∼10^6^–10^7^ times.^144^ The detection of the urobiome remains limited by the sampling method used. For example, some bladder mucosa-associated bacteria are undetectable in urine samples, and invasive methods are necessary for detection. The urobiome is similar for both genders, and the majority of the bacteria found belong to the phylum Firmicutes. Other phyla found in the urobiome are Actinobacteria, Bacteroidetes, and Proteobacteria.^145^ Common genera for both genders are *Escherichia*, *Enterococcus*, *Prevotella*, *Streptococcus*, and *Citrobacter*.^146^*Pseudomonas* was detected only in males, whereas *Corynebacterium* and *Streptococcus* were more abundant in males compared to females.^146,147^ On the other hand, the abundance of *Lactobacillus* was found to be higher in females compared to males.^146,147^ Even though *Lactobacillus* is generally known as a probiotic, some species are associated with certain pathologies. For instance, *Lactobacillus gasseri* is associated with urgency urinary incontinence (UUI).^144^ Moreover, a decrease in the abundance of *Lactobacillus* facilitates the colonization of disease-causing uropathogens.^147^*Gardnerella* is second to *Lactobacillus* in terms of abundance among the urobiome in females. The most abundant species is *Gardnerella vaginalis*, with some pathogenic strains, causing urinary tract infections (UTIs) in women, which is comparatively less frequent in men.^144,148^ In general, the dominant genera found in the female urinary microbiome are *Atopobium*, *Citrobacter*, *Enterococcus*, *Escherichia*, *Gardnerella*, *Lactobacillus*, *Prevotella*, *Shigella*, *Sneathia*, and *Streptococcus*, with the dominating species exclusive to healthy women being *Lactobacillus crispatus*, *Gardnerella vaginalis*, and *Atopobium vaginae*.^149^ However, reports on the male urobiome are significantly fewer compared to the female urobiome, and the small sample size may hinder the identification of differences in the urobiome of both populations.^150^ Finally, in healthy males, it is known that *Staphylococcus haemolyticus* is an abundant species.^151^

The anatomical proximity and physiology of body sites influences the microbial community and their abundance. Unlike males, the proximities between the opening of the reproductive organ and the urinary tract are closer to each other in females. Thus, the vagina might be the main source of the microbial community in the urinary tract. In two studies, the existence of a common urogenital microbiota in both vaginal and urine samples was reported.^152,153^ However, some differences were also observed. For instance, the genera *Tepidimonas* and *Flavobacterium* were found to be present in the urobiome, even though they are absent in the vaginal microbiome.^153^ Other urobiomes such as the urinary fungal community have not been well-characterized, though the presence of *Candida* spp. has been reported in healthy individuals.^144^ To date, only one species of archaea (*Methanobrevibacter smithii*) has been reported to be associated with urinary infection.^154^ A urinary virome has also been detected, including lytic bacteriophages such as a *Pseudomonas aeruginosa*-infecting phage isolated from kidney stones^155^ or *Escherichia coli*-infecting phages isolated from the bladder of females suffering from UUI.^156^

UTIs are one of the most common bacterial infections found in humans, especially among women due to the design of the female anatomy. UTIs have been commonly associated with *Escherichia coli*, but other commensal members are found in the gut microbiota, such as *Enterococcus* and *Staphylococcus*.^157^ Interestingly, there seems to be a correlation between an increase in the intestinal abundance of these genera and a higher prevalence of UTI.^158,159^*E. coli* is also part of the commensal urobiome, and hence, it has been detected in healthy individuals. However, there are some differences in the motility genes between the isolates found in UTI patients and those in healthy individuals.^160^ Moreover, *E. coli* has greater pathogenicity when it is isolated together with *Enterococcus*; however, the mechanisms underlying this coinfection are not yet well-understood.^161,162^

The vaginal microbiota may also impact the host’s susceptibility to UTI. For instance, women with recurrent UTI become resistant if their vaginal microbiome is altered by the administration of probiotics, especially *Lactobacillus crispatus*.^163^ Furthermore, women with bacterial vaginosis caused by the overgrowth of anaerobic species such as *Gardnerella vaginalis* suffer more UTIs than women with microbiomes composed mainly of *Lactobacillus*.^164^ Studies have shown that temporary exposure to some strains of *Gardnerella vaginalis* triggers the activation of *E. coli* from dormant intracellular reservoirs in the bladder, enhancing the chance of developing recurrent UTI through the induction of apoptosis and interleukin 1-receptor-mediated injury in bladder epithelial cells.^165^ These results extend the classic concept of UTI pathogenesis, suggesting that the disease may be driven by occasional exposures of the urinary tract to gut or vagina-associated bacteria that are not traditionally considered as uropathogenic.

#### 2.2.5 Reproductive System

##### 2.2.5.1 Vaginal

The human vaginal microbiome differs from other body sites as it is dominated by a single genus, *Lactobacillus*.^166−168^ Because *Lactobacillus* spp. lower vaginal pH, they inhibit the growth of many pathogens and beneficially impact the host epithelium, modulating the immune system.^169−172^ It was reported that ∼25% of women in North America possess vaginal microbiomes that are not dominated by *Lactobacillus*.^173^ Instead, their microbiomes are composed of an even population of obligate and facultative anaerobes–namely, species in the genera *Gardnerella*, *Prevotella*, *Atopobium*, *Sneathia*, *Megasphaera*, and *Peptoniphilus*.^166,168,173−175^ Interestingly, having such a vaginal microbiome correlates with the higher tendency of being diagnosed with bacterial vaginosis (BV),^176,177^ a bacterial infection resulting from the imbalance of beneficial and harmful bacteria. Thus, epidemiological studies have associated microbiomes that are not dominated by *Lactobacillus* with an increased risk of acquiring sexually transmitted infections (STIs)^178−181^ and preterm birth.^182−186^ This also suggests that having a non-*Lactobacillus*-dominated community may be less protective toward developing adverse health outcomes.^187^

The vaginal epithelium is coated in a cervical mucus layer that is regulated by hormones.^188^ The mucus is composed of protein, lipids, water, and glycoproteins, also referred to as mucins.^189^ Mucins have been hypothesized to possess a protective role in the vaginal epithelium and may also serve as a nutrient source for the vaginal microbiome.^190−193^ Mucin levels change throughout the menstrual cycle and similarly, the level of glycogen fluctuates throughout the cycle too.^194−196^ Glycogen is produced by the vaginal epithelium, and epithelial cells consist of a high level of glycogen compared to other epithelial tissues.^197^ Similar to mucin, glycogen is also thought to be a nutrient source for the vaginal microbiome.^198,199^ The characteristics of vaginal physiology are influenced by hormonal changes. Therefore, during menopause, the levels of cervical mucus and glycogen decline, and the usual acidic environment of the vagina changes, contributing to the modified microenvironment for the vaginal microbiome.^200^

Vaginal microbiota with a lower abundance of *Lactobacillus* and a higher proportion of facultative and obligate anaerobes such as *Gardnerella*, *Prevotella*, *Atopobium*, and *Sneathia* are associated with acquiring diseases like STIs and human immunodeficiency virus.^178,201^ This vaginal microbiome profile has also been linked to both the incidence and prevalence of human papillomavirus.^202,203^ Despite continuous research seeking to establish the association between vaginal microbiota and health, there is still insufficient information for connecting casual mechanisms and pathways. Nevertheless, an exploratory study that used vaginal microbiota transplants (VMTs) has demonstrated long-term remission of women with recurrent bacterial vaginosis,^204^ and such an approach may be employed in the future to gain insights into the modulation of the vaginal microbiome for therapeutic purposes.

### 2.3 Summary

In this section, we described how the large and diverse groups of microorganisms that reside in various parts of the human body (Table 1) have a highly coevolved relationship with human health. Microbiome research has highlighted the importance of human-microbiota ecosystems in the promotion of health and various disease-causing processes. This also suggests that the microbiome is a potential target for disease management. In the following section, we will present various strategies by which the composition and function of the microbiome can be modulated for therapeutic outcomes. With more studies revealing the mechanistic insights of the microbiome in relation to health, therapeutics applications can be refined.

**Table 1:** Predominant Microbiome Present on Different Body Sites and Their Relationship with Disease

body site	predominant microbes	microbiome-associated diseases
mouth	bacterial phyla: Actinobacteria, Bacteroidetes, Firmicutes, Fusobacteria, Proteobacteria, and Spirochaetes	dental caries (*Streptococcus mutans*, *Streptococcus sobrinus*, and *Lactobacillus acidophilus*)^14,15^
	fungal genera: *Candida*, *Cladosporium*, *Saccharomycetales*, *Fusarium*, *Aspergillus*, and *Cryptococcus*	periodontitis (*Streptococcus salivarius* may reduce the disease development)^13^
stomach	bacterial phyla: Proteobacteria, Firmicutes, Actinobacteria, Bacteroidetes, and Fusobacteria	gastric cancer (*Helicobacter pylori*)^39^
intestines	bacterial phyla: Firmicutes and Bacteroides	inflammatory bowel disease (lower abundance of Firmicutes)^76−78^
	archaeal species: *Methanosphaera stadtmanae* and *Methanobrevibacter smithii*	irritable bowel syndrome, celiac disease, and colorectal cancer (reduction in *Lactobacillus* species)^80^
nose	bacterial phyla: Actinobacteria, Firmicutes, and Proteobacteria	chronic rhinosinusitis (*Staphylococcus aureus*)^91^
airway and lungs	bacterial phyla: Firmicutes, Proteobacteria, and Bacteroidetes	asthma (lower abundance of Proteobacteria)^94^
	fungal species: *Candida albicans*, *Ceriporia lacerata*, *Saccharomyces cerevisiae*, and *Penicillium brevicompactum*	chronic obstructive pulmonary disease (*Streptococcus pneumoniae*, *Haemophilus influenzae*, or *Moraxella catarrhalis*)^95^
	viruses: *Herpesviridae*	
skin	bacterial phylum: Actinobacteria, Firmicutes, Bacteroidetes, and Proteobacteria	atopic dermatitis (*Staphylococcus aureus*)^138^
bladder	bacterial phylum: Firmicutes	urgency urinary incontinence (*Lactobacillus gasseri*)^144^
		urinary tract infection (*Gardnerella vaginalis*)^144,148^
vagina	bacterial phylum: Firmicutes (*Lactobacillus*)	bacterial vaginosis, sexually transmitted infections (not dominated by *Lactobacillus*)^173,178−181^

## 3 Strategies To Engineer the Microbiome for Therapeutic Applications

As described in section 2, the human microbiome plays a crucial role in health maintenance as it can influence the development of various diseases. This knowledge has led to the emergence of new therapeutic approaches that target both acute and chronic diseases by modulating the host microbiome. The rapid increase in the availability of robust, broad-spectrum, and easy-to-use synthetic biology tools (as discussed in section 4) has further contributed to unlocking the potential of engineering the microbiome to prevent and treat diseases.

In this section, we present different methods by which the human microbiome can be rationally engineered. We also discuss examples that demonstrate that microbiome engineering is a viable way to target diseases and enhance human health.

### 3.1 Changing the Population Dynamics of the Microbiome

The composition of the human microbiome is unique to every individual and is constantly fluctuating due to factors such as age, diet, host genetics, and medication. Nevertheless, distinct microbiome profiles have been associated with specific diseases by comparing the differences between patients and healthy controls. These differences can occur at any level of the taxonomic rank, with previous reports showing phylum-level to species-level associations.^205^ Several methods have been applied to correct microbiome differences with varying specificity and magnitude, as discussed later.

#### 3.1.1 Increasing the Abundance of Specific Members of the Microbiome

A low microbial diversity of the human microbiome is significantly associated with several diseases.^206^ However, the changes observed in the microbiome composition might be dissimilar in different populations. For example, Dutch and Belgian cohorts showed a negative correlation between the Bacteroidetes enrichment and diversity,^207^ whereas a positive correlation was observed between Bacteroidetes and diversity in African individuals,^208^ underscoring the need for population-specific comparisons of the microbiome between healthy individuals and patients. The ratio of the two most dominant phyla in the microbiome, namely, Firmicutes/Bacteroidetes, is one of the most important parameters representing microbiome diversity, at least in the gut. Previous reports have shown a high ratio of Firmicutes/Bacteroidetes in obese Ukrainian adults compared to their lean counterparts.^209^ A similar observation was also made in Dutch^210^ and Japanese^211^ individuals with a systematic review of 32 studies across varied populations, confirming the positive correlation between the Firmicutes/Bacteroidetes ratio and obesity.^212^ In contrast, a decreased Firmicutes/Bacteroidetes ratio has been observed in patients with inflammatory bowel disease (IBD). Manichanh et al. reported a significant reduction in the proportion of Firmicutes in the microbiome of patients with Crohn’s disease (CD) compared to the healthy microbiome.^213^ The alteration in the gut microbiome was also associated with disease activity and severity, with lower Firmicutes observed in patients with active ulcerative colitis (UC) compared to the inactive disease and in aggressive CD compared to the nonaggressive disease.^214^ Firmicutes in the gut, particularly the genus *Faecalibacterium*, were also reduced in patients with major depressive disorder and bipolar disorder,^215^ as well as chronic fatigue syndrome.^216^ Apart from Firmicutes and Bacteroidetes, a lower abundance of other phyla in the gut, such as Actinobacteria, have also been associated with several diseases. *Bifidobacterium* is one of the most important genera belonging to the Actinobacteria, with lower counts of bifidobacteria found in celiac disease,^217^ irritable bowel syndrome,^218^ and Alzheimer’s disease.^219^

The correlation between the decrease in microbiome diversity and disease development is not only limited to the gut; it has been observed in other anatomical locations as well. Kong et al. reported reduced skin microbiome diversity in patients with atopic dermatitis, with an enrichment of *Staphylococcus* sequences and depletion of Actinobacteria.^220^ In the lung, reduced microbial diversity and an abundance of Firmicutes was found to be significantly associated with the progression of idiopathic pulmonary fibrosis.^221^ The depletion of *Lactobacillus* spp., predominant members of the healthy urine microbiome in females, was observed in patients with UUI,^144^ a predisposition to UTI,^222^ and overactive bladder.^223^

##### 3.1.1.1 Probiotic Supplementation

The studies mentioned earlier demonstrate that decreased levels of specific phyla or genera of microbes in the microbiome are significantly associated with disease development and progression. Therefore, engineering the microbiome to correct this imbalance is imperative to alleviate associated disorders and promote health. This can potentially be achieved by exogenously supplementing beneficial bacteria, such as probiotics, which can rebalance the microbiome. Such strategies have been evaluated previously and were found to be successful in some cases.

Joung et al. studied the effect of oral administration of *L. plantarum* K50 or *L. rhamnosus* GG to obese mice on a high-fat diet for 12 weeks.^224^ At the end of the intervention, they showed that treated mice had reduced body weight and serum triglyceride levels as well as increased high-density lipoprotein cholesterol levels. A high Firmicutes/Bacteroidetes ratio was seen in nontreated obese mice, as seen in obese human individuals, which significantly reduced after treatment with the probiotic strains.^224^ In another study, the administration of *L. rhamnosus* GG to mice on a high-fat diet led to the reversal of resistance to leptin (an appetite-regulating hormone), an increase in fecal microbiome diversity, and a reduction in the Proteobacteria phylum.^225^ A meta-analysis of 15 clinical trials comprising a total of 957 participants concluded that probiotic intervention resulted in a significant reduction of body weight, fat percentage, and body mass index but not fat mass when compared to the placebo.^226^ Although most clinical trials conducted did not perform microbiome analyses, changes in the composition of the fecal microbiome post-*L. salivarius* Ls-33 administration in obese adolescents were reported by Larsen et al., with a significant decrease in the Firmicutes/Bacteroidetes ratio.^227^ However, the study did not find any changes in anthropometric and inflammatory parameters,^228^ making the correlation between changes in the gut microbiome and obesity ambiguous in humans.

Apart from the commonly used *Lactobacillus* and *Bifidobacterium* strains, *Akkermansia muciniphila* has found wide applications in treating cardiometabolic diseases, including obesity and diabetes. *A. muciniphila* is one of the most abundant species of the human gut microbiome; its depletion has been reported in obese and diabetic mice and, more importantly, in humans with pathologies such as obesity, type 2 diabetes, hypertension, and IBD.^229^ Due to this clear negative correlation between *A. muciniphila* and cardiometabolic diseases, the safety and efficacy of *A. muciniphila* supplementation in counteracting obesity and diabetes was evaluated in a randomized, double-blind, placebo-controlled study with 40 overweight or obese individuals.^230^ It was observed that, compared to the placebo, both live and pasteurized *A. muciniphila* (10^10^ CFU per day) were safe and tolerable for the study duration (3 months) with no reported adverse events. After 3 months, the group receiving pasteurized *A. muciniphila* showed improved insulin sensitivity as well as reduced cholesterol and body weight compared to the placebo. A reduction in the levels of markers for liver dysfunction was also seen in pasteurized *A. muciniphila* but not in the live microbe group. No change in the gut microbiome composition was seen in either of the groups. Strikingly, the pasteurization of *A. muciniphila* exacerbated its beneficial effects, raising interesting questions about the bacteria’s mechanism of action. Although an outer membrane protein called Amuc_1100 was found to partly recapitulate *A. muciniphila*’s beneficial effects,^231^ further elucidation of the mechanism is warranted.

As for obesity, the administration of probiotics was evaluated for IBD management. Wang et al. showed that administering a mixture of *L. plantarum* ZDY2013 from acid beans and *B. bifidum* WBIN03 from infant feces to dextran sodium sulfate (DSS)-induced UC in mice led to the downregulation of the pro-inflammatory cytokines and upregulation of antioxidant factors.^232^ Subsequently, the microbiome analysis of fecal samples from the mice revealed an increase in the abundance of unidentified Firmicutes and a decrease in Bacteroidetes. *L. fermentum* strains isolated from healthy individuals also demonstrated similar effects on the innate immune system in mice with DSS-induced colitis.^233^ The administration of *L. fermentum* KBL374 and KBL375 resulted in decreased levels of inflammatory cytokines and increased levels of the anti-inflammatory interleukin (IL)-10. *L. fermentum* administration also reshaped the gut microbiome of mice by increasing the abundance of *Lactobacillus* and *Akkermansia* spp. and decreasing Bacteroides numbers.^233^ Despite these reports of probiotics ameliorating IBD in mice, multiple clinical trial results have been discouraging. In a randomized, double-blind, placebo-controlled clinical trial comprising 142 patients with asymptomatic UC or CD, treatment with a multistrain probiotic cocktail showed no improvement in quality of life and other laboratory parameters, with a reduction in the fecal calprotectin levels of UC patients being the only significant finding.^234^ Another clinical trial comprising 56 UC patients showed that administering *B. longum* 536 resulted in a significant decrease in the disease activity score after 8 weeks, but it was not statistically different from the placebo group at the end of the treatment.^235^ Improvements in rectal bleeding and endoscopic score were observed in the probiotic group, but not in the placebo group. Moreover, the meta-analyses of clinical trials with UC and CD patients given probiotics concluded that probiotics are somewhat beneficial in UC, particularly in maintaining remission, but not in CD patients.^236,237^ This might be due to the insufficient period of treatment or delays in intervention.^237^

The bacterium *F. prausnitzii*, which belongs to the phylum Firmicutes, is a prominent member of the gut microbiome, accounting for 5% of total fecal bacteria.^238^ A negative association between the abundance of *F. prausnitzii* and IBD has been confirmed by a meta-analysis of 16 studies encompassing 1700 CD or UC patients.^239^ It was found that both CD and UC patients had a lower abundance of *F. prausnitzii* compared to healthy controls. Furthermore, patients with active CD and UC had reduced *F. prausnitzii* when compared to patients with CD and UC in remission, respectively. The importance of *F. prausnitzii* was further confirmed by in vivo studies in which the administration of bacteria to mice models of colitis led to reduced disease severity.^240,241^ Surprisingly, no clinical trial involving the supplementation of this bacteria to IBD patients has been conducted yet, likely due to *F. prausnitzii*’s extreme oxygen sensitivity, making it difficult to cultivate even in an anaerobic environment.^242^

Few studies have evaluated changes in the fungal diversity and virome of patients with IBD. Sokol et al. reported a decrease in the levels of *Saccharomyces cerevisiae* and an increase in *Candida albicans* in IBD patients compared to healthy subjects.^243^*Malassezia restricta*, a common skin fungus, was also found in abundance in the gut of CD patients.^244^ This fungus is known to elicit the release of inflammatory cytokines from innate immune cells, thus contributing to IBD development.^244^ Similarly, the gut microbiome of UC and CD patients had a significant expansion of *Caudovirales* bacteriophages, which may contribute to intestinal inflammation.^245^ Previous clinical trials have shown that *S. boulardii*, a closely related yeast to *S. cerevisiae*, can be used as an adjuvant therapy to induce remission or prevent the relapse of IBD in remission.^246^ However, no clinical trial has evaluated *S. boulardii* as a standalone IBD treatment.

Atopic dermatitis (AD) is a common skin allergic condition associated with a dysbiotic skin microbiome with a higher abundance of *S. aureus*. Studies on culturable Gram-negative bacteria from the skin of AD patients and healthy individuals revealed that *Roseomonas mucosa* from healthy volunteers, and not AD patients, was associated with the amelioration of AD in a mice model.^247^ On the basis of these results, a clinical trial comprising children below 7 years of age with AD (the most common group suffering from the disease) treated with *R. mucosa* isolated from healthy individuals was conducted.^248^ Treatment with the commensal bacteria led to an improvement in the skin epithelial barrier function, lowered *S. aureus* burden, increased the skin’s microbial diversity, and reduced the requirement of topical steroids for treatment. These positive effects were associated with the activation of the tissue-repair pathways by the glycerophospholipids produced by *R. mucosa* from healthy individuals, which were not produced by the isolates from AD patients.^248^

The alteration of vaginal flora has been shown to be associated with recurrent UTI, with the colonization of UTI-causing *E. coli* being derepressed due to the lower abundance of hydrogen peroxide-producing *Lactobacillus* in the vagina.^249^*L. crispatus* CTV-05 is a vaginal isolate that can produce hydrogen peroxide and adhere to the vaginal epithelial layer,^250^ thus making it an ideal probiotic candidate for the treatment of recurrent UTI. In a randomized, placebo-controlled Phase 2 trial comprising 100 women with recurrent UTI, the patients were given a placebo or *L. crispatus* CTV-05 intravaginally for 10 weeks.^251^ Recurrent UTI occurred in only 15% of the patients receiving the probiotic treatment compared to 27% in the placebo group. High levels of vaginal *L. crispatus* were observed in both groups, suggesting the expansion of the endogenous *L. crispatus* population in the placebo group. However, it was not associated with significant therapeutic advantages, unlike the *L. crispatus* CTV-05 isolate, which is able to outcompete *E. coli* in the vagina.^251^

##### 3.1.1.2 Prebiotic Supplementation

**Figure 1:**
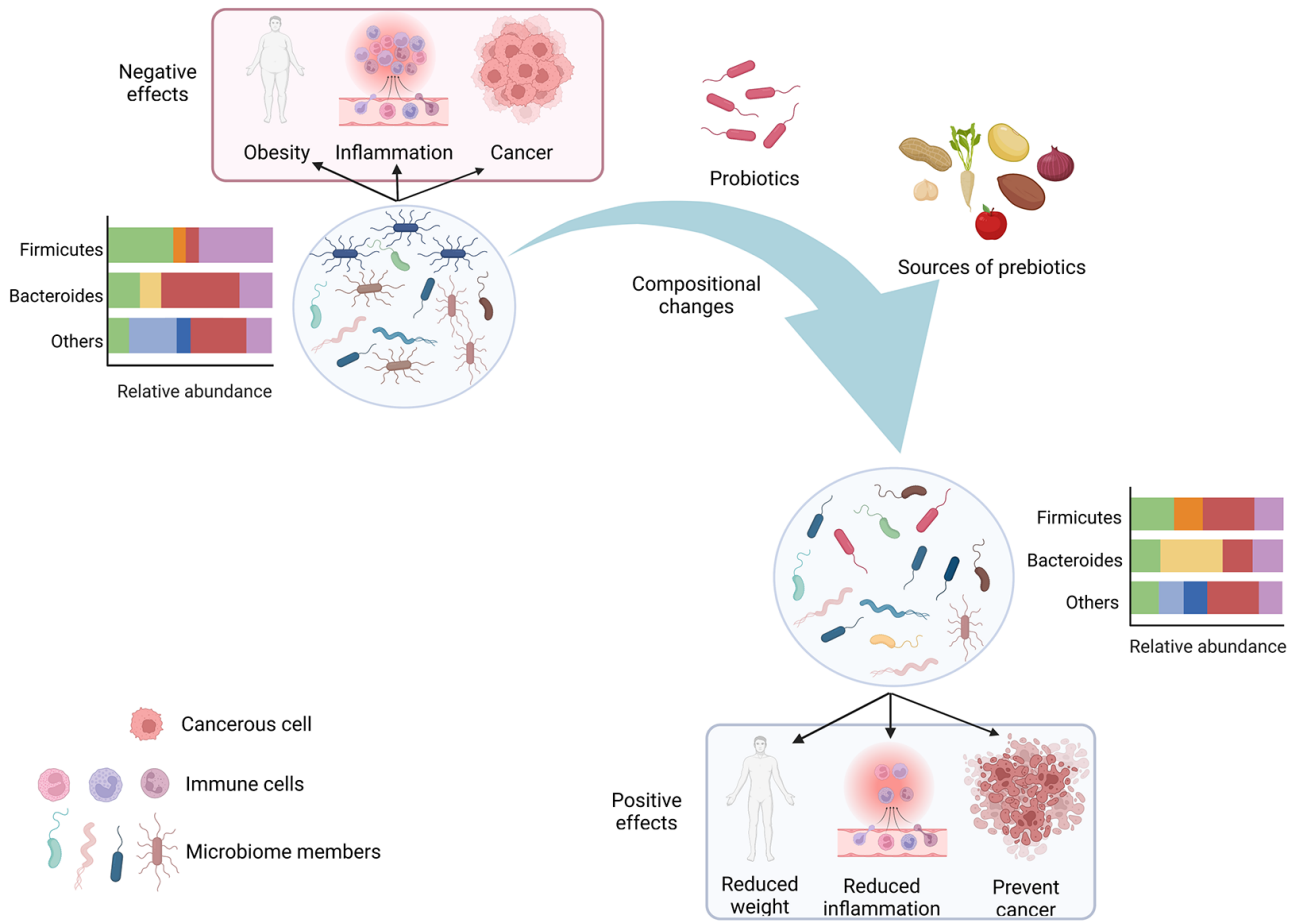
Modulation of microbiome composition by probiotics and prebiotics. A dysbiotic microbiome can contribute toward obesity development, inflammation, and cancer. Administering probiotics and prebiotics can change microbiome composition to ameliorate diseases.

An alternative to the exogenous supplementation of probiotics is the administration of prebiotics, which are nondigestible substrates that can be utilized by members of the host–microbiome to confer health benefits.^252^ Prebiotics are usually oligosaccharides that stimulate the growth of one or more species of bacteria already present in the microbiome. As different prebiotics can selectively increase the abundance of the microbes that can utilize them, these substrates can be used to remodel the microbiome—transitioning it from a disease state to a relatively healthier state (Figure 1). Some common prebiotics are fructo-oligosaccharides (FOS) derived from inulins, galacto-oligosaccharides (GOS), xylo-oligosaccharides (XOS), and lactulose.^253^ FOS, GOS, and XOS have been shown to promote *Bifidobacterium* expansion in the human gut, although there are conflicting reports of their effect on other bacterial genera due to differences in intervention doses and duration.^254−256^

In a prospective study on European newborn infants for the first 2 years of age, a gut microbiome analysis using stool samples was performed on infants with and without atopic dermatitis or other skin allergies.^257^ Lower counts of *Bifidobacterium* were observed in the first year of life in infants with allergies compared to healthy controls. A higher number of Clostridia at 3 months, higher *S. aureus* at 6 months, and lower *Bacteroides* at 12 months were also observed.^257^ An increase in *Bifidobacterium* levels after prebiotics administration to prevent atopic dermatitis was also evaluated in a randomized, prospective, placebo-controlled clinical trial comprising 259 infants receiving either 8 g/L of prebiotics (a mixture of GOS and FOS) or placebo.^258^ It was observed that the group that received prebiotics showed a lower incidence of atopic dermatitis at 6 months of age compared to the group that received the placebo. This was accompanied by an increase in the levels of bifidobacteria, but not lactobacilli, as determined by the colony-forming units from the stool samples. Other microbes in stool samples were not analyzed. A similar reduction in the incidence of allergies was reported in infants receiving prebiotics until 2 years of age, although changes in microbiome composition were not determined.^259^ The bifidogenic effect of FOS was also shown in adults with CD, wherein treatment with 15 g of FOS for 3 weeks led to an improvement in disease activity, an increase in the fecal bifidobacteria, and the modification of mucosal dendritic cell functions, such as increased IL-10 expression.^260^

The modulation of the gut microbiome by prebiotics also has applications in cancer therapy. In a study by Han et al., the administration of a colon-retentive inulin gel to a mice model of colorectal cancer improved the antitumor efficacy of the immune checkpoint blocker, antiprogrammed cell death protein-1 (α-PD-1).^261^ Previous studies have shown that patients that respond to immune checkpoint blockers have a higher abundance of beneficial bacteria, such as *Bifidobacterium*, *Akkermansia*, Ruminococcaceae, and *Faecalibacterium* in their gut microbiome compared to nonresponsive patients.^262−264^ Han et al. showed that the oral administration of the inulin gel to the mice led to the expansion of such beneficial bacteria, including *Akkermansia*, *Lactobacillus*, and *Roseburia*. This elicited a T cell response in the mice that worked in synergy with α-PD-1 for an enhanced antitumor effect.^261^

#### 3.1.2 Depletion of Specific Members of the Microbiome

Contrary to increasing the abundance of specific microbes in the microbiome, the selective depletion of certain members is another viable way of engineering the microbiome, particularly during infections. Since the discovery of penicillin in 1928, antibiotics have been the primary mode of defense against pathogens. However, most antibiotics currently in use are nonspecific in their antimicrobial activity and, consequently, cause a significant decrease in the diversity and richness of the human microbiome. Dethlefsen and Relman showed that there was a rapid shift in the composition and loss of diversity in the gut microbiome of individuals within 3–4 days after administration of ciprofloxacin.^265^ Although some members of the gut microbiome recovered after the end of the antibiotic course, recovery was incomplete, and the final composition was altered compared to the initial state. The altered composition of the microbiome due to antibiotics is also associated with increased susceptibility to other pathogens, immune dysregulation, and the rise of resistance genes.^266,267^ To negate the negative impact of antibiotics on the microbiome, targeted therapies against pathogens are required. Some small-molecule antibiotics are in clinical development and show promise against specific pathogens. For example, ridinilazole is a DNA-binding small molecule with highly targeted action against *Clostridium difficile*.^268^ In a Phase II randomized, double-blind clinical trial, ridinilazole was found to provide a superior clinical cure with no infection recurrence, compared to the current standard-of-care vancomycin.^269^ This was accompanied by a less disrupted microbiome in the case of ridinilazole. Vancomycin treatment led to a significant reduction of Firmicutes, Bacteroidetes, and Actinobacteria and the expansion of Proteobacteria, whereas ridinilazole showed only a modest reduction of the Firmicutes.^270^ Similarly, targeted antibiotics against the skin pathogen *S. aureus*, pneumonia-causing *P. aeruginosa*, and *Enterobacteriaceae* are also in development.^271^

In addition to the collateral damage to the microbiome, broad-spectrum antibiotics may also suffer from poor efficacy, such as in biofilms in which the pathogens remain impervious to the antibiotics. This is exemplified in the case of bacterial vaginosis, which is characterized by the displacement of beneficial *Lactobacillus* spp. with anaerobic bacteria, predominantly *Gardnerella vaginalis*, which forms a biofilm on the vaginal epithelium.^272^ Broad-spectrum antimicrobials have shown high short-term curing rates but are unable to prevent the recurrence of vaginosis, partly due to biofilm formation.^273^ Landlinger et al. developed a narrow-spectrum engineered endolysin, a peptidoglycan-degrading enzyme, by identifying and performing domain shuffling on 14 native endolysins present in *Gardnerella*.^274^ Among the various candidates, PM-447 was selected based on its high antimicrobial activity against various *Gardnerella* spp. and negligible activity against *Lactobacillus* spp. and other vaginal microbes. Interestingly, PM-447 was also able to target *Gardnerella* in vaginal samples from 13 patients with bacterial vaginosis and disperse the biofilm without affecting the remaining vaginal microbiome.^274^ Further evaluation of PM-447 in animal models is still awaited.

Vaccines also have the potential to eliminate pathogens in the human microbiome to prevent diseases. For example, the pneumococcal conjugate vaccine (PCV-7) against *Streptococcus pneumoniae* has significantly decreased the prevalence of invasive pneumococcal disease, particularly in young children.^275^ PCV-7 was designed against the seven virulent serotypes of *S. pneumoniae*, a natural colonizer of the upper respiratory tract. However, studies have shown that the niche vacated by virulent serotypes of the bacteria after vaccination was occupied by nonvaccine serotypes. Furthermore, an increase in the abundance of *S. aureus*, an ecological competitor of *S. pneumoniae*, was also observed.^276,277^ The use of a broader PCV-13 vaccine was found to increase the diversity and stability of the nasal microbiome,^278^ likely due to the opening of a larger niche that was occupied by nonpneumococcal bacteria.^279^

As an alternative to antibiotics and vaccines, bacteriophages have also been used as natural predators that target pathogenic bacteria and their associated diseases. The main advantage of using phages is their narrow host range, enabling precise elimination of the pathogen.^280^ Phages specific against *S. aureus*, *Enterococcus faecium*, *Vibrio parahaemolyticus*, *Acinetobacter baumannii*, and *Mycobacterium tuberculosis* have been previously isolated and found to be effective in targeting even antibiotic-resistant variants of the pathogens, at least in in vitro and in vivo models.^281^

Beyond infections, bacteriophages have also been used as therapies against other disorders by modulating the microbiome. Duan et al. showed that patients with alcoholic hepatitis have a higher number of fecal *E. faecalis* compared to nonalcoholic individuals or patients with other alcohol-use disorders.^282^ The authors identified cytolysin, an exotoxin produced by *E. faecalis* in the gut, to be responsible for liver injury. Upon administration of a bacteriophage targeting *E. faecalis* in mice inoculated with bacteria from patients with alcoholic hepatitis, a significant decrease in cytolysin levels was observed, accompanied by reduced liver injury. The fecal count of *Enterococcus* was also reduced, but no significant change in the microbiome composition was observed, indicating the targeted elimination of *E. faecalis*.^282^

In another study, Zheng et al. used a phage isolated from human saliva against the pro-tumoral *Fusobacterium nucleatum* to develop a novel therapy for colorectal cancer (CRC).^283^ Previous studies have shown that a high proliferation of *F. nucleatum* causes chemoresistance in CRC.^284^ Therefore, by using a phage targeting *F. nucleatum* in combination with nanoparticles loaded with a chemotherapy drug, the authors demonstrated superior efficacy of this method in different CRC mice models, compared to the chemotherapy drug alone or an antibiotic cocktail. A significant reduction of *F. nucleatum* and an increase in the abundance of antitumoral SCFA-producing bacteria were also seen in the gut of the mice.^283^

Bacteriophages have also been used to treat acne vulgaris, one of the most common dermatological disorders worldwide. One contributing factor to the development of the disease is the higher abundance of *Propionibacterium acnes*, although the exact mechanism is still debatable.^285^ To reduce the number of *P. acnes*, Brown et al. formulated an aqueous cream comprising a cocktail of bacteriophages isolated from human skin microflora against *P. acnes*.^286^ The cream was found to be effective in lysing *P. acnes* in vitro but has yet to be evaluated in animal models.

Engineered bacteria, both commensal and probiotic, are also emerging as robust therapies for targeted pathogen eradication. Although probiotic bacterial strains are known to prevent infection through the competitive exclusion of the pathogen and native production of antimicrobial agents,^287^ these strategies often show poor clinical efficacy. With the aid of synthetic biology, novel functionalities can be incorporated into the bacteria of choice to enable highly efficient and selective pathogen elimination. This is achievable due to the integration of biosensors in the bacteria that detect quorum signaling molecules produced by the target pathogen, such as acyl homoserine lactone (AHL) by *P. aeruginosa*(288) and autoinducing peptides (AIP) by *S. aureus*,^289^ which in turn activates the production and secretion of an antibacterial agent. Due to the high species specificity of quorum signaling molecules, the precise delivery of the therapeutic molecule becomes feasible—potentiating the use of engineered bacteria as viable alternative therapies for infections. Such strategies have been previously used to target *P. aeruginosa*,^288^*S. aureus*,^289^ and *V. cholerae*.^290^ Although these studies demonstrated the efficacy of the engineered bacteria in eliminating the target pathogen in either in vitro or in vivo models, the extent to which the microbiome is perturbed due to the administration of the engineered bacteria was not evaluated. Depending upon the chassis used, the engineered bacteria might rapidly transit through the microbiome or persist for a prolonged period. Although the former is unlikely to significantly alter the composition of the microbiome due to its inherent resistance to change,^291^ the latter might have a considerable impact, and future studies designed to evaluate this will have to be conducted.

In addition to the engineered bacteria, synthetic biology has also been used to engineer bacteriophages, repurposing them as antimicrobial delivery vehicles. Various studies have engineered bacteriophages to deliver CRISPR-Cas9 systems into their host.^292−294^ The CRISPR-Cas9 system is designed such that it recognizes a gene in the genome of the host, upon which it introduces double-stranded breaks in the genome, resulting in cell death. This approach is more specific compared to the use of wild-type bacteriophages because CRISPR-Cas9 is unable to induce cell death in the absence of the target gene. This strategy also enables the selective elimination of bacteria carrying antibiotic-resistance genes by programming CRISPR-Cas9 to identify these targets.^293^ However, the escape of target bacteria from the killing activity of CRISPR-Cas9 is a major drawback for this method, and escapees, either by chromosomal deletions or loss of CRISPR arrays, have been reported previously.^294^

**Figure 2:**
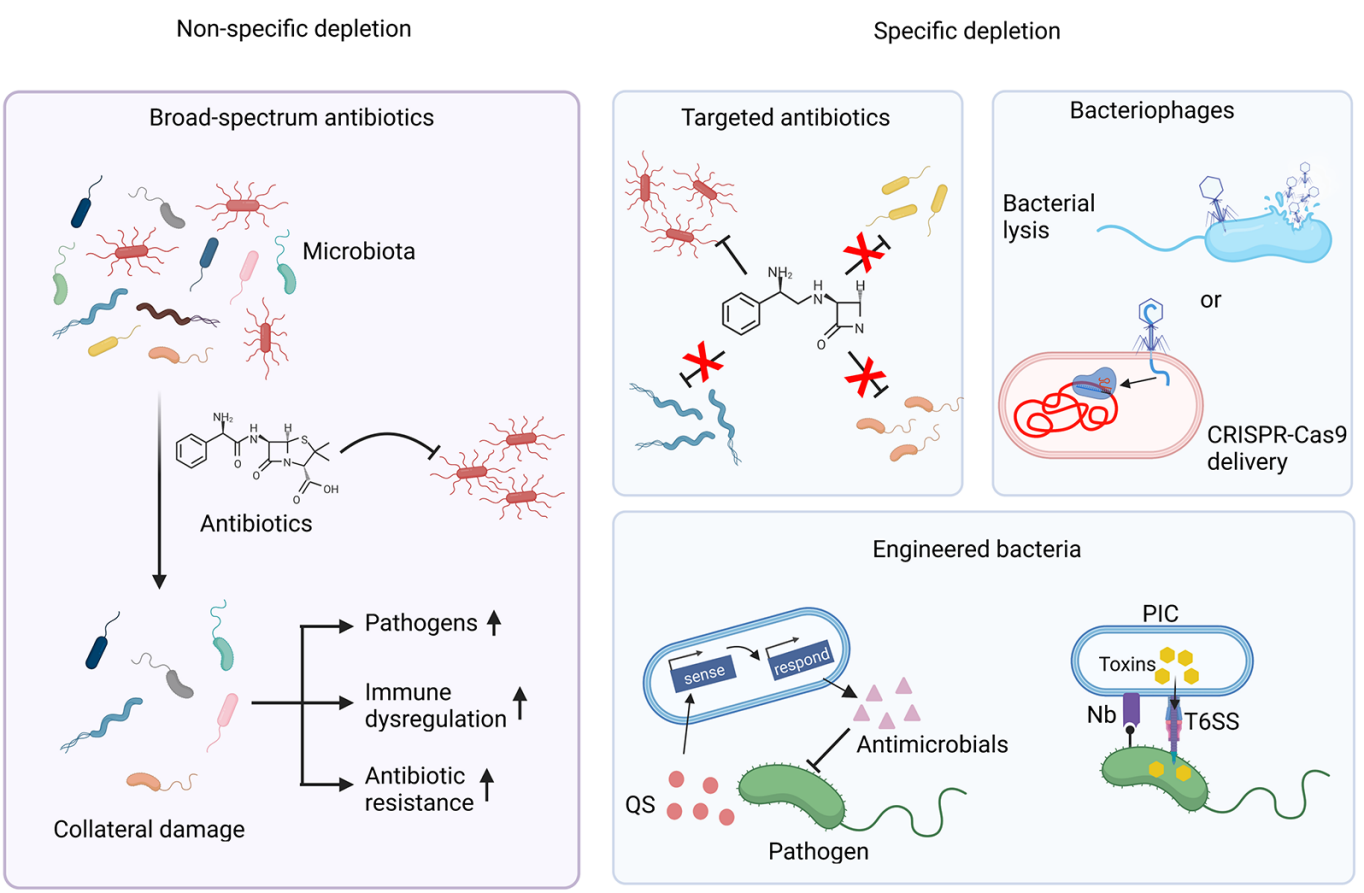
Strategies to deplete members of the microbiome. Nonspecific depletion can be mediated by broad-spectrum antibiotics. Specific depletion can be achieved by targeted antibiotics, bacteriophages, and engineered bacteria. QS, quorum signaling; PICs, programmed inhibitor cells; Nb, nanobody; T6SS, type-6 secretion system.

To improve upon these drawbacks, Ting et al. devised a novel strategy for targeted bacterial depletion by programmed inhibitor cells (PICs), which are engineered bacteria carrying the type-6 secretion system (T6SS) with a nanobody displayed on the surface (Figure 2).^295^ The nanobody recognizes an antigen on the surface of Gram-negative bacteria, such as outer-membrane proteins BamA and intimin, enabling cell–cell adhesion. T6SS is an antagonistic system through which the engineered bacteria can deliver antibacterial toxins into the target bacterial cell. The engineered bacteria protect themselves from the toxins by expressing immunity proteins. Due to the fluidic nature of the gut environment, T6SS alone is likely to be inefficient in delivering the toxins. Thus, the authors incorporated the nanobody into the bacteria for enhanced antibacterial activity.^295^ The authors showed that an intimin-expressing *E. coli* spiked into the fecal samples from mice was successfully depleted by 90% by using an engineered *Enterobacter cloacae* expressing an anti-intimin antibody on the surface with a native T6SS. This depletion was highly specific as an *E. coli* strain expressing partial intimin was unaltered. Analysis of the other bacteria in the fecal samples revealed only minor changes in microbiome composition, indicating the potential of PICs to be used as highly specific antimicrobials.

### 3.2 Changing the Functionality of the Microbiome

The previous section describes how microbiome composition is important in understanding its association with disease development and how it can be subsequently modulated for disease treatment. However, perhaps even more crucial than composition in understanding the influence that the microbiome exerts on its host is the microbiome’s function, corresponding to the active genes, proteins, and metabolites produced by its members. The significance of microbiome function stems from the likelihood that microbiomes with different compositions might still exhibit similar functions due to functional redundancy. This was observed in a study on the gut microbiome of obese and lean female twins that shared >93% of functional genes, comprising a core microbiome, despite having limited similarity at the phyla level.^296^ A similar relative abundance of microbial clades associated with clinically varied diseases also suggests that the function of the microbiome, and not its composition, has a stronger influence and association with human health.^297^

Morgan et al. demonstrated the importance of studying microbiome function in their study of microbiome samples from 231 IBD patients and healthy individuals, which were analyzed by 16S RNA sequencing and metagenomics.^298^ In addition to the expected changes in microbiome composition, there was a more consistent shift in microbial functions, such as decreased carbohydrate metabolism and amino acid biosynthesis, as well as increased nutrient transport and uptake. Similarly, the enrichment of lipopolysaccharide biosynthesis, pathogenic processes, and inflammatory pathways and the depletion of amino acids and energy metabolism have been reported in patients infected with HIV on antiretroviral therapy.^299^ To elucidate the exact mechanism by which microbiome function influences host health, multiomics studies beyond metagenomics are needed. These include transcriptomics, proteomics, and metabolomics to characterize the microbiome at the functional level in both healthy and diseased states. Although there are limited examples of such studies, some rationally designed interventions for modulating microbiome function have been reported before and are discussed later.

#### 3.2.1 DNA Conjugation-Mediated Engineering

DNA conjugation is a method of in situ engineering of the microbiome by delivering a genetic payload to the target microbe via a donor bacterium. These payloads can be mobile genetic elements carrying genes to incorporate novel functionalities into the target bacteria. Such an engineering approach is advantageous because this enables the modification of even unculturable bacteria with complex phenotypes that may not be adequately replicated in non-native bacterial strains.^300^ In a study by Brophy et al., the authors used a *B. subtilis* strain to transfer a miniaturized integrative and conjugative element (mini-ICE*Bs1*) efficiently to various Gram-positive bacteria isolated from the human skin and gut microbiome.^301^ The conjugative transfer was placed under the control of an inducible promoter, and the genes that enable further propagation of the ICE*Bs1* beyond the initial recipient were deleted as a safety feature. Because new donor strains carrying the DNA to be transferred can be created with ease, this strategy will be useful in rapidly engineering microbiomes with synthetic programs and modulating their function.

Another DNA conjugation strategy was developed by Ronda et al. wherein an *E. coli* donor strain was engineered to transfer mobile plasmids, either replicative or integrative, into recipient Gram-positive and Gram-negative strains.^302^ The authors demonstrated that this method can be applied to engineer the microbiome by using the donor *E. coli* to deliver green fluorescent protein (GFP) into members of the gut microbiome in mice. By using a library of mobile plasmids, up to 5% of the bacteria were found to receive the plasmid 6 h postadministration of *E. coli.* The recipients belonged to all four major phyla in the gut microbiome, namely, Firmicutes, Bacteroides, Actinobacteria, and Proteobacteria.^302^ Interestingly, the transconjugants persisted only for 72 h postadministration of the *E. coli* donor, suggesting unstable plasmid maintenance.

Jin et al. developed a genetic manipulation pipeline through which they were able to genetically modify 27 of the nonmodal bacteria belonging to the Firmicutes/Clostridia class.^303^ The authors identified the culture conditions that can support bacterial growth followed by a library of genetic tools, including the origin of replications and antibiotic-resistance markers with potent promoters that are functional in the target bacteria. By utilizing these tools and further optimizing the conjugation protocol, the successful delivery of the deactivated Cpf1-based CRISPRi system for regulating gene expression was achieved. In this study, the authors demonstrated the application of their pipeline to modulate metabolites produced by the gut microbiome, such as the bile acid pool in mice, which has numerous implications on host health.^303^

The studies described earlier indicate that DNA conjugation is a viable method for modulating microbiome function, although the area is still in infancy and needs further development, particularly an assessment of safety in humans. This is pertinent in the case of mobile genetic elements that are used to deliver the payloads, as such elements have a high tendency to propagate further into nontarget members of the microbiome by horizontal gene transfer.^304^ In addition, limited studies on nonmodal members of the microbiome remain a bottleneck in their genetic engineering because it is difficult to reliably predict the functionality of the payload introduced into these microorganisms.

#### 3.2.2 Use of Enzyme Inhibitors

The metabolic activity of microbial enzymes is an important process through which the microbiome can influence host health. Apart from playing a role in the normal functioning of the microbe itself, these enzymes can also metabolize drugs, prodrugs, and xenobiotics administered to the host, leading to unintended and potentially adverse outcomes.^305^ This can be mitigated by using inhibitory chemicals that act on the specific microbial enzyme. For example, SN-38 is an anticancer drug used in colon cancer and against lung and brain tumors formed from the intravenously administered prodrug, CPT-11, in the liver. Subsequently, it is glucuronidated by UDP-glucuronosyltransferase in the liver into SN-38G, which is excreted into the GI tract.^306^ Here, it is again converted to SN-38 by the bacterial β-glucuronidase enzymes—causing diarrhea and prohibiting an escalation in chemotherapy drug dosage. Although eliminating gut bacteria by antibiotics can potentially prevent SN-38 toxicity, this method has several drawbacks, as discussed in section 3.1.2. Instead, Wallace et al. employed high-throughput screening to identify potent inhibitors of the bacterial β-glucuronidase that do not target orthologous mammalian enzymes.^307^ In addition, these inhibitors neither killed the bacteria nor harmed mammalian cells. In a mice model, the inhibitor was found to protect mice from the chemotherapy drug’s toxicity, although improvements in drug efficacy remain to be studied.

Inhibitors for the gut microbiome-dependent production of trimethylamine (TMA) *N*-oxide (TMAO), which is associated with cardiovascular risks in humans, have also been reported.^308,309^ Dietary choline, phosphatidylcholine, and carnitine are converted into TMA by the microbial choline-TMA lyase enzymes, which is subsequently converted to TMAO by hepatic flavin monooxygenase.^310,311^ Wang et al. identified an analogue of choline, 3,3-dimethyl-1-butanol (DMB), that was observed to inhibit multiple microbial TMA lyases without killing the microbes.^308^ The treatment of mice fed a high choline or l-carnitine diet with DMB resulted in lower plasma TMAO levels, the attenuation of atherosclerotic lesions, and the formation of macrophage foam cells. The same group also developed two other TMA lyase inhibitors, fluoromethylcholine and iodomethylcholine, that are nonlethal and able to accumulate within the microbe, resulting in a sustained decrease in TMAO levels in mice for 3 days after a single oral dose.^309^ Treatment with these inhibitors also prevented thrombus formation by reducing platelet adherence to the collagen matrix in the arteries in mice fed choline. Interestingly, the inhibitors caused a change in gut microbiome composition despite being nonlethal, suggesting additional selection pressure on the microbes due to the inhibitors, which might eventually lead to resistance development.

The development of enzymatic inhibitors to modulate microbiome function faces two major challenges. First, the inhibitor should be able to act on all related microbial enzymes. In the absence of broad-spectrum inhibition, noninhibited microbial species may compensate, resulting in no net change in microbiome function. Second, the presence of human enzymes with similar functions as the microbial enzyme will necessitate the screening of a large library of chemicals to find targets that are inhibitory toward microbes and not mammalian cells, which might not be achievable in some cases.

#### 3.2.3 Microbiome Metabolite Modulation by Engineered Microorganisms

Among the various strategies to modulate microbiome function, the most advanced is the administration of exogenous engineered bacteria that are either commensal or probiotic. Contrary to the administration of wild-type bacteria that are primarily used to change microbiome composition, engineered bacteria are equipped with novel functionalities that enable the regulation of microbiome function. By using the large repertoire of synthetic biology tools, bacteria can be reprogrammed to specifically target a disease, resulting in a highly precise autonomous therapy. Such therapies have been developed to target metabolic diseases,^312−314^ prevent cancer,^315−317^ inhibit pathogens,^318,319^ and address other conditions^320−322^ (for recent reviews, see refs (323−326)). An increasing number of such studies have been reported thus far. However, in this section, we will only present studies that show how the microbiome has been implicated to play a major role in disease development through its metabolites, thus necessitating its functional modulation for therapy.

There is an intense interplay of metabolites between the human microbiome and its host, with microbiome metabolic pathways significantly associated with 34% of blood and 95% of fecal metabolites.^327^ One such metabolite is ammonia, which is primarily produced by the metabolism of amino acids in our food by gut microbes. The human body regulates the levels of ammonia via the liver’s urea cycle.^328^ However, in the case of liver failure, the metabolite accumulates in the blood, which can act as a neurotoxin at high concentrations.

**Figure 3:**
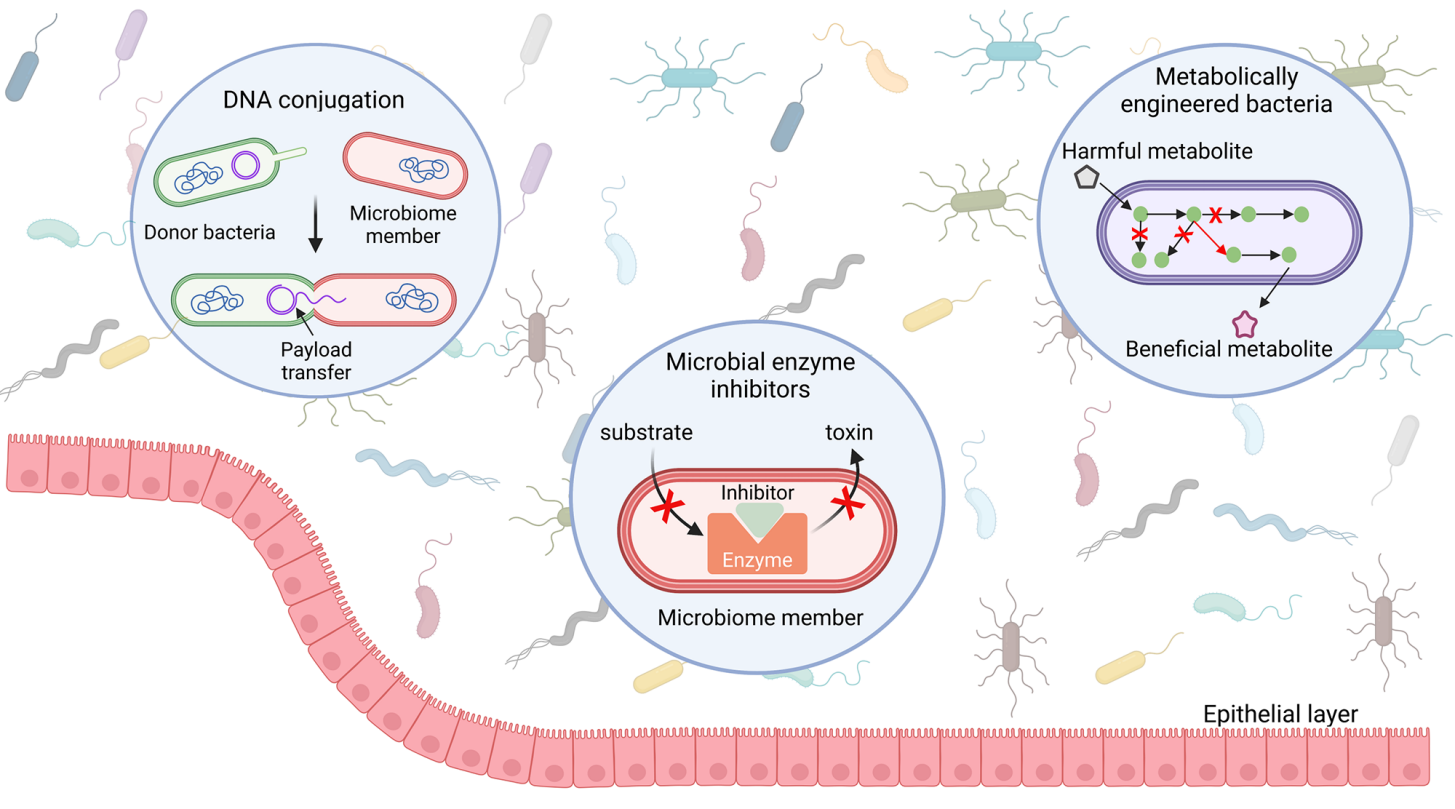
Modulation of microbiome functionality by DNA conjugation, enzyme inhibitors, and engineered bacteria.

To develop a therapy that reduces ammonia levels, *E. coli* Nissle 1917 (EcN) was genetically modified to convert gut ammonia into l-arginine, boosting the urea cycle.^329^ The metabolic pathway was placed under the control of the anaerobic-inducible *fnrS* promoter (PfnrS) such that metabolic conversion was initiated only in the gut’s low-oxygen environment. In addition, the bacterial strain was made auxotrophic for thymidine as a biocontainment strategy. This strain was found to reduce ammonia levels in mice given a high-protein diet or administered with thioacetamide, a liver toxin. The engineered bacterium was also found to be safe to use in healthy volunteers at a dose of 5 × 10^11^ CFU three times a day for 14 days.^329^ However, in a Phase 2 clinical trial, the engineered EcN failed to significantly reduce blood ammonia in patients with cirrhosis compared to placebo.^330^ The engineered EcN has since been repurposed for anticancer therapy in conjunction with immune checkpoint inhibitors by metabolizing waste ammonia from tumors into l-arginine, increasing T cell response against cancer (Figure 3).^316^

SCFAs produced by the fermentation of dietary fibers by the gut microbiome have anti-inflammatory activity and play an important role in various host processes, such as improving intestinal barrier integrity and reducing colon cancer risk.^331^ SCFAs, particularly butyrate, are also able to regulate appetite, decrease insulin resistance, promote fat oxidation, and stimulate the release of insulinotropic hormones.^332,333^ Due to these effects on host metabolic activity, butyrate is likely to have a therapeutic effect on patients with obesity and diabetes. Oral delivery of butyrate is challenging due to its poor bioavailability and disagreeable odor and taste. Thus, different bacteria have been metabolically engineered to produce butyrate in the gut as an alternative treatment for obesity and diabetes. *Bacillus subtilis* SCK6 naturally produces butyrate at very low levels and thus was engineered to produce butyrate via the butyryl CoA:acetic acid CoA transferase pathway that is present in the gut’s butyrogenic microbes.^334^ Further deletion of competing metabolic pathways yielded a strain that can produce 1.5 g/L of butyrate in vitro. When evaluated in mice given a high-fat diet, the engineered bacteria were able to retard weight gain, reduce visceral fat accumulation, and improve glucose tolerance. In addition to the decrease of Firmicutes and increase in Bacteroidetes, analysis of the metabolic pathways of the gut microbiome revealed a significant enhancement of the genes involved in carbohydrate, amino acid, vitamins, and energy metabolism.^334^ EcN has also been engineered to produce 0.5–1 g/L butyrate in vitro but has yet to be evaluated in in vivo models.^335,336^

Extracellular adenosine triphosphate (eATP) is an IBD-associated metabolite produced by activated immune cells and the gut microbiome. eATP is known to promote pro-inflammatory cytokine production and neuron apoptosis, as well as to suppress anti-inflammatory responses. A probiotic yeast, *S. cerevisiae*, was genetically modified to sense eATP in the gut and respond by producing ATP-degrading enzymes that convert eATP to AMP. In turn, AMP is further broken down to immunosuppressive adenosine.^337^ To enable *S. cerevisiae* to respond to eATP, a human P2Y2 receptor evolved to sense eATP with 1 000-fold higher sensitivity was integrated into the probiotic strain. This was combined with a potent ATPase from potato to create an IBD treatment. In mice models of colitis, the engineered probiotic ameliorated inflammation, as observed by the lower expression of pro-inflammatory cytokines, reduced colon shortening, and improved histological scores.^337^ The engineered probiotic performed better than the probiotic with constitutively expressed ATPase and even standard-of-care IBD therapies, suggesting the therapy’s potency against inflammatory disorders.

### 3.3 Natural and Synthetic Microbial Consortia

Microbial communities, such as those in the human microbiome, perform complex functions shaped by dynamic interactions within the community and with the environment. Such intricate functions are unlikely to be recapitulated by individual populations, which has resulted in heightened interest in microbial consortia. Additionally, increased microbial diversity within the consortia might also impart resilience against environmental changes, such as nutrient limitation.^338^ Currently, two types of microbial consortia are being used for therapeutic applications: naturally occurring with undefined composition, such as the gut microbiome, which can be used as a fecal microbiota transplant (FMT); and synthetic, comprising a predetermined cocktail of probiotics or commensal microbes. Here, we will discuss some examples of these microbial consortia being used to target a variety of diseases and the challenges faced by these strategies.

#### 3.3.1 Fecal Microbiota Transplantation

**Figure 4:**
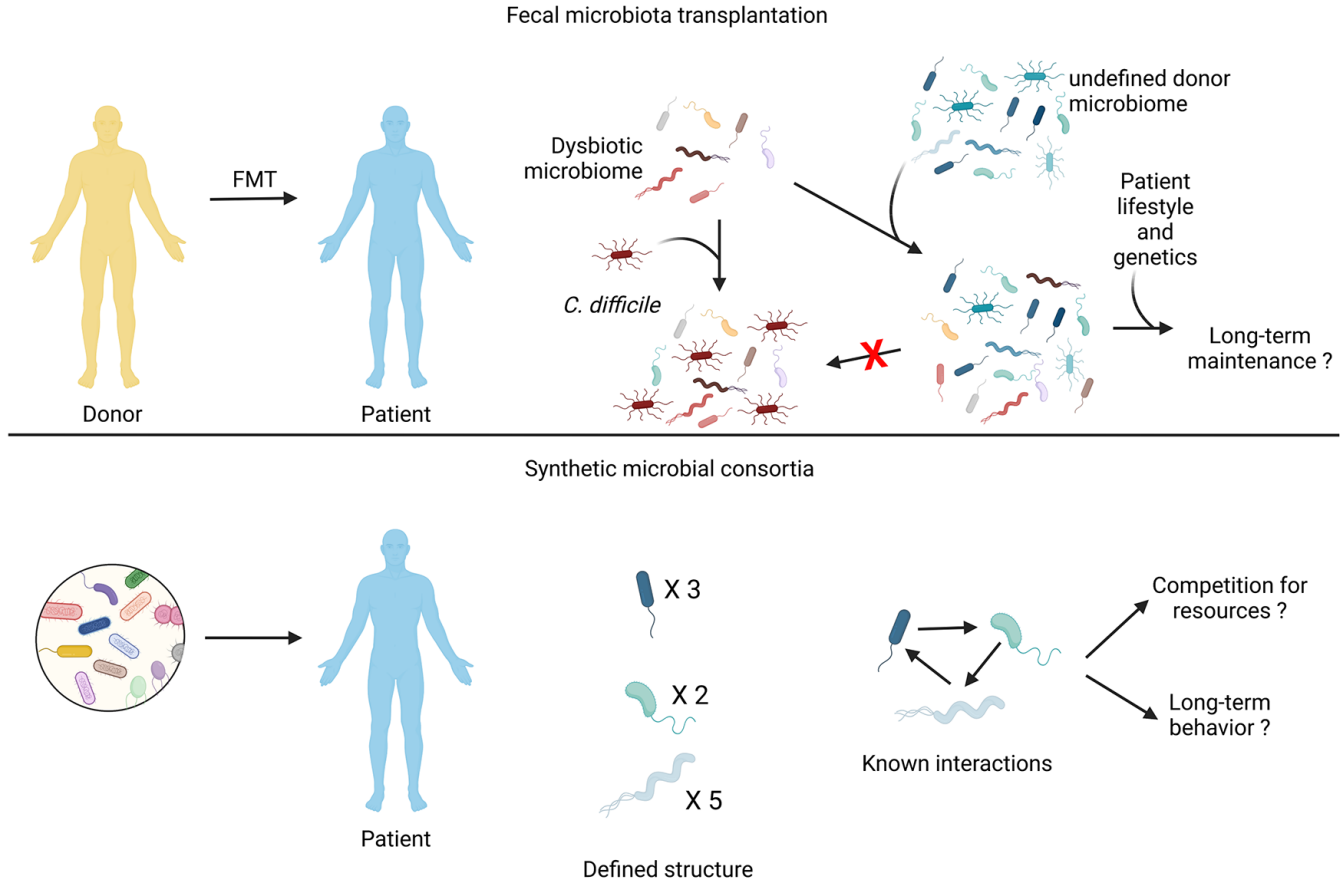
Engineering the microbiome by fecal microbiota transplantation (FMT) and synthetic microbial consortia. The main features of both strategies are presented along with unknown parameters (marked by “?”).

FMT involves the administration of dried feces, either the patient’s own or from a healthy donor to the patient (Figure 4). The route of administration might vary, ranging from oral consumption of freeze-dried capsules and small/large intestine infusion to enema.^339^ The aim is to reconstitute the native disease-associated microbiome into a healthier version to provide a positive health impact. FMT has shown the most promise in recurrent *C. difficile* infection with a resolution rate of 85–90% and is highly recommended for both mild and severe cases.^340^ After initial vancomycin treatment for patients with *C. difficile* in a randomized clinical trial, FMT showed higher efficacy than a continued vancomycin regimen, accompanied by an increase in Bacteroidetes and *Clostridium* clusters and a decrease in Proteobacteria.^341^ In mice and humans given broad-spectrum antibiotics, autologous FMT enabled the rapid and complete recovery of the dysbiotic microbiome within days and was the most effective intervention.^342^ Surprisingly, the administration of a cocktail of probiotics significantly delayed the recovery of the microbiome compared to spontaneous recovery, with the recovery being incomplete. This was attributed to the soluble factors produced by the probiotics that caused the inhibition of the indigenous microbiome.^342^

Apart from *C. difficile* infection, FMT has been used to treat ulcerative colitis,^343,344^ type 2 diabetes,^345^ irritable bowel syndrome,^346^ and cancer;^347^ however, only limited case studies and clinical trials have been reported to date. Further well-designed clinical trials are required to confirm FMT’s efficacy for indications beyond *C. difficile* infection.

There are certain aspects of FMT that must be considered and studied before it can be broadly applied in clinical settings. The selection of an appropriate donor is one of the key components for FMT success. Although autologous FMT is likely to provide superior compatibility, it may not always be feasible, thus necessitating a stool donor. Meticulous screening of the donor’s gut microbiome for potentially pathogenic species is required prior to FMT to ensure procedural safety. This has especially become relevant in the COVID-19 pandemic due to the detection of the SARS-CoV-2 virus in individual fecal samples.^348^ Additionally, studies have been undertaken to identify the donors whose stool samples are most likely to result in successful engraftment of the gut microbiome. A high taxonomic diversity and the presence of specific bacterial families in the donor stool have been proposed to be key to disease-specific restoration of gut homeostasis in recipients.^349^ Beyond donor selection, the recipients’ genetics, diet, and lifestyle are also likely to play a role in FMT maintenance.^339^

As an alternative to FMT, Seres Therapeutics developed SER-109, a natural consortium of bacterial spores from healthy donors to treat recurrent *C. difficile* infection.^350^ The spores are primarily from bacteria belonging to Firmicutes, as other phyla are not spore-formers. This is advantageous as previous studies have shown Firmicutes to be associated with a relatively higher concentration of secondary bile acids, which inhibits vegetative growth of *C. difficile*.^350^ Moreover, these bile acids also prevent *C. difficile* spore germination, which is a major cause of recurrent infection.^351^ The use of a purified spore consortium allows for oral delivery because the spores are resistant to gastric acid, reducing the risk of pathogen transmission seen in FMT. In a randomized, double-blind, placebo-controlled Phase 3 clinical trial comprising 182 patients, the group of patients receiving oral SER-109 after standard antibiotic regime showed a reduced recurrence of *C. difficile* infection compared to the patients receiving placebo after antibiotics (12% vs 40%).^352^ Patients receiving SER-109 showed an engraftment of the dosed species within 1 week, which persisted for the study duration (8 weeks) with an increased abundance of Firmicutes and decreased proinflammatory Enterobacteriaceae. A higher concentration of secondary bile acids in the fecal samples from patients given SER-109 was also observed.^352^

#### 3.3.2 Synthetic Microbial Consortia

Due to the undefined community structure in naturally occurring microbiomes, it is challenging to quantify the contributions of individual members toward a specific function, making it unsuitable for further optimization. Synthetic microbial consortia can fulfill this unmet need as they can be rationally designed with reduced microbial complexity to perform functions that can predictably impart a therapeutic benefit to the host. For example, Tanoue et al. identified a consortium of 11 bacterial strains isolated from the feces of healthy human donors that can robustly induce interferon-γ-producing CD8^+^ T cells in the intestine.^353^ The 11 strains are low-abundance members of the microbiome including those belonging to *Bacteroides* spp. and *Parabacteroides* spp., *Eubacterium limosum*, *Ruminococcaceae bacterium cv2*, *Phascolarctobacterium faecium*, and *Fusobacterium ulcerans*.^354^ Upon colonization in mice, the microbial consortium provided resistance to infection by *Listeria monocytogenes* and enhanced the efficacy of immune checkpoint inhibitors in tumor models.^353^

GUT-108, a rationally designed consortium of 11 bacterial strains, was also shown to reverse colitis in a mice model.^355^ The consortium comprises strains that can perform functions that are reduced in the gut microbiome of IBD patients. These include the production of SCFAs such as butyrate and propionate, secondary bile acids, deoxycholic acid, and lithocholic acid, as well as indole and its derivatives.^356,357^ Additionally, several strains in GUT-108 produce antimicrobial factors that prevent the growth of opportunistic pathogens, which may further exacerbate the inflammatory response.^355^ The administration of GUT-108 to a mice model of experimental colitis prevented the expansion of pathogenic bacteria belonging to the Enterobacteriaceae family, which was observed in mice receiving phosphate buffered saline (PBS). The successful engraftment of 10 strains of GUT-108 was observed in mice, which led to reduced inflammatory cytokines, induced IL-10 production, and increased the levels of metabolites that promote mucosal healing and immunoregulatory responses.^355^

Beyond the synthetic consortia comprising wild-type bacterial strains, engineered bacteria with defined characteristics can also be used to develop synthetic communities. For example, Kong et al. created six two-strain consortia with unique interactions ranging from commensalism to predation.^358^ They used these strains to further develop three- and four-strain member communities with predictable behavior, demonstrating the successful engineering of social interactions in a bacterial community. This approach may enable the design of synthetic consortia with more complex behavior that can impart beneficial effects to the host.

The design of microbial consortia presents unique challenges compared to single microbial populations. Similar to natural microbiomes, synthetic consortia should be able to maintain homeostasis and prevent certain members from outcompeting others even in different nutritional environments. However, this is difficult to achieve because microbes often show varied abilities to metabolize different resources, making the long-term prediction of homeostasis unfeasible. Applying synthetic biology to incorporate genetic circuits, such as the oscillatory predator–prey system,^359^ into the microbes may potentially mitigate competition between consortium members. Another challenge is predicting the behavior of the microbial consortia, which can be attributed to the lack of omics-level understanding of metabolic interactions between the microbes. The knowledge gained through multiomics studies combined with computational modeling will ultimately aid in our ability to design synthetic microbial consortia with predictable and controllable functions.^360^

### 3.4 Current Challenges and Limitations of Microbiome Engineering

The examples presented earlier (summarized in Table 2) demonstrate that it is feasible to engineer the microbiome for various therapeutic outcomes in the host. So far, in vitro and in vivo evaluations of different methods to modulate microbiome composition and function have shown promising results. With the exception of FMT, most therapeutics are still awaiting successful clinical translation. There are unique challenges to the development of different therapeutics based on a rationally engineered microbiome, which have been alluded to earlier. However, to further accelerate microbiome engineering to develop safe and effective therapeutic products, there is a need to bridge the knowledge gaps pertaining to microbe–microbe and microbe–host interactions and build novel tools to broaden the scope of microbiome engineering, as discussed later.

**Table 2:** Summary of Strategies for Engineering the Microbiome To Target Different Diseases

strategy	indication	therapeutic microbes/molecules	features	ref
probiotics	obesity and diabetes	*A. muciniphila*	the bacterium is depleted in patients with obesity, type 2 diabetes, and hypertension	(230)
			obese patients who had received the probiotic for 3 months showed improved insulin sensitivity and reduced cholesterol	
			pasteurized bacteria showed higher efficacy compared to live bacteria	
	IBD	*F. prausnitzii*	IBD patients exhibit reduced abundance of *F. prausnitzii*	(240, 241)
			administration of the bacteria led to reduced disease severity in a mice model of colitis	
			no clinical trial conducted yet, likely due to difficult cultivation of the bacteria	
		*S. boulardii*	the probiotic can be used as an adjuvant to induce remission or prevent relapse of IBD	(246)
			clinical trials using the probiotics alone have not yet been conducted	
	atopic dermatitis	*R. mucosa*	the bacteria isolated from healthy individuals	(248)
			treatment with the commensal bacteria led to an improved skin barrier and reduced *S. aureus* burden	
			the therapeutic effect was limited to the bacterial isolate from healthy volunteers and not the patients	
	urinary tract infection	*L. crispatus* CTV-05	*L. crispatus* CTV-05 is a vaginal isolate that adheres to the vaginal epithelial layer and suppresses the growth of pathogenic *E. coli*	(251)
			in a Phase 2 clinical trial, probiotic administration led to a significant reduction in the recurrent UTI incidence	
prebiotics	atopic dermatitis	mixture of GOS and FOS	GOS and FOS can promote *Bifidobacterium* growth	(258)
			patients receiving GOS and FOS showed lower incidence of atopic dermatitis with expansion of Bifidobacteria	
	Crohn’s disease	FOS	patients receiving FOS showed an improvement in disease and increased fecal Bifidobacteria	(260)
	colorectal cancer	inulin gel	developed colon-retentive inulin gel that increased the abundance of beneficial bacteria such as *Bifidobacteria* and *Akkermansia*	(261)
			expansion of the beneficial bacteria led to the increased antitumor efficacy of immune checkpoint blockers	
targeted antibiotics	*C. difficile* infection	ridinilazole	ridinilazole is a small DNA-binding molecule with highly specific action against *C. difficile*	(269)
			it was found to be superior to standard-of-care vancomycin in a Phase 2 clinical trial	
	*Gardnerella* infection	engineered endolysin PM-447	bacterial vaginosis caused due to reduced *Lactobacillus* spp. and increased *Gardnerella* spp.	(274)
			engineered endolysin formed by domain shuffling of native enzymes in *Gardnerella*	
			PM-447 has negligible activity against *Lactobacillus* but can target *Gardnerella*, including dispersing the biofilm	
bacteriophages	alcoholic hepatitis	bacteriophage against *E. faecalis*	cytolysin, produced by *E. faecalis*, is responsible for liver injury in alcoholic hepatitis	(282)
			targeting the bacteria with the phage led to a reduction in liver injury and no significant perturbation of gut microbiome composition	
	colorectal cancer	phage against *F. nucleatum*	phage isolated from human saliva	(283)
			*F. nucleatum* causes chemoresistance in colorectal cancer	
			by targeting the bacteria with the phage in conjunction with a chemotherapy drug, superior efficacy was observed in mice model	
	acne vulgaris	bacteriophages against *P. acnes*	an aqueous cream formulated comprising bacteriophages against *P. acnes* isolated from human skin flora	(286)
			validation only in in vitro model	
enzyme inhibitors	colon cancer	inhibition of bacterial *β-glucuronidase*	bacterial *β-glucuronidase* converts the harmless byproduct of the anticancer drug into toxic SN-38	(307)
			developed enzyme inhibitors neither killed the bacteria nor harmed the mammalian cells	
	cardiovascular diseases	inhibition of TMA lyases	microbial TMA lyases involved in the synthesis of TMAO, which is associated with cardiovascular diseases	(308, 309)
			developed inhibitors of TMA lyases are nonlethal to microbes and are able to sustain TMAO decrease in mice models	
engineered microbes	pathogen elimination	commensal and probiotic bacteria	the bacteria is engineered to sense the quorum signaling molecules produced by the pathogen	(288, 289)
			in response, the engineered bacteria secretes the antibacterial agent	
	hyperammonemia	engineered EcN	EcN was engineered to convert ammonia into l-arginine to boost the urea cycle	(329)
			biocontainment strategy incorporated into the bacteria for safety	
			although effective in various mice models, the bacteria failed to show significant efficacy in Phase 2 clinical trial	
	diabetes and obesity	engineered *B. subtilis*	the bacteria were metabolically rewired to produce 1.5 g/L butyrate in vitro	(334)
			in an obesity mice model, the engineered bacteria were able to retard weight gain and fat accumulation	
	IBD	engineered *S. cerevisiae*	*S. cerevisiae* genetically engineered to sense eATP by using a human P2Y2 receptor	(337)
			in response to eATP, the engineered yeast produces ATP-degrading enzymes	
			the engineered microbe performed better than the standard-of-care IBD therapies in a colitis mice model	
natural microbial consortia	recurrent *C. difficile* infection	fecal microbiota transplantation	fecal sample from healthy donor administered to patients	(341)
			risk of pathogen transmission	
			genetics and lifestyle of the recipient may affect efficacy	
		SER-109, bacterial spores from healthy donors	oral delivery possible	(352)
			reduced risk of pathogen transmission	
			clinical efficacy observed in Phase 3 trial	
synthetic microbial consortia	*L. monocytogenes* infection	11 bacterial strains from healthy donors	the consortium can induce CD8^+^ T cells in the intestine	(353)
	cancer		the bacterial strains are low-abundance members of the microbiome	
			the consortium can enhance the efficacy of immune checkpoint inhibitors in tumor models	
	colitis	GUT-108, consortium of 11 bacterial strains	the consortium can perform functions reduced in IBD patients	(355)
			production of antimicrobial factors to prevent pathogen growth	
			induction of anti-inflammatory molecules	

#### 3.4.1 Inadequate Use of Multiomics Studies

As part of the Human Microbiome Project (HMP), different microbial communities in the human body have been comprehensively characterized by 16S rRNA gene sequencing to decipher the microbiome’s taxonomic complexity. Meanwhile, metagenomic whole-genome shotgun sequencing provides insight into the pathways present in the microbiome and their functions.^361^ However, these data sets do not shed light on intramicrobial community interactions, how the microbiome interacts with the host, and how the host responds to its resident microbiome. A thorough understanding of these interactions is necessary for determining the causal role that the microbiome plays in disease development, which will eventually lead to novel therapies through microbiome engineering. To achieve this, healthy and diseased cohorts will have to be subjected to various multiomics assays encompassing not only the microbiome but also the host. Such studies are ongoing for preterm birth, IBD, and type 2 diabetes as part of the second phase of the HMP.^362^ In the case of IBD, stool samples will be collected from patients with IBD and healthy controls. These will be used for multiomics assays such as 16S rRNA gene sequencing, metagenomic and metatranscriptomic sequencing, protein profiling, and metabolomics. In addition, the corresponding changes in the host will be determined by performing RNA-seq on colon biopsy samples, studying DNA methylation of the host genome, and interrogating the host cells with different metabolites. Importantly, the study will also include a survey of yeast and viruses found in the microbiome.^362^

#### 3.4.2 Spatiotemporal Control of the Engineered Microbiome

For the engineered microbiome to safely impart health benefits to the host, it should exhibit predictable spatiotemporal behavior. Environmental perturbations and spatial organization are major variables that can influence the complex and dynamic interactions of the microbiome.^363,364^ For example, Sheth et al. studied the microbial biogeography of the mouse intestine and showed both positive and negative associations between individual taxa.^365^ Additionally, the authors showed that mice given a low-fat or high-fat diet led to changes in the species richness and altered spatial organization of the colon microbiome. Therefore, the engineered microbiome should be resilient to any perturbations and be able to adapt to its community structure over different time scales to continue providing the intended therapeutic effect to the host. This is particularly important in the case of engineered microbiomes with an associated fitness cost, which might lead to evolutionary adaptations through random mutations and horizontal gene transfer. Similar adaptations have been observed in the commensal microbe *Bacteroides fragilis*, leading to its long-term prevalence in the human gut.^366^ Moreover, mechanisms to safeguard the host against unintended microbiome functions should be built-in as a safety feature.

A potential way to achieve this level of dynamic control of the composition and function of the microbiome is through synthetic biology. Genetic functionalities, such as biosensors, can be incorporated into the microbiomes such that they can be controlled through external stimuli. Additionally, genetic circuits can be designed for autonomous feedback control of the microbiome.

#### 3.4.3 Genetically Intractable Microorganisms

The majority of microbiome members most relevant to human health, such as *Clostridium* and other anaerobic Firmicutes, remain poorly cultivable and genetically intractable.^367^ This has hindered our ability to interrogate these microorganisms for mechanisms by which they modulate human health. Elucidating these mechanisms will not only deepen our understanding of host–microbiome and intramicrobiome interactions but also lead to more robust microbiome-based therapeutics that include these health-promoting bacteria. Thus, there is a great need for the development of novel genetic engineering tools that can be used in such genetically intractable microorganisms. These tools can range from well-characterized promoters, ribosome binding sites, terminators, and reporter genes to more complex genomic manipulation systems, such as CRISPR-Cas and homologous recombination.

In the following section, we will discuss how these challenges can be overcome with the aid of enabling technologies, advancing our understanding of microbiome–host interactions and the development of robust microbiome-based therapeutics.

## 4 Enabling Technologies for Microbiome Research and Engineering

Many studies including the examples mentioned in section 3 support the feasibility of engineered microbe therapy for microbiome-related diseases.^368−371^ However, despite many proof-of-concept studies, several clinical trials using engineered microbes could not show efficacy or were terminated due to a lack of efficacy in the Phase 2 trials (e.g., NCT03447730, NCT03234465, and NCT03447730). One contributing factor toward the lack of efficacy in these engineered microbes may be the absence of regulatory mechanisms. Typically, these mechanisms control the expression of exogenous genes under stable promoters in the microbiome to enhance efficacy or reduce side effects. One of the advantages of utilizing microbes as therapeutics is their high programmability compared to conventional chemical medicine. Synthetic biology approaches are expected to enhance efficacies and reduce side effects to leverage on the high programmability bestowed by the implementation of precise spatial/temporal control into engineered microbes.

To fully harness engineered probiotics for therapy, it is essential to keep in mind engineering approaches and designs based on a more sophisticated knowledge of the mechanistic insights of microbiome-associated diseases and the microbiome itself. As mentioned in section 3.1.1, the administration of pasteurized *A. muciniphila* to overweight/obese individuals improved insulin sensitivity and reduced cholesterol and body weight without changing the gut microbiome composition. Beyond reinforcing the importance of monitoring microbiome activities, this result suggests that the microbiome’s function or activity shifts can be more important compared to microbiome composition. Accordingly, functional meta-omics such as metabolomics, metatranscriptomics, metaproteomics, and multimeta-omics are gaining attention for investigating the causality of microbiome-associated diseases at the molecular, gene, and pathway levels.

In this section, we will review various meta-omics approaches for understanding molecular insights in the microbiome community and host interactions. We will also touch upon the many synthetic biology tools that can facilitate the reprogramming of microbes to potentially develop robust therapeutics with unique advantages over other strategies for microbiome engineering.

### 4.1 Functional Omics Approach

Metagenomics is a powerful tool for investigating the dynamics of microbiota, having revealed the association between microbiota composition and many diseases. Yet, metagenomic data provides limited mechanistic insights into microbiome-linked health states. To address this gap, applying functional meta-omics approaches such as metabolomics, metatranscriptomics, and metaproteomics to microbiota are expected to provide further insights. Here we will review the current progress in deploying functional omics to microbiota for investigating critical metabolites, microbiome activity, and interactions of host/diseases. Furthermore, we will review the microbiome and host genetics interactions.

#### 4.1.1 Discovery of Novel Metabolites and Biosynthesis

Interestingly, metabolites from microbiota play a more critical role in host–microbe interaction rather than the commensal microbe itself. However, the metabolites and biosynthesis pathways that underlie host–microbe interactions are still unclear. Several studies have shown that microbes interact with the host by modulating signal pathways via metabolites.^372^ For instance, SCFAs such as acetate, butyrate, and propionate are fermented in the colon from dietary fibers. These SCFAs are known to modulate the differentiation and accumulation of regulatory T cell (T_reg_ cell) by activating the G-protein coupled receptor.^373,374^ In turn, activated T_reg_ cells produce the anti-inflammation factor IL-10, which is assumed to suppress gut inflammation diseases like IBD. The link between microbiome composition and metabolites is known to be indirect due to the functional redundancy of metabolic pathways, which suggests the interchangeability of some species.^375^ Thus, analyzing differences in microbial composition may not reflect functional metabolite differences. In fact, phylogeny prediction from metabolomic data has been so far unsuccessful, indicating the difficulty of linking microbiome and metabolome data.^376^ For this reason, identifying the metabolites associated with disease can provide more straightforward data for helping implement synthetic approaches for therapeutic purposes.

Untargeted metabolomics using mass spectrometry has been used for the discovery of key disease-associated metabolites.^377,378^ Koh et al. employed untargeted metabolome analysis toward type 2 diabetes and discovered that imidazole propionate was present at higher concentrations among patients with type 2 diabetes.^377^ Imidazole propionate is produced from histidine by gut microbes and impairs insulin signaling through mTORC1. Previously, researchers identified the UrdA gene that produces imidazole propionate in vitro, finding that the UrdA gene was more abundant in subjects with type 2 diabetes.^377^ Another example is phenylacetylglutamine (PAGln), which was identified through untargeted metabolome analysis as a biomarker associated with a higher risk of cardiovascular diseases among type 2 diabetes patients. In the gut, PAGln is converted from dietary phenylalanine by the microbial porA gene, where it reportedly enhanced platelet activation-related phenotypes by stimulating G-protein coupled receptors.^378^

Because untargeted mass spectrometry identifies molecules by comparing the spectrum patterns of chemicals of interest to chemicals in a reference database, metabolite identification solely relies on the references used in the analysis. This means that untargeted identification is unable to pinpoint unknown chemicals. In fact, it is estimated that >90% of metabolites in the microbiome lack matches in public databases.^379^ Such uncharacterized metabolites are called “dark matter”.^379^ To tackle this problem, machine learning-based reference generation is gaining prominence. Machine learning is a method for automatically building mathematical models for classification or prediction, wherein an algorithm learns patterns from training data sets. Machine learning has been used across a wide variety of applications, proving its versatility.^380^ Currently, machine learning is being used for identifying microbiome metabolites during data preprocessing (peak detection, alignment, and identification), data processing (structure identification and compound quantification), and biological interpretation.^381^ For instance, DarkChem used a deep learning approach to generate MS/MS libraries for predicting chemical properties in metabolomics and chemical identification.^382^

#### 4.1.2 Metatranscriptomics and Metaproteomics

In metatranscriptomics, the transcriptional activities of microbiota are analyzed using RNA sequencing. Unlike metagenomics, metatranscriptomics allows for the identification of active microbes, genes, and pathways in microbial communities. Metatranscriptomics has since been applied to a number of different types of microbiotas,^383^ including those from seawater,^384,385^ soils,^386,387^ and human microbiota.^388^ Likewise, metatranscriptomics approaches in human microbiota have enabled a deeper understanding of host–microbiota interactions, active microbes and their pathways, and expression changes in disease progression.^389,390^ Nowicki et al. demonstrated how metatranscriptomics was applied to subgingival plaque from gingivitis patients. The study observed a significant shift of microbiota composition and increased virulence genes expression as gingivitis progressed,^389^ demonstrating the importance of transcriptomics analysis for understanding molecular mechanisms during disease progression. Meanwhile, Schirmer et al. performed metatranscriptomics on a longitudinal IBD cohort to elucidate gene expression and their differences among healthy and IBD patients.^390^ In the study, they detected species-specific biases in transcriptional activity. One example is the methylerythritol phosphate pathway (MEP) genes. They showed that *Bacteroides vulgatus* became the main transcriptional contributor of MEP at severe IBD stages. This study highlighted how metatranscriptomics analyses are a powerful tool for monitoring microbiome activities and gaining further insights into the role of the microbiome in diseases.

Although RNA expression can be a good indicator of gene expression, it does not always reflect protein abundances. Alternatively, metaproteomics can be engaged as an alternative approach for monitoring gene activity in microbiota. Metaproteomics was initially applied to investigate microbial function in environmental^391^ and gut microbiome samples from twins in a 2009 study.^392^ So far, multiple studies have demonstrated how metaproteomics analysis can be deployed for human microbiome samples.^393−395^ Although metaproteomics is not as common as metatranscriptomics because of its lower throughput compared to deep sequencer-based analysis, metaproteomics can provide information on the post-translational modifications of proteins^396,397^ and the expression of proteins secreted from the host cell,^398,399^ both of which cannot be monitored through metatranscriptomics.

Another study by Zhang et al. analyzed lysine acetylation (Kac) changes in proteins in the gut microbiome of patients with Crohn’s diseases and negative control subjects.^400^ In the study, they employed a peptide immune-affinity enrichment strategy followed by mass spectrometry to characterize Kac peptides and their changes in the human gut microbiome. Using the strategy, they identified Kac sites of 52 host and 136 microbial proteins that were differentially modified between Crohn’s disease patients and nonpatients. Likewise, Lobel et al. investigated the effect of diet on the post-translational modifications of proteins in the gut microbiome in chronic kidney disease (CKD) model mice.^397^ They discovered that a high sulfur amino acid-containing diet resulted in the post-translational modification of microbial tryptophanase. The protein modification reduced the production of uremic toxin in CKD model mice. These studies show that microbiome function can be altered via post-translational modifications without changing their composition, underlying the importance of metaproteomics for investigating mechanistic insights of microbiome-related phenotypes.

Given the advantages and disadvantages associated with meta-omics approaches, multimeta-omics approaches have been employed to comprehensively understand microbiota gene activities as well as interactions within microbiota or between the microbiota and host. One Human Microbiome Project team conducted a multiomics analysis on a longitudinal IBD cohort to elucidate the molecular profiles of the host and microbiome activity.^401^ In their study, they conducted metagenomics, metatranscriptomics, and metaproteomics on stool and serum samples from 132 subjects for 1 year. This study provided a comprehensive description of host and microbial activities, which helped identify microbial, biochemical, and host factors that contributed to dysregulation. Mills et al.^402^ combined metagenomics, metapeptidomics, metaproteomics, and metabolomics approaches from 250 fecal samples for ulcerative colitis (UC) studies. They discovered that proteinase activity derived from *B. vulgatus* was associated with UC severity. In vitro and in vivo experiments also showed that treatment with a proteinase inhibitor could suppress UC symptoms, highlighting the potential of a multiomics approach in identifying causal genes from complicated microbiomes.

#### 4.1.3 Microbiome Genome-Wide Association Study

Conventionally, it was held that interindividual variations in the microbiome composition were mainly influenced by environmental factors rather than the host’s genetic factors.^403^ However, evidence from twin^404,405^ and family^406^ studies have indicated the presence of interactions between the microbiome and host genetics. In a U.K. twin study, the relative abundances of gut microbiota were more highly correlated within monozygotic twins than dizygotic twins, suggesting that the interactions between the microbiome and host genetics influenced gut microbiota composition. A better understanding of host genetics and microbiome interactions can assist precision medicine approaches and enhance the efficacy of engineered microbe therapeutics.

A larger proportion of identified genetic loci linked to microbiome variance are related to the dietary preferences of hosts or their immunity. One of the most replicated genomic loci is the LCT locus. Studies across U.K., Dutch, Canadian, and Finnish populations have shown an association between the LCT locus and *Actinobacteria* or *Bifidobacterium*. LCT encodes lactase, which digests lactate in the gut. One of the strongest associations with Bifidobacteria in the LCT locus is the functional SNP rs4988235. This SNP is known to be strongly associated with lactose intolerance and lactase expression. Therefore, lactose intolerance is the inability to digest lactose caused by the lower expression of lactase. Bifidobacteria are known to have the ability to digest lactose,^407^ indicating that they compensate for reduced lactase activity in the host. Another well-replicated locus in microbiome composition is ABO. The association of the microbiome to the ABO locus was reported in studies conducted among German, Finnish, and Dutch populations. However, the bacteria associated with the ABO locus differs among the three countries. In addition to the ABO loci, FUT2 is also known to be associated with the microbiome, with FUT2 genes determining the ABO antigen on mucosal cells. The functional association between ABO and FUT2 suggests that the ABO locus is a contributing factor to microbiome composition. However, the mechanistic insights behind the microbiome changes have yet to be elucidated. Although dozens of genomics loci were reportedly associated with microbiota composition, most of the loci were not replicated in other studies.

Finally, host genetic variance can influence the host’s health states. One well-known example is the ATG16L1 locus. ATG16L1 encodes a subunit of the autophagy-related ATG12-ATG5/ATG16L1 complex and is involved in autophagosome formation. However, its function is not limited to autophagy; it is also involved in immune responses such as inflammation.^408^ Several genome-wide association studies (GWASs) have shown the association between IBD and the ATG16L1 locus.^409,410^ Chu et al. revealed that ATG16L1 is essential for immunomodulation by *B. fragilis*, which is known to secrete outer membrane vesicle (OMV) including immunomodulatory molecules that induce regulatory T cells.^411^ They showed that the risk allele of ATG16L1 (A300) did not show OMV-mediated regulatory T cell induction for mucosal inflammation suppression and also displayed a deficiency of ATG16L1. These results indicate host variant–microbiota interaction and the importance of considering host genetic factors for therapeutic purposes.

### 4.2 Synthetic Biology and Cellular Reprogramming of Microbes

Synthetic biology aims to design and engineer organisms with desired functions that are predictable and consistent. To achieve this aim, synthetic biology introduces into biology engineering principles such as modularization, logic gate, and circuit design.^412,413^ These efforts enable researchers to choose optimal genetic parts (e.g., promoter, ribosome binding site, terminator, peptide tag, and biosensor) as well as construct all types of logic gates^414^ (AND, OR, NOR, NOT, XOR, and NAND) and synthetic devices that can control microbes similar to machines like the oscillator^415^ and genetic toggle switch.^416^ In turn, these well-characterized and tunable parts allow the assembly of more functional systems through an optimization cycle called the design–build–test–learn cycle (DBTL cycle). In this subsection, we will review how synthetic biology can be used for cellular reprogramming. We will also discuss how synthetic biology allows the spatiotemporal regulation of engineered microbes and how such techniques can be applied to microbiome environments.

#### 4.2.1 Regulating Microbe Behavior Using Genetic Logic Circuits

**Figure 5:**
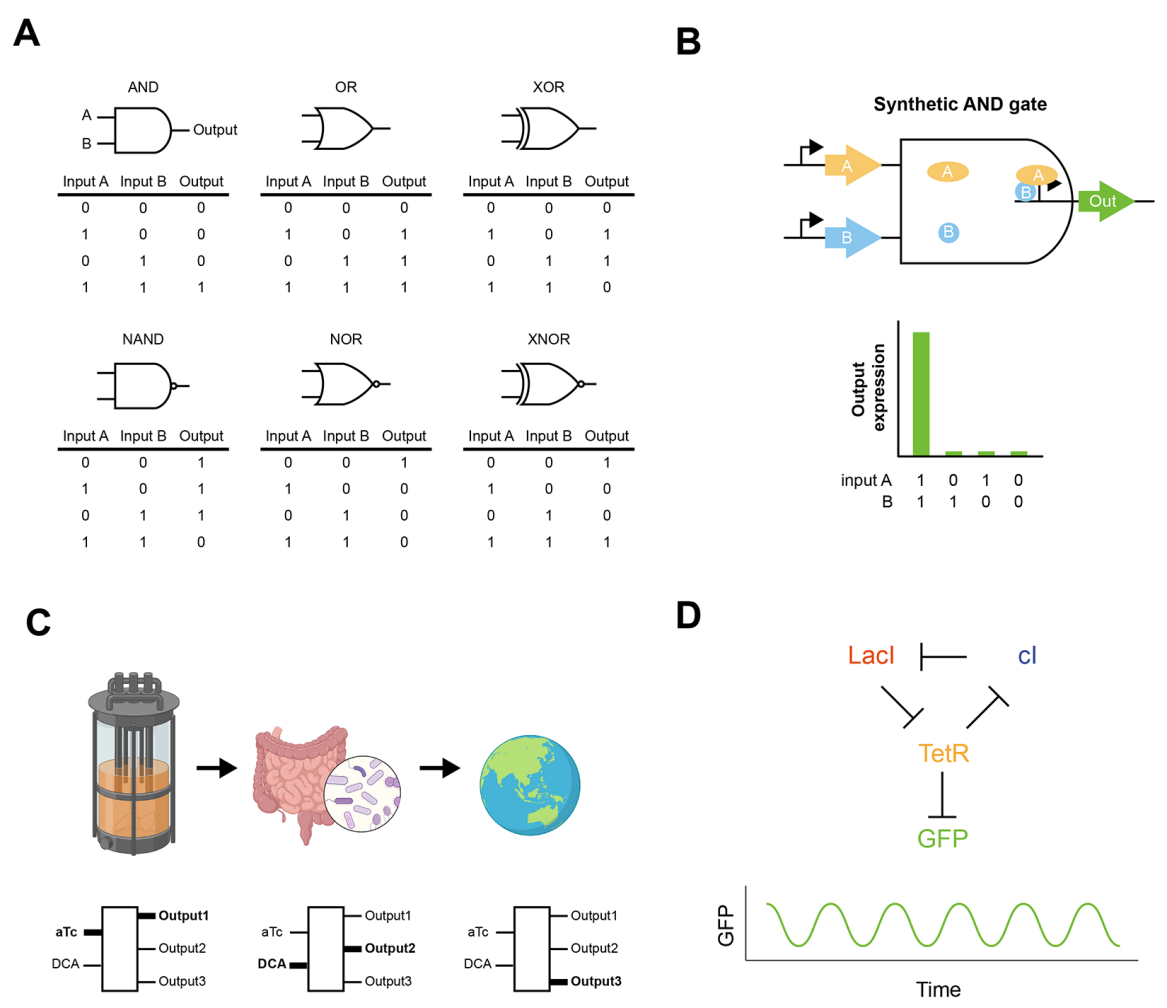
(A) Logic gates and their truth tables. (B) One example of the transcriptional AND gates.^417^ The output gene expression is activated only when both A and B are induced. (C) Application of synthetic logic gates for the spatiotemporal control of engineered microbes. (D) Transcriptional network found in repressilator. TetR represses cI, cI represses LacI, and LacI represses TetR.

A logic gate is an electronic device that performs a basic logical function by processing binary inputs to binary outputs. Most logic gates process two inputs into one output following the truth table (Figure 5A). For example, AND gate outputs 1 only when both inputs are 1 and OR gate outputs 1 when either input is 1. Each logic gate can operate a simple task. However, devices with multiple logic gates can process complicated tasks like a computer. Synthetic biology has enabled the development and implementation of genetic logic gates by mainly connecting transcription networks using transcription factors. In synthetic logic gates, expression represents 1 and no expression represents 0. An AND gate can be constructed by using a promoter that requires two transcription activators for its transcription (Figure 5B). The AND gate can be a very powerful and useful tool for modulating inputs and an output. For example, Merk et al. constructed a genetic AND gate that expresses output genes when both ITPG and tetrathionate exist.^418^ Because IPTG is an artificial inducer and tetrathionate is gut inflammation marker, this AND gate allows for the temporal and spatial regulation of the expression of specific genes. While GFP was used as an output in their proof-of-concept study, the AND gate can be used to temporally or spatially control the in situ secretion of medicine by changing the output accordingly.

In addition, such logic gates are scalable and can accommodate an increase in the number of outputs and inputs by connecting multiple logic gates that can process more complicated environments. Taketani et al. developed a 2-input and 3-output logic circuit by combining three NOR gates. This circuit was then implemented in *Bacteroides thetaiotaomicron*, enabling the bacteria to express different genes in an environment-dependent manner such as the bioreactor stage, the gut stage, and after release from the host.^417^ In this study, they employed anhydrotetracycline (aTc) and the bile acid deoxycholic acid (DCA) as inputs and demonstrated how the engineered *B. thetaiotaomicron* changed its behavior in an input-dependent manner in a human gastrointestinal model. These proof-of-concept studies show that scalable logic gates allow for the spatial and temporal regulation of engineered microbes under complicated environments. Therefore, administering standalone engineered microbes that behave in an environment-dependent manner improves the specificity and localization of their activity, improving overall efficacy.

An oscillator is an electronic device that outputs a periodic signal. Elowitz and Leibler constructed a genetic oscillator that expresses a marker gene periodically.^415^ In the original genetic oscillator, three transcription repressors, LacI, TetR, and cI, were encoded in one plasmid called repressilator, and GFP was encoded under pLtetO1 in the other plasmid. Because TetR represses cI, cI represses LacI, and LacI represses TetR expression, the expression of each gene changed periodically. The GFP reporter gene was regulated by TetR, and its expression also changed periodically. Although the original repressilator showed periodic GFP expression, only 40% of cells were found to behave properly. Accordingly, the experiment was found to not be robustly designed—causing error propagation by stochastic effects. A follow-up study done by Potvin-Trottier et al. improved the original by removing the degradation tags attached to the repressors and introducing a “sponge” sequence that soaked up TetR molecules.^419^ These changes reduced stochastic effects and improved the robustness of the genetic oscillator. Recently, an updated version of the oscillator was tested by Riglar et al. in a mouse gut environment to quantify bacterial dynamics in vivo.^420^ They developed the RINGS (repressilator-based inference of growth at single-cell level) method to estimate bacterial generations after synchronization. By using the RINGS method, they succeeded in doing so in the mouse gut environment to understand in vivo bacterial dynamics. In the study, an oscillation system was used to monitor bacterial dynamics in vivo, but the oscillation system could be utilized for the periodic administration of drugs in situ as well. Moreover, these studies highlighted how synthetic genetic circuits can function properly in microbiome environments through the DBTL cycle.

The examples of genetic circuits presented earlier demonstrate how microbes can be reprogrammed to exhibit desired behavior using different genetic parts, such as promoters, repressors, and activators. In the following sections, we will review different types of cellular reprogramming that can potentially improve the efficacy of microbial therapies for microbiome-associated diseases.

#### 4.2.2 Biosensors and Quorum Sensing

To process environmental information, microbes can be equipped with biosensors that can detect pH, temperature, light, metals, and chemical and biological compounds, among others.^421^ In synthetic biology, biosensors are integrated into synthetic genetic circuits to detect and process environmental information for downstream processing, which allows engineered microbes to change their behaviors in an environment-dependent manner and to communicate among bacterial communities.

To engineer therapeutic microbes, implementing biosensors into genetic circuits can provide several advantages. First, biosensor-based expression can reduce genetic burden and improve the genetic stability of engineered microbes. It is well-known that implementing a synthetic genetic circuit can exert a burden on microbes,^422^ resulting in genetic mutation, a loss of engineered function, and growth defects in the engineered microbes.^420^ Most of the genetic burden arises from the consumption of cellular resources, reducing cellular fitness.^423^ Therefore, silencing genetic circuit activity through biosensors can reduce genetic burden and keep engineered microbes functional. Another approach for reducing genetic burden is by cooperating multiple engineered microbes via quorum sensing (QS).

Second, biosensor-based expression can reduce the risks of off-target and resultant side effects. All medications have side effects mainly due to their dosage or unwanted targeting. Biosensor-based expression control has often been utilized for cancer therapy development, which requires specific targeting to reduce severe side effects. One common strategy is to express anticancer products under a hypoxia promoter from bacteria that can colonize tumor microenvironments, such as *Salmonella typhimurium*, *Clostridium novyi*, and EcN. He et al. engineered EcN to express Tum-5 or p53 under oxygen-dependent P_vhb_ promoters,^424,425^ which activates transcription under hypoxic areas such as the tumor microenvironment. In the study by He’s team, engineered EcN was injected into mice bearing tumors. They confirmed that the engineered EcN could repress tumor growth and that no obvious side effects could potentially occur from nonspecific targeting. More research regarding reprogramming microbes for cancer treatment has been reviewed elsewhere.^426^ Another promising target for engineered microbes with biosensors is pathogens. As discussed in section 3.1.2, employing QS machineries and antimicrobial agents enable the engineering of microbes that sense and eliminate specific bacteria secreting QS signal molecules. Third, biosensors can be used for diagnostic tools by combining memory systems, which will be reviewed in the next section.

Biosensor specificity is crucial for the proper function of engineered microbes, especially in complex heterogeneous environments, such as the human gut, which contains several structurally similar ligands. However, wild-type biosensors are often nonspecific and react to several structurally similar molecules. Hence, it is necessary to develop biosensors that can differentiate between such ligands in vivo to ensure an accurate response to the disease. Meyer et al. employed directed evolution to engineer 12 highly specific biosensors with lower cross-reactivity.^427^ Recently, Rottinghaus et al. engineered EcN for the specific sensing of aromatic amino acids or neurochemicals through the rational improvement of biosensor specificity based on the protein structures.^428^ Such studies are expanding the toolbox of high-quality biosensors for therapeutics purposes.

#### 4.2.3 Memory Systems

Cellular memory is a phenomenon wherein transient signals are converted into a prolonged response. Cellular memory is common in most organisms and is used widely in biological events such as differentiation,^429^ epigenetics,^430^ and immunity.^431^ Due to its potential applications, many types of synthetic memory circuits have been constructed based on various mechanisms^432^ such as transcription factors,^416^ DNA recombination,^433^ and RNAi.^434^

**Figure 6:**
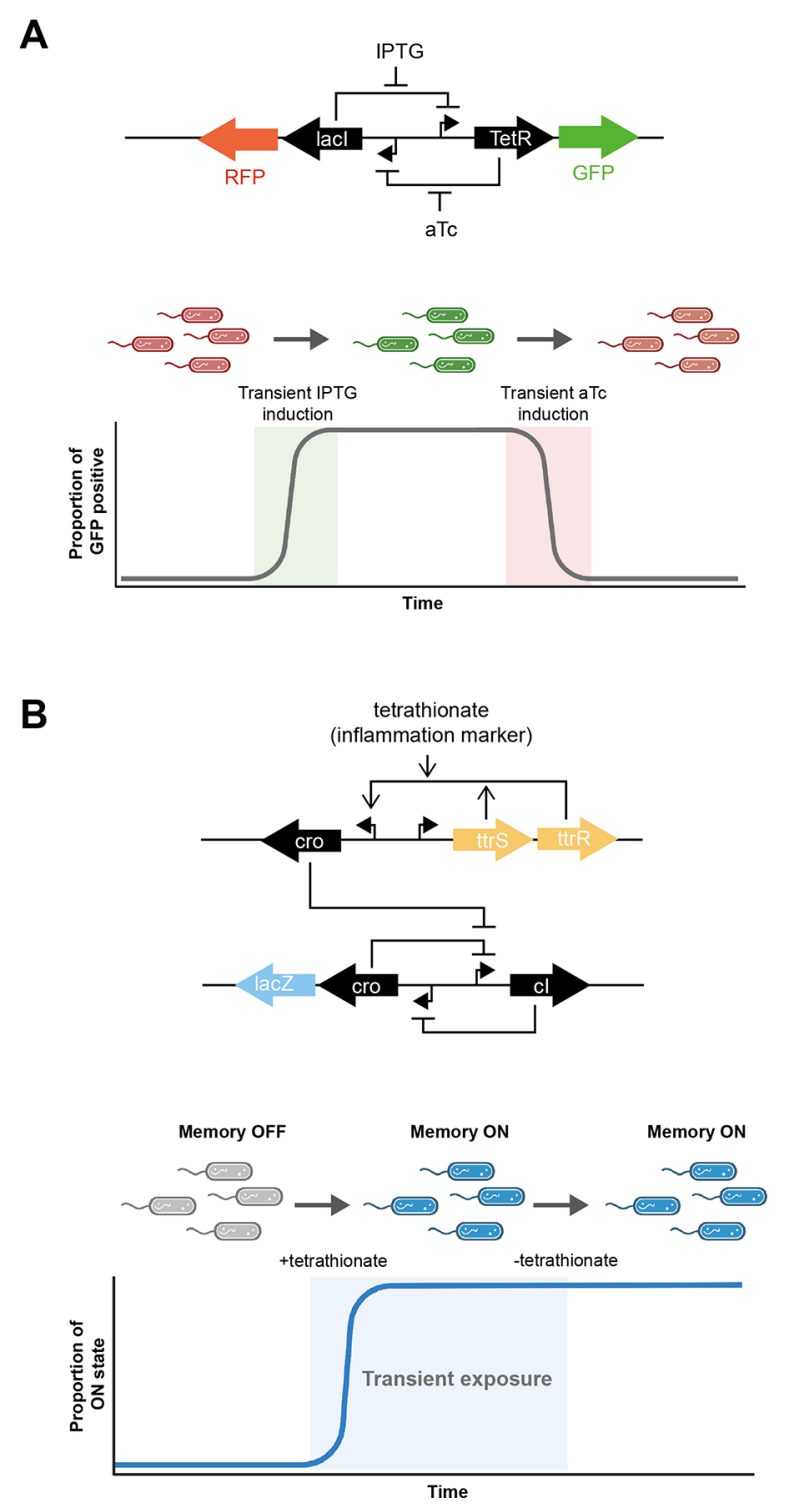
(A) Gene circuit of the genetic toggle switch developed by Gardner et al.^416^ TetR and lacI each repress transcription. Either tetR or lacI expression is dominant without inducer. Each inducer (aTc or IPTG) inhibits a repressor and induces expression. Once cells are exposed to an inducer, either lacI or tetR become dominant. (B) Toggle switch-based memory circuit for a gut inflammation diagnosis tool. Cro is activated upon the detection of the inflammation marker tetrathionate by the ttrS/ttrR component. Once the circuit has detected tetrathionate, expression of cro becomes dominant even after tetrathionate withdrawal.

Memory systems can be classified as either reversible or irreversible. Reversible memory systems can be turned off upon the detection of another signal. Gardner et al. constructed a genetic toggle switch that can function as a reversible memory system by implementing LacI and cI or TetR to mutually inhibit their expression (Figure 6A).^416^ Because repressors mutually inhibit expression, coexpression is at an unstable steady state and the expression of either repressor becomes randomly dominant in each cell without any stimulus. Gardner et al. also demonstrated that temporal exposure to inhibitors of the repressors could switch the cellular state and that the state lasts long after inhibitor withdrawal. On the other hand, an irreversible memory system cannot be turned off once a signal is detected. O’Gorman et al. developed an irreversible memory system utilizing Flippase that excises the reporter’s cis-element after a specific stimulus and keeps reporter gene expression on in a mammalian cell.^435^

Synthetic memory circuits have proven to be a useful research tool for investigating fundamental biological mechanisms and as a potential tool in medicine and industry. For instance, the Cre-loxP system for creating tissue-specific knockout strains is an irreversible memory system^436^ for investigating gene function in specific organs and tissue because it does not perturb gene function in other organs and tissues. The system is also used to trace the developmental lineage of cells.^437^ For industrial applications, synthetic memory circuits can be used to reduce the cost of inducers for constant induction to produce biochemicals of interest.^432^ By combining biosensors, synthetic memory systems can be used as noninvasive diagnosis tools for gut microbiome-associated diseases. Kotula et al. demonstrated that a synthetic memory circuit using a cI/cro bistable genetic switch could function in vivo.^438^ In a follow-up study done by Riglar et al.,^420^ they developed a diagnosis tool for gut inflammation by implementing a memory system and a biosensor to detect the inflammation marker tetrathionate in the *E. coli* strain NGF-1 (Figure 6B). They used the TtrR/TtrS two-component system to sense tetrathionate and trigger the memory device and cI/cro bistable genetic switch for a memory device. This may allow the detection of disease onset in intestinal organs before symptoms worsen.

Biosensors are an essential building block for processing environmental cues in synthetic biology approaches. However, the number of biosensors that can monitor the inner states of the microbiome are still limited. To overcome this problem, Naydich et al. developed a high-throughput memory system than can be used to screen for biosensors that function in murine gut.^439^ They implemented a cI/cro bistable toggle switch as a memory device. In the memory off state, the cI repressor is dominant, while cro and downstream lacZ expression is off. The trigger device is composed of a dominant negative mutant of cI (cI^DN^) under a candidate promoter that is triggered by the condition of interest. cI^DN^, which has an N55K mutation in its DNA-binding region, forms a dimer with wild-type (WT) cI and derepresses cro and lacZ expression. Once cro is expressed highly enough, cro can keep repressing cI even without a stimulus. Hence, by generating a trigger device library, a high-throughput memory system can be used to screen for promoters that are active during the condition of interest. They tested this high-throughput memory system to screen for promoters with an increased response to an inflamed murine gut environment. This research exemplifies how synthetic biology approaches can be used as an investigation tool.

#### 4.2.4 Kill Switches for Biocontainment and Drug Delivery

As research on developing microbes as therapeutic agents has progressed, issues relating to the biosafety of genetically modified organisms have emerged, raising concerns over the increased risk of spreading potentially hazardous biological materials to environments. Hence, the implementation of effective biocontainment systems is essential for real world usage, especially in cases where engineered wild-type commensal microbes are used due to their resilience in wild environments compared to commonly used laboratory strains. Many biocontainment strategies have already been developed through synthetic biology approaches.^440^ The most readily used strategy is to introduce auxotrophic mutations, in which microbes are engineered to be dependent on specific nutrients for growth. However, auxotrophic strains may survive in natural environments that provide the nutrient. Moreover, this strategy can be used only for microbes that can be isolated and cultured in vitro. Therefore, the kill switch can be an alternative strategy for biocontainment.

**Figure 7:**
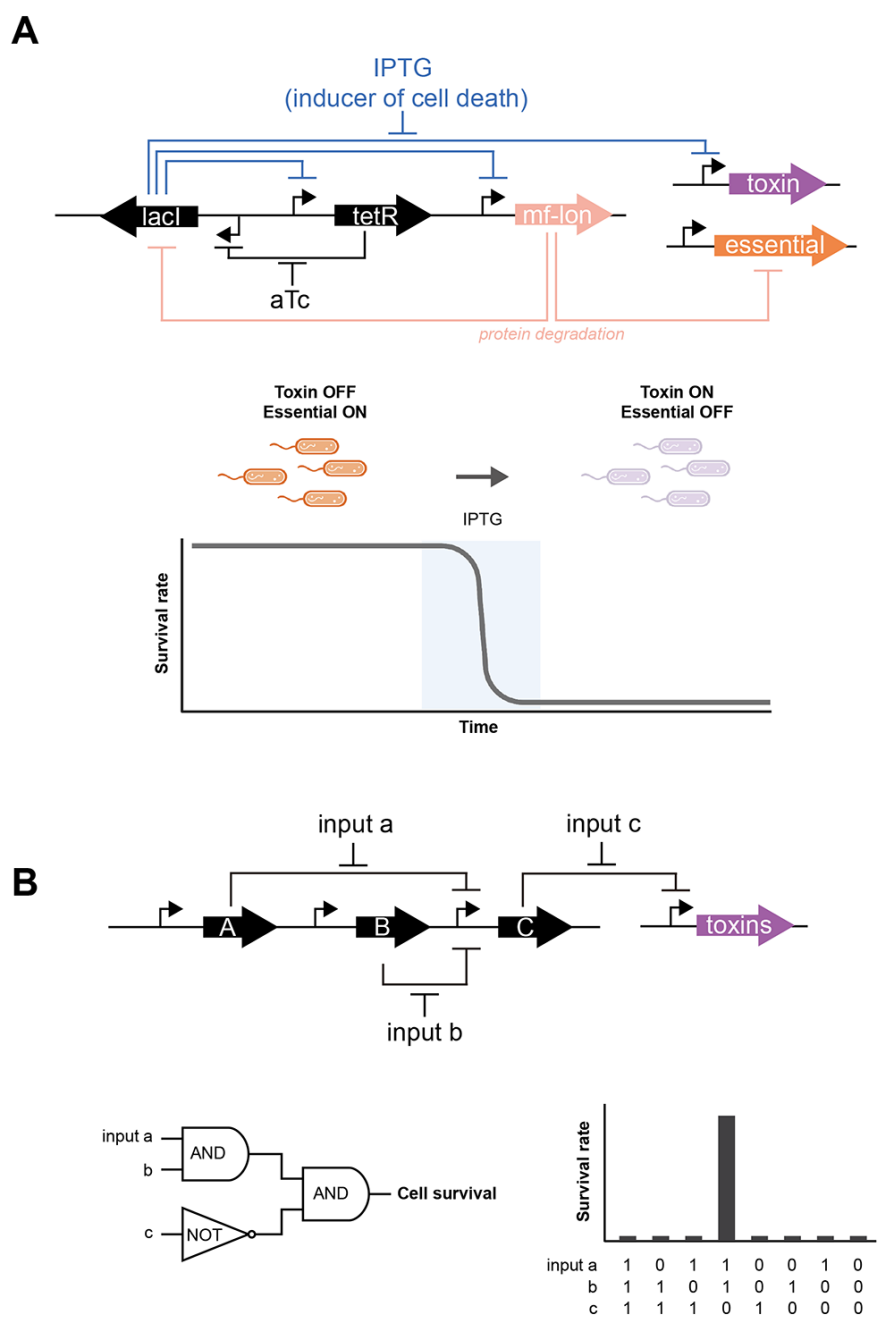
(A) Genetic circuit of deadman kill switch. Output of the toggle switch activates a toxin and inactivates an essential gene to kill cells, which can be induced by IPTG. Mf-lon proteinase then degrades lacI and essential genes to increase circuit stability. (B) Genetic circuit of passcode kill switch. A, B, and C represent repressor genes. The loss of input a or b or the addition of input c activates the toxin gene expression.

Kill switches have long been used in synthetic biology. In 1987, Molin et al. developed a conditional suicide switch by expressing the Hok gene under the Trp promoter repressed by tryptophan.^441^ The Hok gene causes the depolarization of the cellular membrane and results in cell death. They showed that Hok can work in broad ranges of both Gram-positive and Gram-negative bacteria. Contreras et al. utilized the nuclease gene from *Serratia marcescens* for a suicide gene combined with a thermo-induction promoter.^442^ In 2016, Chan et al. developed the “deadman” and “passcode” kill switches,^443^ which are passively activated. The deadman switch activates the toxin gene and inactivates an essential gene in the absence of a signal (ATc). To increase robustness, a genetic toggle switch was implemented in the deadman switch (Figure 7A). On the other hand, the passcode death switch employed the hybrid LacI-GalR family TFs^444,445^ to allow multiple input molecules that control cell death (Figure 7B). They showed that the loss of IS1 and IS5, which caused a large percentage of inactivating mutations in the passcode circuit after long-term culturing, increased the passcode’s stability.

Due to the kill switch’s lethality, engineered cells always have a selection pressure to eliminate the kill switch.^443^ Hence, improving the genetic stability of the kill switch is essential for proper and effective function. It was reported that mutagenesis is one of the major reasons why an engineered circuit loses function.^443^ Thus, restoring the mutation to its original sequence may help increase stability. Chavez et al. developed a mutation-restoring system using CRISPR/Cas9.^446^ In their system, gRNAs were designed to recognize specific mutations and convert the mutations back to their original functional sequences. They demonstrated that this system worked in a murine gut environment and reduced mutation frequencies drastically. Although this system can prevent specific mutations only, this method can be potentially applied to prevent hotspot mutations causing the loss of function of kill switches.

Another approach to improve the kill switch’s stability is functional redundancy within the circuit. Rottinghaus et al. engineered EcN with an aTc-inducible kill switch, which induces the expression of gRNA and Cas9 to cut the EcN genomic DNA.^447^ They demonstrated that ∼10% and ∼80% of loss-of-function mutations in the kill switch were accumulated at the gRNA and the Cas9 cassettes, respectively. To decrease the probability of losing Cas9 and gRNA functions, they employed four functionally redundant Cas9 cassettes and two gRNA cassettes to achieve a 10^–8.6^ killing efficiency, which surpassed the recommended killing efficiency of 10^–8^ by the National Institutes of Health.

Another application of the kill switch is in the release of functional compounds from cells to target cells, e.g., pathogens, inflammation, and cancer cells in situ. Although proteins can be designed to be secreted by adding a secretion signal, nonpermeable small compounds require a specific transporter or channel to be exported from cells. Because those transporters and channels are not available in many cases, the kill switch can be a versatile alternative method to secrete any compounds, including large and small molecules. Saeidi et al.^448^ demonstrated the feasibility of using a kill switch for secretion purposes. In the study, a sensor device that sensed *P. aeruginosa* through quorum sensing by the LasR protein activated cell lysis and released the Pyocin S5 protein to kill *P. aeruginosa*. However, this method still carries the risk of releasing the genetic material introduced into the engineered microbes to the environment

### 4.3 Chassis Engineering

To facilitate cellular reprogramming of the desired microbe, the relevant genetic circuit has to be introduced into the microbe. Such microbes are typically referred to as the “chassis”, with the term based on the structural framework of equipment and machines like automobiles. Accordingly, the microbe is usually transformed with plasmids to introduce new functions without changing its genomic DNA. In fact, many early proof-of-concept studies were done through plasmid-based approaches.^415,416^ Still, problems with the plasmid-based approach include the lower stability of plasmid DNA as well as expression noise caused by copy number differences among cells. Hence, the integration of genetic parts or devices into the chassis’ genomic DNA is preferable, especially for therapeutic purposes. Moreover, chassis engineering enables the removal or enhancement of the organisms’ features to engineer a more suitable chassis for therapeutic purposes. Despite the advantages of chassis development, the genetic manipulation of commensal microbes still lags behind model organisms such as *E. coli* and *S. cerevisiae*—both of which are not major members of the human microbiome. In this subsection, we will review current developments in CRISPR-based gene manipulation of microbes and in situ DNA transfer methodologies, which allow for the genetic modification of genetically intractable microorganisms.

#### 4.3.1 CRISPR-Based Gene-Editing/Manipulation Tools

CRISPR was originally discovered as an immune system in archaea, although nowadays it is recognized as a genetics tool. CRISPR is mainly applied for gene editing through the introduction of DNA breaks, followed by homologous recombination using donor DNAs. CRISPR-directed homologous recombination has accelerated genome engineering in many organisms including those with genomes previously considered difficult to manipulate.^449^ Currently, CRISPR tools are available in a wide range of commensal bacteria and yeast such as *E. coli*,^450^*Lactobacillus*,^451^*Clostridium*,^452^*Bacteroides*,^453^*Staphylococcus*,^454^*Bacillus*,^455^*Saccharomyces*,^456^ and *Candida*(457) for site-directed mutagenesis and gene deletion/insertion. CRISPR has also been used to engineer the microbiome and commensal microbes for characterizing the gene function of microbiome-related phenotypes. Guo et al. employed CRISPR/Cas9 to demonstrate the potential link between host immunity response and SCFAs produced by *Clostridium*.^367^ In the study, Guo et al. developed a CRISPR/Cas9 system for manipulating *Clostridium sporogenes* and deleted SCFA-related genes, resulting in the decreased SCFA production. By comparing immune responses between mice administered with wild-type strain and knockout strains, they showed that the loss of SCFA increased IgA plasma cells. This supports the immunomodulation function of SCFA. Ultimately, the study showed that CRISPR-based microbiome genetics can help identify causal genes and their interactions, which are required in synthetic biology approaches.

Although CRISPR-driven gene editing is widely available for many organisms, DNA breaks caused by CRISPR/Cas9 tend to lead to cell death rather than gene editing in a majority of commensal microbes that have limited homologous recombination activity. Hence, CRISPR/Cas9 cannot be used for the majority of nonmodel commensal bacteria. For these microbes, CRISPRi, CRISPRa, or base editors can be less-toxic alternatives. CRISPRi and CRISPRa are tools that modulate transcription activity rather than edit genes. Both tools employ dCas9, which has DNA binding activity rather than DNase activity, fused to a transcription repressor or activator, respectively, to modulate transcription near the loci sgRNA recognizes. Hence, CRISPRi and CRISPRa function as programmable transcription factors that ideally can target promoters of any gene. Their programmability allows knockdown screening for bacteria using CRISPRi libraries.^458^ Accordingly, Peters et al. constructed a CRISPRi library that covers all of the essential genes of *B. subtilis* for functional screening and identifies the mechanism of action of antibiotics.^458^

Due to the high programmability of CRISPRi and CRISPRa, they can be used as custom transcription factors for constructing genetic circuits. A number of CRISPR-based synthetic gene circuits have since been constructed.^459^ Many logic gates, buffers, NOT,^460^ AND,^436,461,462^ OR,^462^ NAND,^462^ NOR,^436,462,463^ XOR,^436^ and NIMPLY^436^ were successfully implemented using CRISPRi and CRISPRa. In addition to synthetic logic gates, synthetic gene circuits such as the bistable toggle switch,^464^ oscillator,^464^ and stripe pattern generation^464^ were also implemented using CRISPRi and CRISPRa. So far, while most genetic circuits constructed by CRISPRi and CRISPRa are in *E. coli* or *S. cerevisiae*, this approach can also help construct genetic circuits in nonmodel organisms.

While CRISPR/Cas9 gene editing introduces mutations through DNA strand breaks and subsequent homologous recombination, base editors mutate DNA using deaminase. Base editors employ dCas9 or nickase Cas9 fused to a deaminase that can convert nucleotides.^465,466^ For instance, the cytosine base editor converts C to G,^466^ while the adenine base editor facilitates A to T conversion.^465^ Because base editing is quite new, there is no study yet utilizing it for microbiome engineering. However, microbiome editing using base editors is expected to soon be applied in therapeutics due to its lower toxicity compared to using bacteria.

Another promising CRISPR-related tool for microbiome engineering is the CRISPR-associated Tn7 transposon (CAST).^467,468^ CAST was first reported in 2017 by Peters et al. They reported that some bacteria carry a Tn7-like transposon, including Cas effector proteins of the CRISPR subtype I-F. The Tn7-like transposon lacks TnsE, which mediates target insertion of the Tn7 transposon. Hence, they hypothesized that this type of transposon hijacked and utilized the CRISPR system for DNA recognition,^467^ with CAST transposing through a CRISPR-mediated manner. In 2019, two groups proved that CAST inserted into the target site in a CRISPR-mediated manner in *E. coli*.^469,470^ Both showed that the target site can be reprogrammed by changing the gRNA sequence. Strecker et al. showed that DNA insertion efficiency can reach up to 80% without any selection, with the frequency of off-target insertion being <1%. According to studies, insertion efficiency is highly dependent on target sites. Strecker’s group also detected on-target insertion in 29 loci out of the 48 sites tested. In 2022, Rubin et al. applied CAST to develop the DNA-editing all-in-one RNA-guided CRISPR–Cas transposase (DART) system, which was site- and species-specific in a mouse gut microbial community.^471^ This will be reviewed in the next section.

All in all, CRISPR enables the manipulation of a wide variety of genomic DNA. DNA delivery remains the first step in experimental manipulation for downstream processes, yet the majority of commensal microbes are not culturable. Consequently, in the next section, we will review the current progress of gene manipulation of microbiota in situ.

#### 4.3.2 Genetic Manipulation of Microbes In Situ

Multiomics studies have uncovered how microbiota genes are associated with many human diseases and health states, as mentioned in section 2. However, elucidating the causal relationship of microbiota genes to host disease remains tricky mainly due to the difficulty of genetic manipulation in nonmodel microbiota as well as the challenges involved in culturing them in vitro.^472^ Consider how conventional DNA delivery methodologies such as chemical transformation and electroporation can only be applied in vitro. Therefore, alternative technologies for transferring DNA into microbiota in situ are gaining traction in microbiome engineering. In this section, we will review the current progress of DNA delivery technologies and commensal bacteria manipulation methods in situ.

To adapt to various conditions, environmental bacteria are known to actively exchange their plasmid DNAs among different species—a process called horizontal gene transfer (HGT).^473^ One of the most widely used HGT methods is bacterial conjugation, where plasmid DNA is transferred from a donor bacteria to a recipient bacteria through a type IV secretion system.^474^ The gut microbiota is considered a fertile environment for conjugative gene transfer. It has been reported that bacterial conjugation can be utilized to manipulate gut microbiota from various donors in situ.^475,476^ Recently, bacterial conjugation has become increasingly prominent as a microbiome engineering tool. Ronda et al. developed a technique called metagenomic alteration of gut microbiome by in situ conjugation (MAGIC), which enables the transfer of plasmid DNAs from an *E. coli* donor strain using the Inc.Palpha-family RP4 conjugation system to gut microbiota in situ.^477^ This system allowed them to introduce DNA to both Gram-positive and Gram-negative bacteria. The conjugation plasmid encoding the transposon and transposable cassette allows the integration of DNA into genomic DNA in situ. Fluorescence-activated cell sorting (FACS) and 16S RNA analysis showed that at least 5% of gut bacteria were successfully modified in situ. However, transconjugants were no longer detectable after 72 h, likely due to toxicity or vector instability. Consequently, further improvements are needed for MAGIC to be used in the stable genome manipulation of gut microbiota for the investigation and identification of causal genes in microbiome-associated diseases.

Another method for transferring DNA into bacteria in complex communities in situ is by using phages. Phages are viruses that infect specific bacteria and transfer their genomic DNA into bacterial cells. After infection, phage-derived plasmid DNA is integrated into host genomic DNA or replicated in the host. By cloning a desired DNA fragment into the phage’s genomic DNA, exogenous genes can be transferred to bacterial cells, where they confer new functions with a fairly high efficiency. Notably, the transduction efficiency of the P2 bacteriophage can reach nearly 100%,^478^ far beyond other DNA delivery methods like chemical transformation or electroporation. Because of target specificity, high efficiency, and activity in situ, phages are now being applied in microbiome engineering.^479,480^ For instance, Citorik et al. utilized a phage to deliver CRISPR/Cas9 with a gRNA targeting the antibiotic resistance gene in a synthetic *E. coli* population in waxworms and changed bacteria composition.^481^ In addition, Lam et al. demonstrated that phages could deliver a CRISPR/Cas9 cassette to *E. coli* in the murine gut,^294^ showing that CRISPR/Cas9 delivered by a phage in situ could cause a chromosomal large deletion.

Knowing genetically tractable microbes and choosing an optimal DNA transfer method for microbes of interest in situ are crucial because there is no versatile DNA transfer method for all commensal microbes. Rubin et al.^471^ developed the environmental transformation sequencing (ET-seq) and DNA-editing all-in-one RNA-guided CRISPR–Cas transposase (DART) systems, techniques that allowed the identification of genetically tractable bacteria in microbial communities and organism- and locus-specific genetic manipulation in situ. In ET-seq, DNA containing a nontargeted *mariner* transposon was transferred to microbial communities by conjugation, electroporation, or natural transformation, after which transposon-integrated loci were identified by deep sequencing. Following the identification of tractable bacteria and an optimal DNA delivery method, organism- and locus-specific CRISPR-associated transposase plasmids were designed and introduced into soil and infant gut microbiota. Using DART, they targeted strain-specific genomic loci of *E. coli* and demonstrated that DART could change *E. coli* strain composition. Another example of the optimization of DNA manipulation was done by Jin et al. They developed a pipeline to manipulate nonmodel gut microbiota (*Clostridia*) in vitro and in the host.^303^ Their pipeline included the identification of compatible rep and ori combinations for the vector, antibody selection for both *E. coli* and *Clostridia*, and, finally, the reduction of restriction modifications to increase stability for stable conjugation and genome modification.

## 5 Challenges and Limitations

In section 2, we reviewed microbiome studies that showed the link between the microbiome and disease/health states. These studies suggested that the microbiome plays a crucial role in human health through microbiome–host interactions. Meanwhile, in section 3, we reviewed how compositional and functional alterations of the microbiome could affect human health states, indicating that the microbiome can be a contributing factor to several diseases. Revealing the causality of microbiomes in diseases enables its modulation by various strategies. However, microbiome engineering therapies have not yet yielded a viable commercial product. Although we are awaiting the results of clinical evaluation in some cases, other therapies, particularly engineered bacteria therapies, have been unable to perform well in clinical trials so far due to lack of efficacy.

Accordingly, we propose three limitations hindering the feasibility of leveraging engineered microbiome therapy for diseases: (1) the lack of mechanistic understanding underlying microbiome-associated diseases at multiple levels; (2) the challenges involved in modifying genetically intractable organisms; and (3) the spatiotemporal regulation of the engineered microbiome. In section 4, we reviewed the current progress of meta-omics studies and synthetic biology tools that can help resolve these limitations. In particular, metabolomics, metatranscriptomics, metaproteomics, and multimeta-omics approaches enable association studies at the metabolite, gene, and gene interaction levels—deepening our knowledge of the molecular mechanisms of microbiome-associated diseases and allowing the design of reprogrammed microbes with predictable and regulated behavior against specific targets. Moreover, CRISPR and in situ DNA transfer technologies using bacterial conjugation and phages permits the manipulation of genetic intractable microbes both in vitro and in situ. Advances in such new DNA manipulation technologies will help in the identification of responsible genes and the engineering of commensal microbes in vitro and in situ.

We also reviewed how common design principles in synthetic biology taken from engineering fields facilitate spatiotemporal regulation in engineered microbes with synthetic genetic circuits. Although most experiments were performed in in vitro conditions, some were applied in the gut microbiome, proving the feasibility of spatiotemporal regulation within the microbiome environment.

As mentioned earlier, functional meta-omics and synthetic biology approaches help resolve the limitations hindering the feasibility of engineered microbe therapy. However, experimental tools must still be specifically tuned for studying the microbiome, as synthetic biology emerged independently of microbiome studies. Hence, most available tools are designed for *E. coli* to work under constant laboratory conditions. To date, a collection of standardized genetic parts for the nonmodel commensal microbiome remains limited or is unavailable. For instance, *E. coli* genetic parts were originally developed for in vitro purposes; hence, most of their functionalities in microbiome environments are not well-evaluated and may cause the loss of robustness in genetic circuits. A robust design is crucial for therapeutic applications due to the diversity of the microbiomes found in individuals. Therefore, the optimization of genetic parts and gene circuits for in vivo environments is set to be accelerated by developments in in vitro platforms such as organs-on-a-chip and organoids.

## References

[ref1] Hooper L. V.; Gordon J. I. Commensal Host-Bacterial Relationships in the Gut. Science 2001, 292, 1115–1118. 10.1126/science.1058709. 11352068

[ref2] Theis K. R.; Dheilly N. M.; Klassen J. L.; Brucker R. M.; Baines J. F.; Bosch T. C.; Cryan J. F.; Gilbert S. F.; Goodnight C. J.; Lloyd E. A.; et al. Getting the Hologenome Concept Right: An Eco-Evolutionary Framework for Hosts and Their Microbiomes. mSystems 2016, 1, e00028-16 10.1128/mSystems.00028-16. PMC5069740 27822520

[ref3] Simon J. C.; Marchesi J. R.; Mougel C.; Selosse M. A. Host-Microbiota Interactions: From Holobiont Theory to Analysis. Microbiome 2019, 7, 5 10.1186/s40168-019-0619-4. 30635058 PMC6330386

[ref4] Berg G.; Rybakova D.; Fischer D.; Cernava T.; Verges M. C.; Charles T.; Chen X.; Cocolin L.; Eversole K.; Corral G. H.; et al. Microbiome Definition Re-Visited: Old Concepts and New Challenges. Microbiome 2020, 8, 103 10.1186/s40168-020-00875-0. 32605663 PMC7329523

[ref5] Kim S. A.; Kim B. R.; Chun M. Y.; Youn S. W. Relation between Ph in the Trunk and Face: Truncal Ph Can Be Easily Predicted from Facial Ph. Ann. Dermatol. 2016, 28, 216–221. 10.5021/ad.2016.28.2.216. 27081270 PMC4828386

[ref6] Herath M.; Hosie S.; Bornstein J. C.; Franks A. E.; Hill-Yardin E. L. The Role of the Gastrointestinal Mucus System in Intestinal Homeostasis: Implications for Neurological Disorders. Front. Cell. Infect. Microbiol. 2020, 10, 248 10.3389/fcimb.2020.00248. 32547962 PMC7270209

[ref7] Yatsunenko T.; Rey F. E.; Manary M. J.; Trehan I.; Dominguez-Bello M. G.; Contreras M.; Magris M.; Hidalgo G.; Baldassano R. N.; Anokhin A. P.; et al. Human Gut Microbiome Viewed across Age and Geography. Nature 2012, 486, 222–227. 10.1038/nature11053. 22699611 PMC3376388

[ref8] Guigoz Y.; Dore J.; Schiffrin E. J. The Inflammatory Status of Old Age Can Be Nurtured from the Intestinal Environment. Curr. Opin. Clin. Nutr. Metab. Care 2008, 11, 13–20. 10.1097/MCO.0b013e3282f2bfdf. 18090652

[ref9] Aas J. A.; Paster B. J.; Stokes L. N.; Olsen I.; Dewhirst F. E. Defining the Normal Bacterial Flora of the Oral Cavity. J. Clin. Microbiol. 2005, 43, 5721–5732. 10.1128/JCM.43.11.5721-5732.2005. 16272510 PMC1287824

[ref10] Wade W. G. The Oral Microbiome in Health and Disease. Pharmacol. Res. 2013, 69, 137–143. 10.1016/j.phrs.2012.11.006. 23201354

[ref11] Kumaraswamy K. L.; Vidhya M. Human Papilloma Virus and Oral Infections: An Update. J. Cancer. Res. Ther. 2011, 7, 120–127. 10.4103/0973-1482.82915. 21768696

[ref12] Wantland W. W.; Wantland E. M.; Remo J. W.; Winquist D. L. Studies on Human Mouth Protozoa. J. Dent. Res. 1958, 37, 949–950. 10.1177/00220345580370052601. 13587822

[ref13] Wescombe P. A.; Heng N. C.; Burton J. P.; Chilcott C. N.; Tagg J. R. Streptococcal Bacteriocins and the Case for Streptococcus Salivarius as Model Oral Probiotics. Future Microbiol. 2009, 4, 819–835. 10.2217/fmb.09.61. 19722837

[ref14] Loesche W. Dental Caries and Periodontitis: Contrasting Two Infections That Have Medical Implications. Infect. Dis. Clin. North. Am. 2007, 21, 471–502. 10.1016/j.idc.2007.03.006. 17561079

[ref15] Parahitiyawa N. B.; Scully C.; Leung W. K.; Yam W. C.; Jin L. J.; Samaranayake L. P. Exploring the Oral Bacterial Flora: Current Status and Future Directions. Oral Dis. 2010, 16, 136–145. 10.1111/j.1601-0825.2009.01607.x. 19627515

[ref16] Fejerskov O. Changing Paradigms in Concepts on Dental Caries: Consequences for Oral Health Care. Caries Res. 2004, 38, 182–191. 10.1159/000077753. 15153687

[ref17] Filoche S.; Wong L.; Sissons C. H. Oral Biofilms: Emerging Concepts in Microbial Ecology. J. Dent. Res. 2010, 89, 8–18. 10.1177/0022034509351812. 19918089

[ref18] Zarco M. F.; Vess T. J.; Ginsburg G. S. The Oral Microbiome in Health and Disease and the Potential Impact on Personalized Dental Medicine. Oral Dis. 2012, 18, 109–120. 10.1111/j.1601-0825.2011.01851.x. 21902769

[ref19] Horz H. P.; Conrads G. Diagnosis and Anti-Infective Therapy of Periodontitis. Expert Rev. Anti. Infect. Ther. 2007, 5, 703–715. 10.1586/14787210.5.4.703. 17678431

[ref20] Williams R. C.; Barnett A. H.; Claffey N.; Davis M.; Gadsby R.; Kellett M.; Lip G. Y.; Thackray S. The Potential Impact of Periodontal Disease on General Health: A Consensus View. Curr. Med. Res. Opin. 2008, 24, 1635–1643. 10.1185/03007990802131215. 18452645

[ref21] Li X.; Kolltveit K. M.; Tronstad L.; Olsen I. Systemic Diseases Caused by Oral Infection. Clin. Microbiol. Rev. 2000, 13, 547–558. 10.1128/CMR.13.4.547. 11023956 PMC88948

[ref22] Vieira A. T.; Castelo P. M.; Ribeiro D. A.; Ferreira C. M. Influence of Oral and Gut Microbiota in the Health of Menopausal Women. Front. Microbiol. 2017, 8, 1884 10.3389/fmicb.2017.01884. 29033921 PMC5625026

[ref23] Do L. G.; Ha D. H.; Bell L. K.; Devenish G.; Golley R. K.; Leary S. D.; Manton D. J.; Thomson W. M.; Scott J. A.; Spencer A. J. Study of Mothers’ and Infants’ Life Events Affecting Oral Health (Smile) Birth Cohort Study: Cohort Profile. BMJ. Open 2020, 10, e041185 10.1136/bmjopen-2020-041185. PMC7590353 33099500

[ref24] Craig S. J. C.; Blankenberg D.; Parodi A. C. L.; Paul I. M.; Birch L. L.; Savage J. S.; Marini M. E.; Stokes J. L.; Nekrutenko A.; Reimherr M.; et al. Child Weight Gain Trajectories Linked to Oral Microbiota Composition. Sci. Rep. 2018, 8, 14030 10.1038/s41598-018-31866-9. 30232389 PMC6145887

[ref25] Jensen E. D.; Selway C. A.; Allen G.; Bednarz J.; Weyrich L. S.; Gue S.; Pena A. S.; Couper J. Early Markers of Periodontal Disease and Altered Oral Microbiota Are Associated with Glycemic Control in Children with Type 1 Diabetes. Pediatr. Diabetes 2021, 22, 474–481. 10.1111/pedi.13170. 33398933

[ref26] Willis J. R.; Gabaldon T. The Human Oral Microbiome in Health and Disease: From Sequences to Ecosystems. Microorganisms 2020, 8, 308 10.3390/microorganisms8020308. 32102216 PMC7074908

[ref27] Xue L.; Zou X.; Yang X. Q.; Peng F.; Yu D. K.; Du J. R. Chronic Periodontitis Induces Microbiota-Gut-Brain Axis Disorders and Cognitive Impairment in Mice. Exp. Neurol. 2020, 326, 113176 10.1016/j.expneurol.2020.113176. 31926167

[ref28] Lin D.; Hutchison K. E.; Portillo S.; Vegara V.; Ellingson J. M.; Liu J.; Krauter K. S.; Carroll-Portillo A.; Calhoun V. D. Association between the Oral Microbiome and Brain Resting State Connectivity in Smokers. Neuroimage 2019, 200, 121–131. 10.1016/j.neuroimage.2019.06.023. 31201984 PMC6849507

[ref29] Yang I.; Arthur R. A.; Zhao L.; Clark J.; Hu Y.; Corwin E. J.; Lah J. The Oral Microbiome and Inflammation in Mild Cognitive Impairment. Exp. Gerontol. 2021, 147, 111273 10.1016/j.exger.2021.111273. 33556534

[ref30] Cunha F. A.; Cota L. O. M.; Cortelli S. C.; Miranda T. B.; Neves F. S.; Cortelli J. R.; Costa F. O. Periodontal Condition and Levels of Bacteria Associated with Periodontitis in Individuals with Bipolar Affective Disorders: A Case-Control Study. J. Periodontal Res. 2019, 54, 63–72. 10.1111/jre.12605. 30207388

[ref31] Shafquat A.; Joice R.; Simmons S. L.; Huttenhower C. Functional and Phylogenetic Assembly of Microbial Communities in the Human Microbiome. Trends Microbiol. 2014, 22, 261–266. 10.1016/j.tim.2014.01.011. 24618403 PMC4008634

[ref32] Baker J. L.; Bor B.; Agnello M.; Shi W.; He X. Ecology of the Oral Microbiome: Beyond Bacteria. Trends Microbiol. 2017, 25, 362–374. 10.1016/j.tim.2016.12.012. 28089325 PMC5687246

[ref33] Knight R.; Callewaert C.; Marotz C.; Hyde E. R.; Debelius J. W.; McDonald D.; Sogin M. L. The Microbiome and Human Biology. Annu. Rev. Genomics Hum. Genet. 2017, 18, 65–86. 10.1146/annurev-genom-083115-022438. 28375652

[ref34] Yang I.; Nell S.; Suerbaum S. Survival in Hostile Territory: The Microbiota of the Stomach. FEMS Microbiol. Rev. 2013, 37, 736–761. 10.1111/1574-6976.12027. 23790154

[ref35] Wu W. M.; Yang Y. S.; Peng L. H. Microbiota in the Stomach: New Insights. J. Dig. Dis. 2014, 15, 54–61. 10.1111/1751-2980.12116. 24245792

[ref36] Oren A.; Garrity G. M. Valid Publication of the Names of Forty-Two Phyla of Prokaryotes. Int. J. Syst. Evol. Microbiol. 2021, 71, 005056 10.1099/ijsem.0.005056. 34694987

[ref37] Bik E. M.; Eckburg P. B.; Gill S. R.; Nelson K. E.; Purdom E. A.; Francois F.; Perez-Perez G.; Blaser M. J.; Relman D. A. Molecular Analysis of the Bacterial Microbiota in the Human Stomach. Proc. Natl. Acad. Sci. U. S. A. 2006, 103, 732–737. 10.1073/pnas.0506655103. 16407106 PMC1334644

[ref38] Delgado S.; Cabrera-Rubio R.; Mira A.; Suarez A.; Mayo B. Microbiological Survey of the Human Gastric Ecosystem Using Culturing and Pyrosequencing Methods. Microb. Ecol. 2013, 65, 763–772. 10.1007/s00248-013-0192-5. 23397369

[ref39] Liu X.; Shao L.; Liu X.; Ji F.; Mei Y.; Cheng Y.; Liu F.; Yan C.; Li L.; Ling Z. Alterations of Gastric Mucosal Microbiota across Different Stomach Microhabitats in a Cohort of 276 Patients with Gastric Cancer. EBioMedicine 2019, 40, 336–348. 10.1016/j.ebiom.2018.12.034. 30584008 PMC6412016

[ref40] Nardone G.; Compare D. The Human Gastric Microbiota: Is It Time to Rethink the Pathogenesis of Stomach Diseases?. United European Gastroenterol. J. 2015, 3, 255–260. 10.1177/2050640614566846. PMC4480535 26137299

[ref41] Sung J.; Kim N.; Kim J.; Jo H. J.; Park J. H.; Nam R. H.; Seok Y. J.; Kim Y. R.; Lee D. H.; Jung H. C. Comparison of Gastric Microbiota between Gastric Juice and Mucosa by Next Generation Sequencing Method. J. Cancer. Prev. 2016, 21, 60–65. 10.15430/JCP.2016.21.1.60. 27051651 PMC4819668

[ref42] Zilberstein B.; Quintanilha A. G.; Santos M. A. A.; Pajecki D.; Moura E. G.; Alves P. R A.; Filho F. M.; de Souza J. A. U.; Gama-Rodrigues J. Digestive Tract Microbiota in Healthy Volunteers. Clinics 2007, 62, 47–56. 10.1590/S1807-59322007000100008. 17334549

[ref43] Yu G.; Torres J.; Hu N.; Medrano-Guzman R.; Herrera-Goepfert R.; Humphrys M. S.; Wang L.; Wang C.; Ding T.; Ravel J.; et al. Molecular Characterization of the Human Stomach Microbiota in Gastric Cancer Patients. Front. Cell Infect. Microbiol. 2017, 7, 302 10.3389/fcimb.2017.00302. 28730144 PMC5498480

[ref44] Mitchell D. R.; Derakhshan M. H.; Wirz A. A.; Orange C.; Ballantyne S. A.; Going J. J.; McColl K. E. L. The Gastric Acid Pocket Is Attenuated in H. Pylori Infected Subjects. Gut 2017, 66, 1555–1562. 10.1136/gutjnl-2016-312638. 27663505

[ref45] Li T. H.; Qin Y.; Sham P. C.; Lau K. S.; Chu K. M.; Leung W. K. Alterations in Gastric Microbiota after H. Pylori Eradication and in Different Histological Stages of Gastric Carcinogenesis. Sci. Rep. 2017, 7, 44935 10.1038/srep44935. 28322295 PMC5359573

[ref46] Arumugam M.; Raes J.; Pelletier E.; Le Paslier D.; Yamada T.; Mende D. R.; Fernandes G. R.; Tap J.; Bruls T.; Batto J. M.; et al. Enterotypes of the Human Gut Microbiome. Nature 2011, 473, 174–180. 10.1038/nature09944. 21508958 PMC3728647

[ref47] Eggesbo M.; Moen B.; Peddada S.; Baird D.; Rugtveit J.; Midtvedt T.; Bushel P. R.; Sekelja M.; Rudi K. Development of Gut Microbiota in Infants Not Exposed to Medical Interventions. APMIS 2011, 119, 17–35. 10.1111/j.1600-0463.2010.02688.x. 21143523 PMC3058492

[ref48] Karlsson C. L.; Molin G.; Cilio C. M.; Ahrne S. The Pioneer Gut Microbiota in Human Neonates Vaginally Born at Term-a Pilot Study. Pediatr. Res. 2011, 70, 282–286. 10.1203/PDR.0b013e318225f765. 21629156

[ref49] Biasucci G.; Rubini M.; Riboni S.; Morelli L.; Bessi E.; Retetangos C. Mode of Delivery Affects the Bacterial Community in the Newborn Gut. Early Hum. Dev. 2010, 86, 13–15. 10.1016/j.earlhumdev.2010.01.004. 20133091

[ref50] Huurre A.; Kalliomaki M.; Rautava S.; Rinne M.; Salminen S.; Isolauri E. Mode of Delivery - Effects on Gut Microbiota and Humoral Immunity. Neonatology 2008, 93, 236–240. 10.1159/000111102. 18025796

[ref51] Fallani M.; Young D.; Scott J.; Norin E.; Amarri S.; Adam R.; Aguilera M.; Khanna S.; Gil A.; Edwards C. A.; et al. Intestinal Microbiota of 6-Week-Old Infants across Europe: Geographic Influence Beyond Delivery Mode, Breast-Feeding, and Antibiotics. J. Pediatr. Gastroenterol. Nutr. 2010, 51, 77–84. 10.1097/MPG.0b013e3181d1b11e. 20479681

[ref52] Klaassens E. S.; Boesten R. J.; Haarman M.; Knol J.; Schuren F. H.; Vaughan E. E.; de Vos W. M. Mixed-Species Genomic Microarray Analysis of Fecal Samples Reveals Differential Transcriptional Responses of Bifidobacteria in Breast- and Formula-Fed Infants. Appl. Environ. Microbiol. 2009, 75, 2668–2676. 10.1128/AEM.02492-08. 19286790 PMC2681671

[ref53] Harmsen H. J.; Wildeboer-Veloo A. C.; Raangs G. C.; Wagendorp A. A.; Klijn N.; Bindels J. G.; Welling G. W. Analysis of Intestinal Flora Development in Breast-Fed and Formula-Fed Infants by Using Molecular Identification and Detection Methods. J. Pediatr. Gastroenterol. Nutr. 2000, 30, 61–67. 10.1097/00005176-200001000-00019. 10630441

[ref54] Roger L. C.; McCartney A. L. Longitudinal Investigation of the Faecal Microbiota of Healthy Full-Term Infants Using Fluorescence in Situ Hybridization and Denaturing Gradient Gel Electrophoresis. Microbiology 2010, 156, 3317–3328. 10.1099/mic.0.041913-0. 20829292

[ref55] Favier C. F.; Vaughan E. E.; De Vos W. M.; Akkermans A. D. Molecular Monitoring of Succession of Bacterial Communities in Human Neonates. Appl. Environ. Microbiol. 2002, 68, 219–226. 10.1128/AEM.68.1.219-226.2002. 11772630 PMC126580

[ref56] Fallani M.; Amarri S.; Uusijarvi A.; Adam R.; Khanna S.; Aguilera M.; Gil A.; Vieites J. M.; Norin E.; Young D.; et al. Determinants of the Human Infant Intestinal Microbiota after the Introduction of First Complementary Foods in Infant Samples from Five European Centres. Microbiology 2011, 157, 1385–1392. 10.1099/mic.0.042143-0. 21330436

[ref57] O’Toole P. W.; Claesson M. J. Gut Microbiota: Changes Throughout the Lifespan from Infancy to Elderly. Int. Dairy J. 2010, 20, 281–291. 10.1016/j.idairyj.2009.11.010.

[ref58] Woodmansey E. J. Intestinal Bacteria and Ageing. J. Appl. Microbiol. 2007, 102, 1178–1186. 10.1111/j.1365-2672.2007.03400.x. 17448153

[ref59] Claesson M. J.; Cusack S.; O’Sullivan O.; Greene-Diniz R.; de Weerd H.; Flannery E.; Marchesi J. R.; Falush D.; Dinan T.; Fitzgerald G.; et al. Composition, Variability, and Temporal Stability of the Intestinal Microbiota of the Elderly. Proc. Natl. Acad. Sci. U. S. A. 2011, 108, 4586–4591. 10.1073/pnas.1000097107. 20571116 PMC3063589

[ref60] Flint H. J.; Scott K. P.; Louis P.; Duncan S. H. The Role of the Gut Microbiota in Nutrition and Health. Nat. Rev. Gastroenterol. Hepatol. 2012, 9, 577–589. 10.1038/nrgastro.2012.156. 22945443

[ref61] Islam K. B.; Fukiya S.; Hagio M.; Fujii N.; Ishizuka S.; Ooka T.; Ogura Y.; Hayashi T.; Yokota A. Bile Acid Is a Host Factor That Regulates the Composition of the Cecal Microbiota in Rats. Gastroenterology 2011, 141, 1773–1781. 10.1053/j.gastro.2011.07.046. 21839040

[ref62] Booijink C. C.; El-Aidy S.; Rajilic-Stojanovic M.; Heilig H. G.; Troost F. J.; Smidt H.; Kleerebezem M.; De Vos W. M.; Zoetendal E. G. High Temporal and Inter-Individual Variation Detected in the Human Ileal Microbiota. Environ. Microbiol. 2010, 12, 3213–3227. 10.1111/j.1462-2920.2010.02294.x. 20626454

[ref63] Zoetendal E. G.; Raes J.; van den Bogert B.; Arumugam M.; Booijink C. C.; Troost F. J.; Bork P.; Wels M.; de Vos W. M.; Kleerebezem M. The Human Small Intestinal Microbiota Is Driven by Rapid Uptake and Conversion of Simple Carbohydrates. ISME J. 2012, 6, 1415–1426. 10.1038/ismej.2011.212. 22258098 PMC3379644

[ref64] Rinninella E.; Raoul P.; Cintoni M.; Franceschi F.; Miggiano G. A. D.; Gasbarrini A.; Mele M. C. What Is the Healthy Gut Microbiota Composition? A Changing Ecosystem across Age, Environment, Diet, and Diseases. Microorganisms 2019, 7, 14 10.3390/microorganisms7010014. 30634578 PMC6351938

[ref65] Gill S. R.; Pop M.; Deboy R. T.; Eckburg P. B.; Turnbaugh P. J.; Samuel B. S.; Gordon J. I.; Relman D. A.; Fraser-Liggett C. M.; Nelson K. E. Metagenomic Analysis of the Human Distal Gut Microbiome. Science 2006, 312, 1355–1359. 10.1126/science.1124234. 16741115 PMC3027896

[ref66] Khosravi A.; Mazmanian S. K. Disruption of the Gut Microbiome as a Risk Factor for Microbial Infections. Curr. Opin. Microbiol. 2013, 16, 221–227. 10.1016/j.mib.2013.03.009. 23597788 PMC5695238

[ref67] Walker A. W.; Duncan S. H.; McWilliam Leitch E. C.; Child M. W.; Flint H. J. Ph and Peptide Supply Can Radically Alter Bacterial Populations and Short-Chain Fatty Acid Ratios within Microbial Communities from the Human Colon. Appl. Environ. Microbiol. 2005, 71, 3692–3700. 10.1128/AEM.71.7.3692-3700.2005. 16000778 PMC1169066

[ref68] Louis P.; Scott K. P.; Duncan S. H.; Flint H. J. Understanding the Effects of Diet on Bacterial Metabolism in the Large Intestine. J. Appl. Microbiol. 2007, 102, 1197–1208. 10.1111/j.1365-2672.2007.03322.x. 17448155

[ref69] Kho Z. Y.; Lal S. K. The Human Gut Microbiome - a Potential Controller of Wellness and Disease. Front. Microbiol. 2018, 9, 1835 10.3389/fmicb.2018.01835. 30154767 PMC6102370

[ref70] Barnaba V.; Sinigaglia F. Molecular Mimicry and T Cell-Mediated Autoimmune Disease. J. Exp. Med. 1997, 185, 1529–1531. 10.1084/jem.185.9.1529. 9151889 PMC2196307

[ref71] Bartlett J. G. Antimicrobial Agents Implicated in Clostridium Difficile Toxin-Associated Diarrhea of Colitis. Johns Hopkins Med. J. 1981, 149, 6–9. 7253364

[ref72] Song H. J.; Shim K. N.; Jung S. A.; Choi H. J.; Lee M. A.; Ryu K. H.; Kim S. E.; Yoo K. Antibiotic-Associated Diarrhea: Candidate Organisms Other Than Clostridium Difficile. Korean J. Int. Med. 2008, 23, 9–15. 10.3904/kjim.2008.23.1.9. PMC2686956 18363274

[ref73] Pear S. M.; Williamson T. H.; Bettin K. M.; Gerding D. N.; Galgiani J. N. Decrease in Nosocomial Clostridium Difficile-Associated Diarrhea by Restricting Clindamycin Use. Ann. Int. Med. 1994, 120, 272–277. 10.7326/0003-4819-120-4-199402150-00003. 8080497

[ref74] Lennard-Jones J. E. Classification of Inflammatory Bowel Disease. Scand. J. Gastroenterol. 1989, 24, 2–6. 10.3109/00365528909091339. 2617184

[ref75] Nagao-Kitamoto H.; Shreiner A. B.; Gillilland M. G. 3rd; Kitamoto S.; Ishii C.; Hirayama A.; Kuffa P.; El-Zaatari M.; Grasberger H.; Seekatz A. M.; et al. Functional Characterization of Inflammatory Bowel Disease-Associated Gut Dysbiosis in Gnotobiotic Mice.. Cell Mol. Gastroenterol. Hepatol. 2016, 2, 468–481. 10.1016/j.jcmgh.2016.02.003. 27795980 PMC5042563

[ref76] Sokol H.; Pigneur B.; Watterlot L.; Lakhdari O.; Bermudez-Humaran L. G.; Gratadoux J. J.; Blugeon S.; Bridonneau C.; Furet J. P.; Corthier G.; et al. Faecalibacterium Prausnitzii Is an Anti-Inflammatory Commensal Bacterium Identified by Gut Microbiota Analysis of Crohn Disease Patients. Proc. Natl. Acad. Sci. U. S. A. 2008, 105, 16731–16736. 10.1073/pnas.0804812105. 18936492 PMC2575488

[ref77] Willing B. P.; Dicksved J.; Halfvarson J.; Andersson A. F.; Lucio M.; Zheng Z.; Jarnerot G.; Tysk C.; Jansson J. K.; Engstrand L. A Pyrosequencing Study in Twins Shows That Gastrointestinal Microbial Profiles Vary with Inflammatory Bowel Disease Phenotypes. Gastroenterology 2010, 139, 1844–1854. 10.1053/j.gastro.2010.08.049. 20816835

[ref78] Machiels K.; Joossens M.; Sabino J.; De Preter V.; Arijs I.; Eeckhaut V.; Ballet V.; Claes K.; Van Immerseel F.; Verbeke K.; et al. A Decrease of the Butyrate-Producing Species Roseburia Hominis and Faecalibacterium Prausnitzii Defines Dysbiosis in Patients with Ulcerative Colitis. Gut 2014, 63, 1275–1283. 10.1136/gutjnl-2013-304833. 24021287

[ref79] Peng L.; He Z.; Chen W.; Holzman I. R.; Lin J. Effects of Butyrate on Intestinal Barrier Function in a Caco-2 Cell Monolayer Model of Intestinal Barrier. Pediatr. Res. 2007, 61, 37–41. 10.1203/01.pdr.0000250014.92242.f3. 17211138

[ref80] Carroll I. M.; Chang Y. H.; Park J.; Sartor R. B.; Ringel Y. Luminal and Mucosal-Associated Intestinal Microbiota in Patients with Diarrhea-Predominant Irritable Bowel Syndrome. Gut Pathog. 2010, 2, 19 10.1186/1757-4749-2-19. 21143915 PMC3018384

[ref81] Bhattarai Y.; Muniz Pedrogo D. A.; Kashyap P. C. Irritable Bowel Syndrome: A Gut Microbiota-Related Disorder?. Am. J. Physiol. Gastrointest. Liver Physiol. 2017, 312, G52–G62. 10.1152/ajpgi.00338.2016. 27881403 PMC5283907

[ref82] Salonen A.; de Vos W. M.; Palva A. Gastrointestinal Microbiota in Irritable Bowel Syndrome: Present State and Perspectives. Microbiology 2010, 156, 3205–3215. 10.1099/mic.0.043257-0. 20705664

[ref83] Greenblum S.; Turnbaugh P. J.; Borenstein E. Metagenomic Systems Biology of the Human Gut Microbiome Reveals Topological Shifts Associated with Obesity and Inflammatory Bowel Disease. Proc. Natl. Acad. Sci. U. S. A. 2012, 109, 594–599. 10.1073/pnas.1116053109. 22184244 PMC3258644

[ref84] Schloissnig S.; Arumugam M.; Sunagawa S.; Mitreva M.; Tap J.; Zhu A.; Waller A.; Mende D. R.; Kultima J. R.; Martin J.; et al. Genomic Variation Landscape of the Human Gut Microbiome. Nature 2013, 493, 45–50. 10.1038/nature11711. 23222524 PMC3536929

[ref85] Faith J. J.; Guruge J. L.; Charbonneau M.; Subramanian S.; Seedorf H.; Goodman A. L.; Clemente J. C.; Knight R.; Heath A. C.; Leibel R. L.; et al. The Long-Term Stability of the Human Gut Microbiota. Science 2013, 341, 1237439 10.1126/science.1237439. 23828941 PMC3791589

[ref86] Yan M.; Pamp S. J.; Fukuyama J.; Hwang P. H.; Cho D. Y.; Holmes S.; Relman D. A. Nasal Microenvironments and Interspecific Interactions Influence Nasal Microbiota Complexity and S. Aureus Carriage. Cell Host Microbe 2013, 14, 631–640. 10.1016/j.chom.2013.11.005. 24331461 PMC3902146

[ref87] Biswas K.; Hoggard M.; Jain R.; Taylor M. W.; Douglas R. G. The Nasal Microbiota in Health and Disease: Variation within and between Subjects. Front. Microbiol. 2015, 9, 134 10.3389/fmicb.2015.00134. 25784909 PMC4345913

[ref88] Bassis C. M.; Tang A. L.; Young V. B.; Pynnonen M. A. The Nasal Cavity Microbiota of Healthy Adults. Microbiome 2014, 2, 27 10.1186/2049-2618-2-27. 25143824 PMC4138944

[ref89] Zhou Y.; Mihindukulasuriya K. A.; Gao H.; La Rosa P. S.; Wylie K. M.; Martin J. C.; Kota K.; Shannon W. D.; Mitreva M.; Sodergren E.; et al. Exploration of Bacterial Community Classes in Major Human Habitats. Genome Biol. 2014, 15, R66 10.1186/gb-2014-15-5-r66. 24887286 PMC4073010

[ref90] Ramakrishnan V. R.; Feazel L. M.; Gitomer S. A.; Ir D.; Robertson C. E.; Frank D. N. The Microbiome of the Middle Meatus in Healthy Adults. PLoS One 2013, 8, e85507 10.1371/journal.pone.0085507. 24386477 PMC3875580

[ref91] Zhang Z.; Adappa N. D.; Doghramji L. J.; Chiu A. G.; Cohen N. A.; Palmer J. N. Different Clinical Factors Associated with Staphylococcus Aureus and Pseudomonas Aeruginosa in Chronic Rhinosinusitis. Int. Forum Allergy. Rhinol. 2015, 5, 724–733. 10.1002/alr.21532. 25899601

[ref92] Abreu N. A.; Nagalingam N. A.; Song Y.; Roediger F. C.; Pletcher S. D.; Goldberg A. N.; Lynch S. V. Sinus Microbiome Diversity Depletion and Corynebacterium Tuberculostearicum Enrichment Mediates Rhinosinusitis. Sci. Transl. Med. 2012, 4, 151ra124 10.1126/scitranslmed.3003783. PMC4786373 22972842

[ref93] Huang Y. J.; Nelson C. E.; Brodie E. L.; DeSantis T. Z.; Baek M. S.; Liu J.; Woyke T.; Allgaier M.; Bristow J.; Wiener-Kronish J. P.; et al. Airway Microbiota and Bronchial Hyperresponsiveness in Patients with Suboptimally Controlled Asthma. J. Allergy Clin. Immunol. 2011, 127, 372–381. 10.1016/j.jaci.2010.10.048. 21194740 PMC3037020

[ref94] Marri P. R.; Stern D. A.; Wright A. L.; Billheimer D.; Martinez F. D. Asthma-Associated Differences in Microbial Composition of Induced Sputum. J. Allergy Clin. Immunol. 2013, 131, 346–352. 10.1016/j.jaci.2012.11.013. 23265859 PMC4403876

[ref95] Bisgaard H.; Hermansen M. N.; Buchvald F.; Loland L.; Halkjaer L. B.; Bonnelykke K.; Brasholt M.; Heltberg A.; Vissing N. H.; Thorsen S. V.; et al. Childhood Asthma after Bacterial Colonization of the Airway in Neonates. N. Engl. J. Med. 2007, 357, 1487–1495. 10.1056/NEJMoa052632. 17928596

[ref96] Kraft M. The Role of Bacterial Infections in Asthma. Clin. Chest. Med. 2000, 21, 301–313. 10.1016/S0272-5231(05)70268-9. 10907590

[ref97] Sethi S.; Evans N.; Grant B. J.; Murphy T. F. New Strains of Bacteria and Exacerbations of Chronic Obstructive Pulmonary Disease. N. Engl. J. Med. 2002, 347, 465–471. 10.1056/NEJMoa012561. 12181400

[ref98] Dickson R. P.; Erb-Downward J. R.; Martinez F. J.; Huffnagle G. B. The Microbiome and the Respiratory Tract. Annu. Rev. Physiol. 2016, 78, 481–504. 10.1146/annurev-physiol-021115-105238. 26527186 PMC4751994

[ref99] Bassis C. M.; Erb-Downward J. R.; Dickson R. P.; Freeman C. M.; Schmidt T. M.; Young V. B.; Beck J. M.; Curtis J. L.; Huffnagle G. B. Analysis of the Upper Respiratory Tract Microbiotas as the Source of the Lung and Gastric Microbiotas in Healthy Individuals. mBio 2015, 6, e00037-15 10.1128/mBio.00037-15. 25736890 PMC4358017

[ref100] Hanada S.; Pirzadeh M.; Carver K. Y.; Deng J. C. Respiratory Viral Infection-Induced Microbiome Alterations and Secondary Bacterial Pneumonia. Front. Immunol. 2018, 9, 2640 10.3389/fimmu.2018.02640. 30505304 PMC6250824

[ref101] Soret P.; Vandenborght L. E.; Francis F.; Coron N.; Enaud R.; Avalos M.; Schaeverbeke T.; Berger P.; Fayon M.; Thiebaut R.; et al. Respiratory Mycobiome and Suggestion of Inter-Kingdom Network During Acute Pulmonary Exacerbation in Cystic Fibrosis. Sci. Rep. 2020, 10, 3589 10.1038/s41598-020-60015-4. 32108159 PMC7046743

[ref102] Worlitzsch D.; Tarran R.; Ulrich M.; Schwab U.; Cekici A.; Meyer K. C.; Birrer P.; Bellon G.; Berger J.; Weiss T.; et al. Effects of Reduced Mucus Oxygen Concentration in Airway Pseudomonas Infections of Cystic Fibrosis Patients. J. Clin. Invest. 2002, 109, 317–325. 10.1172/JCI0213870. 11827991 PMC150856

[ref103] Schmidt A.; Belaaouaj A.; Bissinger R.; Koller G.; Malleret L.; D’Orazio C.; Facchinelli M.; Schulte-Hubbert B.; Molinaro A.; Holst O.; et al. Neutrophil Elastase-Mediated Increase in Airway Temperature During Inflammation. J. Cyst. Fibros. 2014, 13, 623–631. 10.1016/j.jcf.2014.03.004. 24713593

[ref104] Casadevall A.; Pirofski L. A. The Damage-Response Framework of Microbial Pathogenesis. Nat. Rev. Microbiol. 2003, 1, 17–24. 10.1038/nrmicro732. 15040176 PMC7097162

[ref105] Freestone P. P.; Hirst R. A.; Sandrini S. M.; Sharaff F.; Fry H.; Hyman S.; O’Callaghan C. Pseudomonas Aeruginosa-Catecholamine Inotrope Interactions: A Contributory Factor in the Development of Ventilator-Associated Pneumonia?. Chest 2012, 142, 1200–1210. 10.1378/chest.11-2614. 22556319

[ref106] Kanangat S.; Meduri G. U.; Tolley E. A.; Patterson D. R.; Meduri C. U.; Pak C.; Griffin J. P.; Bronze M. S.; Schaberg D. R. Effects of Cytokines and Endotoxin on the Intracellular Growth of Bacteria. Infect. Immun. 1999, 67, 2834–2840. 10.1128/IAI.67.6.2834-2840.1999. 10338488 PMC96589

[ref107] Kaza S. K.; McClean S.; Callaghan M. Il-8 Released from Human Lung Epithelial Cells Induced by Cystic Fibrosis Pathogens Burkholderia Cepacia Complex Affects the Growth and Intracellular Survival of Bacteria. Int. J. Med. Microbiol. 2011, 301, 26–33. 10.1016/j.ijmm.2010.06.005. 20829108

[ref108] Lyte M.; Ernst S. Catecholamine Induced Growth of Gram Negative Bacteria. Life Sci. 1992, 50, 203–212. 10.1016/0024-3205(92)90273-R. 1731173

[ref109] Dickson R. P.; Martinez F. J.; Huffnagle G. B. The Role of the Microbiome in Exacerbations of Chronic Lung Diseases. Lancet 2014, 384, 691–702. 10.1016/S0140-6736(14)61136-3. 25152271 PMC4166502

[ref110] Byrd A. L.; Belkaid Y.; Segre J. A. The Human Skin Microbiome. Nat. Rev. Microbiol. 2018, 16, 143–155. 10.1038/nrmicro.2017.157. 29332945

[ref111] Adamczyk K.; Garncarczyk A.; Antonczak P.; Wcislo-Dziadecka D. The Foot Microbiome. J. Cosmet. Dermatol. 2020, 19, 1039–1043. 10.1111/jocd.13368. 32162464

[ref112] Costello E. K.; Lauber C. L.; Hamady M.; Fierer N.; Gordon J. I.; Knight R. Bacterial Community Variation in Human Body Habitats across Space and Time. Science 2009, 326, 1694–1697. 10.1126/science.1177486. 19892944 PMC3602444

[ref113] Grice E. A.; Kong H. H.; Conlan S.; Deming C. B.; Davis J.; Young A. C.; Bouffard G. G.; Blakesley R. W.; Murray P. R.; et al. Topographical and Temporal Diversity of the Human Skin Microbiome. Science 2009, 324, 1190–1192. 10.1126/science.1171700. 19478181 PMC2805064

[ref114] Grice E. A.; Segre J. A. The Skin Microbiome. Nat. Rev. Microbiol. 2011, 9, 244–253. 10.1038/nrmicro2537. 21407241 PMC3535073

[ref115] Oh J.; Byrd A. L.; Deming C.; Conlan S.; Kong H. H.; Segre J. A. Biogeography and Individuality Shape Function in the Human Skin Metagenome. Nature 2014, 514, 59–64. 10.1038/nature13786. 25279917 PMC4185404

[ref116] Findley K.; Oh J.; Yang J.; Conlan S.; Deming C.; Meyer J. A.; Schoenfeld D.; Nomicos E.; Park M.; et al. Topographic Diversity of Fungal and Bacterial Communities in Human Skin. Nature 2013, 498, 367–370. 10.1038/nature12171. 23698366 PMC3711185

[ref117] Oh J.; Byrd A. L.; Park M.; Kong H. H.; Segre J. A. Temporal Stability of the Human Skin Microbiome. Cell 2016, 165, 854–866. 10.1016/j.cell.2016.04.008. 27153496 PMC4860256

[ref118] Feng H.; Shuda M.; Chang Y.; Moore P. S. Clonal Integration of a Polyomavirus in Human Merkel Cell Carcinoma. Science 2008, 319, 1096–1100. 10.1126/science.1152586. 18202256 PMC2740911

[ref119] Hannigan G. D.; Meisel J. S.; Tyldsley A. S.; Zheng Q.; Hodkinson B. P.; SanMiguel A. J.; Minot S.; Bushman F. D.; Grice E. A. The Human Skin Double-Stranded DNA Virome: Topographical and Temporal Diversity, Genetic Enrichment, and Dynamic Associations with the Host Microbiome. mBio 2015, 6, e01578-15 10.1128/mBio.01578-15. 26489866 PMC4620475

[ref120] Scharschmidt T. C.; Fischbach M. A. What Lives on Our Skin: Ecology, Genomics and Therapeutic Opportunities of the Skin Microbiome. Drug Discovery Today Dis. Mech. 2013, 10, e83–e89. 10.1016/j.ddmec.2012.12.003. PMC3833721 24273587

[ref121] Holland K. T.; Greenman J.; Cunliffe W. J. Growth of Cutaneous Propionibacteria on Synthetic Medium; Growth Yields and Exoenzyme Production. J. Appl. Bacteriol. 1979, 47, 383–394. 10.1111/j.1365-2672.1979.tb01198.x. 541303

[ref122] Bruggemann H.; Henne A.; Hoster F.; Liesegang H.; Wiezer A.; Strittmatter A.; Hujer S.; Durre P.; Gottschalk G. The Complete Genome Sequence of Propionibacterium Acnes, a Commensal of Human Skin. Science 2004, 305, 671–673. 10.1126/science.1100330. 15286373

[ref123] Marples R. R.; Downing D. T.; Kligman A. M. Control of Free Fatty Acids in Human Surface Lipids by Corynebacterium Acnes. J. Invest. Dermatol. 1971, 56, 127–131. 10.1111/1523-1747.ep12260695. 4997367

[ref124] Ingham E.; Holland K. T.; Gowland G.; Cunliffe W. J. Partial Purification and Characterization of Lipase (Ec 3.1.1.3) from Propionibacterium Acnes. J. Gen. Microbiol. 1981, 124, 393–401. 7035615 10.1099/00221287-124-2-393

[ref125] Gribbon E. M.; Cunliffe W. J.; Holland K. T. Interaction of Propionibacterium Acnes with Skin Lipids in Vitro. J. Gen. Microbiol. 1993, 139, 1745–1751. 10.1099/00221287-139-8-1745. 8409917

[ref126] Mukherjee S.; Mitra R.; Maitra A.; Gupta S.; Kumaran S.; Chakrabortty A.; Majumder P. P. Sebum and Hydration Levels in Specific Regions of Human Face Significantly Predict the Nature and Diversity of Facial Skin Microbiome. Sci. Rep. 2016, 6, 36062 10.1038/srep36062. 27786295 PMC5081537

[ref127] Oh J.; Conlan S.; Polley E. C.; Segre J. A.; Kong H. H. Shifts in Human Skin and Nares Microbiota of Healthy Children and Adults. Genome Med. 2012, 4, 77 10.1186/gm378. 23050952 PMC3580446

[ref128] Jo J. H.; Deming C.; Kennedy E. A.; Conlan S.; Polley E. C.; Ng W. I.; Segre J. A.; Kong H. H. Diverse Human Skin Fungal Communities in Children Converge in Adulthood. J. Invest. Dermatol. 2016, 136, 2356–2363. 10.1016/j.jid.2016.05.130. 27476723 PMC5687974

[ref129] Jo J. H.; Kennedy E. A.; Kong H. H. Topographical and Physiological Differences of the Skin Mycobiome in Health and Disease. Virulence 2017, 8, 324–333. 10.1080/21505594.2016.1249093. 27754756 PMC5411233

[ref130] Kyriakis K. P.; Terzoudi S.; Palamaras I.; Pagana G.; Michailides C.; Emmanuelides S. Pityriasis Versicolor Prevalence by Age and Gender. Mycoses 2006, 49, 517–518. 10.1111/j.1439-0507.2006.01285.x. 17022772

[ref131] Havlickova B.; Czaika V. A.; Friedrich M. Epidemiological Trends in Skin Mycoses Worldwide. Mycoses 2008, 51, 2–15. 10.1111/j.1439-0507.2008.01606.x. 18783559

[ref132] Seebacher C.; Bouchara J. P.; Mignon B. Updates on the Epidemiology of Dermatophyte Infections. Mycopathologia 2008, 166, 335–352. 10.1007/s11046-008-9100-9. 18478365

[ref133] Tomida S.; Nguyen L.; Chiu B. H.; Liu J.; Sodergren E.; Weinstock G. M.; Li H. Pan-Genome and Comparative Genome Analyses of Propionibacterium Acnes Reveal Its Genomic Diversity in the Healthy and Diseased Human Skin Microbiome. mBio 2013, 4, e00003-13 10.1128/mBio.00003-13. 23631911 PMC3663185

[ref134] Fitz-Gibbon S.; Tomida S.; Chiu B. H.; Nguyen L.; Du C.; Liu M.; Elashoff D.; Erfe M. C.; Loncaric A.; Kim J.; et al. Propionibacterium Acnes Strain Populations in the Human Skin Microbiome Associated with Acne. J. Invest. Dermatol. 2013, 133, 2152–2160. 10.1038/jid.2013.21. 23337890 PMC3745799

[ref135] Kang D.; Shi B.; Erfe M. C.; Craft N.; Li H. Vitamin B12 Modulates the Transcriptome of the Skin Microbiota in Acne Pathogenesis. Sci. Transl. Med. 2015, 7, 293 10.1126/scitranslmed.aab2009. PMC6049814 26109103

[ref136] Picardo M.; Ottaviani M.; Camera E.; Mastrofrancesco A. Sebaceous Gland Lipids. Dermatoendocrinol. 2009, 1, 68–71. 10.4161/derm.1.2.8472. 20224686 PMC2835893

[ref137] Jahns A. C.; Lundskog B.; Ganceviciene R.; Palmer R. H.; Golovleva I.; Zouboulis C. C.; McDowell A.; Patrick S.; Alexeyev O. A. An Increased Incidence of Propionibacterium Acnes Biofilms in Acne Vulgaris: A Case-Control Study. Br. J. Dermatol. 2012, 167, 50–58. 10.1111/j.1365-2133.2012.10897.x. 22356121

[ref138] Leyden J. J.; Marples R. R.; Kligman A. M. Staphylococcus Aureus in the Lesions of Atopic Dermatitis. Br. J. Dermatol. 1974, 90, 525–530. 10.1111/j.1365-2133.1974.tb06447.x. 4601016

[ref139] Salava A.; Lauerma A. Role of the Skin Microbiome in Atopic Dermatitis. Clin. Transl. Allergy 2014, 4, 33 10.1186/2045-7022-4-33. 25905004 PMC4405870

[ref140] Drago L.; De Grandi R.; Altomare G.; Pigatto P.; Rossi O.; Toscano M. Skin Microbiota of First Cousins Affected by Psoriasis and Atopic Dermatitis. Clin. Mol. Allergy 2016, 14, 2 10.1186/s12948-016-0038-z. 26811697 PMC4724956

[ref141] Gonzalez M. E.; Schaffer J. V.; Orlow S. J.; Gao Z.; Li H.; Alekseyenko A. V.; Blaser M. J. Cutaneous Microbiome Effects of Fluticasone Propionate Cream and Adjunctive Bleach Baths in Childhood Atopic Dermatitis. J. Am. Acad. Dermatol. 2016, 75, 481–493. 10.1016/j.jaad.2016.04.066. 27543211 PMC4992571

[ref142] Zasloff M. Antimicrobial Peptides, Innate Immunity, and the Normally Sterile Urinary Tract. J. Am. Soc. Nephrol. 2007, 18, 2810–2816. 10.1681/ASN.2007050611. 17942949

[ref143] Wolfe A. J.; Brubaker L. “Sterile Urine” and the Presence of Bacteria. Eur. Urol. 2015, 68, 173–174. 10.1016/j.eururo.2015.02.041. 25774008 PMC4659483

[ref144] Pearce M. M.; Hilt E. E.; Rosenfeld A. B.; Zilliox M. J.; Thomas-White K.; Fok C.; Kliethermes S.; Schreckenberger P. C.; Brubaker L.; Gai X.; et al. The Female Urinary Microbiome: A Comparison of Women with and without Urgency Urinary Incontinence. mBio 2014, 5, e01283-14 10.1128/mBio.01283-14. 25006228 PMC4161260

[ref145] Mansour B.; Monyok A.; Makra N.; Gajdacs M.; Vadnay I.; Ligeti B.; Juhasz J.; Szabo D.; Ostorhazi E. Bladder Cancer-Related Microbiota: Examining Differences in Urine and Tissue Samples. Sci. Rep. 2020, 10, 11042 10.1038/s41598-020-67443-2. 32632181 PMC7338485

[ref146] Modena B. D.; Milam R.; Harrison F.; Cheeseman J. A.; Abecassis M. M.; Friedewald J. J.; Kirk A. D.; Salomon D. R. Changes in Urinary Microbiome Populations Correlate in Kidney Transplants with Interstitial Fibrosis and Tubular Atrophy Documented in Early Surveillance Biopsies. Am. J. Transplant. 2017, 17, 712–723. 10.1111/ajt.14038. 27597148 PMC5328852

[ref147] Fouts D. E.; Pieper R.; Szpakowski S.; Pohl H.; Knoblach S.; Suh M. J.; Huang S. T.; Ljungberg I.; Sprague B. M.; Lucas S. K.; et al. Integrated Next-Generation Sequencing of 16s Rdna and Metaproteomics Differentiate the Healthy Urine Microbiome from Asymptomatic Bacteriuria in Neuropathic Bladder Associated with Spinal Cord Injury. J. Transl. Med. 2012, 10, 174 10.1186/1479-5876-10-174. 22929533 PMC3511201

[ref148] Ruiz-Gomez M. L.; Martin-Way D. A.; Perez-Ramirez M. D.; Gutierrez-Fernandez J. Male Deep Infections by Gardnerella Vaginalis. A Literature Review and a Case Report. Rev. Esp. Quimioter. 2019, 32, 469–472. 31515976 PMC6790880

[ref149] Gottschick C.; Deng Z. L.; Vital M.; Masur C.; Abels C.; Pieper D. H.; Wagner-Dobler I. The Urinary Microbiota of Men and Women and Its Changes in Women During Bacterial Vaginosis and Antibiotic Treatment. Microbiome 2017, 5, 99 10.1186/s40168-017-0305-3. 28807017 PMC5554977

[ref150] Bajic P.; Van Kuiken M. E.; Burge B. K.; Kirshenbaum E. J.; Joyce C. J.; Wolfe A. J.; Branch J. D.; Bresler L.; Farooq A. V. Male Bladder Microbiome Relates to Lower Urinary Tract Symptoms. Eur. Urol. Focus 2020, 6, 376–382. 10.1016/j.euf.2018.08.001. 30143471

[ref151] Groah S. L.; Perez-Losada M.; Caldovic L.; Ljungberg I. H.; Sprague B. M.; Castro-Nallar E.; Chandel N. J.; Hsieh M. H.; Pohl H. G. Redefining Healthy Urine: A Cross-Sectional Exploratory Metagenomic Study of People with and without Bladder Dysfunction. J. Urol. 2016, 196, 579–587. 10.1016/j.juro.2016.01.088. 26807926

[ref152] Thomas-White K. J.; Gao X.; Lin H.; Fok C. S.; Ghanayem K.; Mueller E. R.; Dong Q.; Brubaker L.; Wolfe A. J. Urinary Microbes and Postoperative Urinary Tract Infection Risk in Urogynecologic Surgical Patients. Int. Urogynecol. J. 2018, 29, 1797–1805. 10.1007/s00192-018-3767-3. 30267143 PMC6527134

[ref153] Komesu Y. M.; Dinwiddie D. L.; Richter H. E.; Lukacz E. S.; Sung V. W.; Siddiqui N. Y.; Zyczynski H. M.; Ridgeway B.; Rogers R. G.; Arya L. A.; et al. Defining the Relationship between Vaginal and Urinary Microbiomes. Am. J. Obstet. Gynecol. 2020, 222, 154.E1–154.E10. 10.1016/j.ajog.2019.08.011. PMC6995424 31421123

[ref154] Grine G.; Lotte R.; Chirio D.; Chevalier A.; Raoult D.; Drancourt M.; Ruimy R. Co-Culture of Methanobrevibacter Smithii with Enterobacteria During Urinary Infection. EBioMedicine 2019, 43, 333–337. 10.1016/j.ebiom.2019.04.037. 31072770 PMC6558020

[ref155] Johnson G.; Wolfe A. J.; Putonti C. Characterization of the φCTX-Like Pseudomonas Aeruginosa Phage Dobby Isolated from the Kidney Stone Microbiota. Access Microbiol. 2019, 1, e000002 10.1099/acmi.0.000002. 32864566 PMC7454045

[ref156] Malki K.; Sible E.; Cooper A.; Garretto A.; Bruder K.; Watkins S. C.; Putonti C. Seven Bacteriophages Isolated from the Female Urinary Microbiota. Genome Announce. 2016, 4, e01003-16 10.1128/genomeA.01003-16. PMC5122675 27881533

[ref157] Khasriya R.; Sathiananthamoorthy S.; Ismail S.; Kelsey M.; Wilson M.; Rohn J. L.; Malone-Lee J. Spectrum of Bacterial Colonization Associated with Urothelial Cells from Patients with Chronic Lower Urinary Tract Symptoms. J. Clin. Microbiol. 2013, 51, 2054–2062. 10.1128/JCM.03314-12. 23596238 PMC3697662

[ref158] Paalanne N.; Husso A.; Salo J.; Pievilainen O.; Tejesvi M. V.; Koivusaari P.; Pirttila A. M.; Pokka T.; Mattila S.; Jyrkas J.; et al. Intestinal Microbiome as a Risk Factor for Urinary Tract Infections in Children. Eur. J. Clin. Microbiol. Infect. Dis. 2018, 37, 1881–1891. 10.1007/s10096-018-3322-7. 30006660

[ref159] Magruder M.; Sholi A. N.; Gong C.; Zhang L.; Edusei E.; Huang J.; Albakry S.; Satlin M. J.; Westblade L. F.; Crawford C.; et al. Gut Uropathogen Abundance Is a Risk Factor for Development of Bacteriuria and Urinary Tract Infection. Nat. Commun. 2019, 10, 5521 10.1038/s41467-019-13467-w. 31797927 PMC6893017

[ref160] Garretto A.; Miller-Ensminger T.; Ene A.; Merchant Z.; Shah A.; Gerodias A.; Biancofiori A.; Canchola S.; Canchola S.; Castillo E.; et al. Genomic Survey of E. Coli from the Bladders of Women with and without Lower Urinary Tract Symptoms. Front. Microbiol. 2020, 11, 2094 10.3389/fmicb.2020.02094. 33013764 PMC7500147

[ref161] Lavigne J. P.; Nicolas-Chanoine M. H.; Bourg G.; Moreau J.; Sotto A. Virulent Synergistic Effect between Enterococcus Faecalis and Escherichia Coli Assayed by Using the Caenorhabditis Elegans Model. PLoS One 2008, 3, e3370 10.1371/journal.pone.0003370. 18843374 PMC2557124

[ref162] Croxall G.; Weston V.; Joseph S.; Manning G.; Cheetham P.; McNally A. Increased Human Pathogenic Potential of Escherichia Coli from Polymicrobial Urinary Tract Infections in Comparison to Isolates from Monomicrobial Culture Samples. J. Med. Microbiol. 2011, 60, 102–109. 10.1099/jmm.0.020602-0. 20947667

[ref163] Stapleton A. E.; Au-Yeung M.; Hooton T. M.; Fredricks D. N.; Roberts P. L.; Czaja C. A.; Yarova-Yarovaya Y.; Fiedler T.; Cox M.; Stamm W. E. Randomized, Placebo-Controlled Phase 2 Trial of a Lactobacillus Crispatus Probiotic Given Intravaginally for Prevention of Recurrent Urinary Tract Infection. Clin. Infect. Dis. 2011, 52, 1212–1217. 10.1093/cid/cir183. 21498386 PMC3079401

[ref164] Sumati A. H.; Saritha N. K. Association of Urinary Tract Infection in Women with Bacterial Vaginosis. J. Glob. Infect. Dis. 2009, 1, 151–152. 10.4103/0974-777X.56254. 20300409 PMC2840952

[ref165] Gilbert N. M.; O’Brien V. P.; Lewis A. L. Transient Microbiota Exposures Activate Dormant Escherichia Coli Infection in the Bladder and Drive Severe Outcomes of Recurrent Disease. PLoS Pathog. 2017, 13, e1006238 10.1371/journal.ppat.1006238. 28358889 PMC5373645

[ref166] Ravel J.; Gajer P.; Abdo Z.; Schneider G. M.; Koenig S. S.; McCulle S. L.; Karlebach S.; Gorle R.; Russell J.; Tacket C. O.; et al. Vaginal Microbiome of Reproductive-Age Women. Proc. Natl. Acad. Sci. U. S. A. 2011, 108, 4680–4687. 10.1073/pnas.1002611107. 20534435 PMC3063603

[ref167] Antonio M. A.; Hawes S. E.; Hillier S. L. The Identification of Vaginal Lactobacillus Species and the Demographic and Microbiologic Characteristics of Women Colonized by These Species. J. Infect. Dis. 1999, 180, 1950–1956. 10.1086/315109. 10558952

[ref168] Zhou X.; Bent S. J.; Schneider M. G.; Davis C. C.; Islam M. R.; Forney L. J. Characterization of Vaginal Microbial Communities in Adult Healthy Women Using Cultivation-Independent Methods. Microbiology 2004, 150, 2565–2573. 10.1099/mic.0.26905-0. 15289553

[ref169] Boskey E. R.; Telsch K. M.; Whaley K. J.; Moench T. R.; Cone R. A. Acid Production by Vaginal Flora in Vitro Is Consistent with the Rate and Extent of Vaginal Acidification. Infect. Immun. 1999, 67, 5170–5175. 10.1128/IAI.67.10.5170-5175.1999. 10496892 PMC96867

[ref170] O’Hanlon D. E.; Moench T. R.; Cone R. A. Vaginal Ph and Microbicidal Lactic Acid When Lactobacilli Dominate the Microbiota. PLoS One 2013, 8, e80074 10.1371/journal.pone.0080074. 24223212 PMC3819307

[ref171] Aldunate M.; Srbinovski D.; Hearps A. C.; Latham C. F.; Ramsland P. A.; Gugasyan R.; Cone R. A.; Tachedjian G. Antimicrobial and Immune Modulatory Effects of Lactic Acid and Short Chain Fatty Acids Produced by Vaginal Microbiota Associated with Eubiosis and Bacterial Vaginosis. Front. Physiol. 2015, 6, 164 10.3389/fphys.2015.00164. 26082720 PMC4451362

[ref172] Delgado-Diaz D. J.; Tyssen D.; Hayward J. A.; Gugasyan R.; Hearps A. C.; Tachedjian G. Distinct Immune Responses Elicited from Cervicovaginal Epithelial Cells by Lactic Acid and Short Chain Fatty Acids Associated with Optimal and Non-Optimal Vaginal Microbiota. Front. Cell. Infect. Microbiol. 2020, 9, 446 10.3389/fcimb.2019.00446. 31998660 PMC6965070

[ref173] Zhou X.; Brown C. J.; Abdo Z.; Davis C. C.; Hansmann M. A.; Joyce P.; Foster J. A.; Forney L. J. Differences in the Composition of Vaginal Microbial Communities Found in Healthy Caucasian and Black Women. ISME J. 2007, 1, 121–133. 10.1038/ismej.2007.12. 18043622

[ref174] Zhou X.; Hansmann M. A.; Davis C. C.; Suzuki H.; Brown C. J.; Schutte U.; Pierson J. D.; Forney L. J. The Vaginal Bacterial Communities of Japanese Women Resemble Those of Women in Other Racial Groups. FEMS Immunol. Med. Microbiol. 2010, 58, 169–181. 10.1111/j.1574-695X.2009.00618.x. 19912342 PMC2868947

[ref175] Verstraelen H.; Verhelst R.; Claeys G.; Temmerman M.; Vaneechoutte M. Culture-Independent Analysis of Vaginal Microflora: The Unrecognized Association of Atopobium Vaginae with Bacterial Vaginosis. Am. J. Obstet. Gynecol. 2004, 191, 1130–1132. 10.1016/j.ajog.2004.04.013. 15507931

[ref176] Peebles K.; Velloza J.; Balkus J. E.; McClelland R. S.; Barnabas R. V. High Global Burden and Costs of Bacterial Vaginosis: A Systematic Review and Meta-Analysis. Sex Transm. Dis. 2019, 46, 304–311. 10.1097/OLQ.0000000000000972. 30624309

[ref177] Allsworth J. E.; Peipert J. F. Prevalence of Bacterial Vaginosis: 2001–2004 National Health and Nutrition Examination Survey Data. Obstet. Gynecol. 2007, 109, 114–120. 10.1097/01.AOG.0000247627.84791.91. 17197596

[ref178] Brotman R. M.; Klebanoff M. A.; Nansel T. R.; Yu K. F.; Andrews W. W.; Zhang J.; Schwebke J. R. Bacterial Vaginosis Assessed by Gram Stain and Diminished Colonization Resistance to Incident Gonococcal, Chlamydial, and Trichomonal Genital Infection. J. Infect. Dis. 2010, 202, 1907–1915. 10.1086/657320. 21067371 PMC3053135

[ref179] Brotman R. M.; Bradford L. L.; Conrad M.; Gajer P.; Ault K.; Peralta L.; Forney L. J.; Carlton J. M.; Abdo Z.; Ravel J. Association between Trichomonas Vaginalis and Vaginal Bacterial Community Composition among Reproductive-Age Women. Sex Transm. Dis. 2012, 39, 807–812. 10.1097/OLQ.0b013e3182631c79. 23007708 PMC3458234

[ref180] Martin H. L.; Richardson B. A.; Nyange P. M.; Lavreys L.; Hillier S. L.; Chohan B.; Mandaliya K.; Ndinya-Achola J. O.; Bwayo J.; Kreiss J. Vaginal Lactobacilli, Microbial Flora, and Risk of Human Immunodeficiency Virus Type 1 and Sexually Transmitted Disease Acquisition. J. Infect. Dis. 1999, 180, 1863–1868. 10.1086/315127. 10558942

[ref181] Gosmann C.; Anahtar M. N.; Handley S. A.; Farcasanu M.; Abu-Ali G.; Bowman B. A.; Padavattan N.; Desai C.; Droit L.; Moodley A.; et al. Lactobacillus-Deficient Cervicovaginal Bacterial Communities Are Associated with Increased Hiv Acquisition in Young South African Women. Immunity 2017, 46, 29–37. 10.1016/j.immuni.2016.12.013. 28087240 PMC5270628

[ref182] Feehily C.; Crosby D.; Walsh C. J.; Lawton E. M.; Higgins S.; McAuliffe F. M.; Cotter P. D. Shotgun Sequencing of the Vaginal Microbiome Reveals Both a Species and Functional Potential Signature of Preterm Birth. npj Biofilms Microbiomes 2020, 6, 50 10.1038/s41522-020-00162-8. 33184260 PMC7665020

[ref183] Brown R. G.; Al-Memar M.; Marchesi J. R.; Lee Y. S.; Smith A.; Chan D.; Lewis H.; Kindinger L.; Terzidou V.; Bourne T.; et al. Establishment of Vaginal Microbiota Composition in Early Pregnancy and Its Association with Subsequent Preterm Prelabor Rupture of the Fetal Membranes. Transl. Res. 2019, 207, 30–43. 10.1016/j.trsl.2018.12.005. 30633889 PMC6489901

[ref184] Elovitz M. A.; Gajer P.; Riis V.; Brown A. G.; Humphrys M. S.; Holm J. B.; Ravel J. Cervicovaginal Microbiota and Local Immune Response Modulate the Risk of Spontaneous Preterm Delivery. Nat. Commun. 2019, 10, 1305 10.1038/s41467-019-09285-9. 30899005 PMC6428888

[ref185] Brown R. G.; Marchesi J. R.; Lee Y. S.; Smith A.; Lehne B.; Kindinger L. M.; Terzidou V.; Holmes E.; Nicholson J. K.; Bennett P. R.; et al. Vaginal Dysbiosis Increases Risk of Preterm Fetal Membrane Rupture, Neonatal Sepsis and Is Exacerbated by Erythromycin. BMC Med. 2018, 16, 9 10.1186/s12916-017-0999-x. 29361936 PMC5782380

[ref186] Freitas A. C.; Bocking A.; Hill J. E.; Money D. M.; Increased Richness and Diversity of the Vaginal Microbiota and Spontaneous Preterm Birth. Microbiome 2018, 6, 117 10.1186/s40168-018-0502-8. 29954448 PMC6022438

[ref187] McKinnon L. R.; Achilles S. L.; Bradshaw C. S.; Burgener A.; Crucitti T.; Fredricks D. N.; Jaspan H. B.; Kaul R.; Kaushic C.; Klatt N.; et al. The Evolving Facets of Bacterial Vaginosis: Implications for Hiv Transmission. AIDS Res. Hum. Retroviruses 2019, 35, 219–228. 10.1089/aid.2018.0304. 30638028 PMC6434601

[ref188] Han L.; Taub R.; Jensen J. T. Cervical Mucus and Contraception: What We Know and What We Don’t. Contraception 2017, 96, 310–321. 10.1016/j.contraception.2017.07.168. 28801053

[ref189] Lacroix G.; Gouyer V.; Gottrand F.; Desseyn J. L. The Cervicovaginal Mucus Barrier. Int. J. Mol. Sci. 2020, 21, 8266 10.3390/ijms21218266. 33158227 PMC7663572

[ref190] Domino S. E.; Hurd E. A.; Thomsson K. A.; Karnak D. M.; Holmen Larsson J. M.; Thomsson E.; Backstrom M.; Hansson G. C. Cervical Mucins Carry Alpha(1,2)Fucosylated Glycans That Partly Protect from Experimental Vaginal Candidiasis. Glycoconj. J. 2009, 26, 1125–1134. 10.1007/s10719-009-9234-0. 19326211 PMC2794911

[ref191] Cone R. A. Barrier Properties of Mucus. Adv. Drug. Delivery Rev. 2009, 61, 75–85. 10.1016/j.addr.2008.09.008. 19135107

[ref192] Agarwal K.; Lewis A. L. Vaginal Sialoglycan Foraging by Gardnerella Vaginalis: Mucus Barriers as a Meal for Unwelcome Guests?. Glycobiology 2021, 31, 667–680. 10.1093/glycob/cwab024. 33825850 PMC8252861

[ref193] Vagios S.; Mitchell C. M. Mutual Preservation: A Review of Interactions between Cervicovaginal Mucus and Microbiota. Front. Cell. Infect. Microbiol. 2021, 11, 676114 10.3389/fcimb.2021.676114. 34327149 PMC8313892

[ref194] Gipson I. K.; Moccia R.; Spurr-Michaud S.; Argueso P.; Gargiulo A. R.; Hill J. A. 3rd; Offner G. D.; Keutmann H. T. The Amount of Muc5b Mucin in Cervical Mucus Peaks at Midcycle. J. Clin. Endocrinol. Metab. 2001, 86, 594–600. 10.1210/jc.86.2.594. 11158014

[ref195] Mirmonsef P.; Hotton A. L.; Gilbert D.; Gioia C. J.; Maric D.; Hope T. J.; Landay A. L.; Spear G. T. Glycogen Levels in Undiluted Genital Fluid and Their Relationship to Vaginal Ph, Estrogen, and Progesterone. PLoS One 2016, 11, e0153553 10.1371/journal.pone.0153553. 27093050 PMC4836725

[ref196] Farage M.; Maibach H. Lifetime Changes in the Vulva and Vagina. Arch. Gynecol Obstet. 2006, 273, 195–202. 10.1007/s00404-005-0079-x. 16208476

[ref197] Seidman J. D.; Cho K. R.; Ronnett B. M.; Kurman R. J. Surface Epithelial Tumors of the Ovary. In Blaustein’s Pathology of the Female Genital Tract; Springer: 2011; pp 679–784.

[ref198] Tester R.; Al-Ghazzewi F. H. Intrinsic and Extrinsic Carbohydrates in the Vagina: A Short Review on Vaginal Glycogen. Int. J. Biol. Macromol. 2018, 112, 203–206. 10.1016/j.ijbiomac.2018.01.166. 29391223

[ref199] Mirmonsef P.; Hotton A. L.; Gilbert D.; Burgad D.; Landay A.; Weber K. M.; Cohen M.; Ravel J.; Spear G. T. Free Glycogen in Vaginal Fluids Is Associated with Lactobacillus Colonization and Low Vaginal Ph. PLoS One 2014, 9, e102467 10.1371/journal.pone.0102467. 25033265 PMC4102502

[ref200] Muhleisen A. L.; Herbst-Kralovetz M. M. Menopause and the Vaginal Microbiome. Maturitas 2016, 91, 42–50. 10.1016/j.maturitas.2016.05.015. 27451320

[ref201] McClelland R. S.; Lingappa J. R.; Srinivasan S.; Kinuthia J.; John-Stewart G. C.; Jaoko W.; Richardson B. A.; Yuhas K.; Fiedler T. L.; Mandaliya K. N.; et al. Evaluation of the Association between the Concentrations of Key Vaginal Bacteria and the Increased Risk of Hiv Acquisition in African Women from Five Cohorts: A Nested Case-Control Study. Lancet Infect. Dis. 2018, 18, 554–564. 10.1016/S1473-3099(18)30058-6. 29396006 PMC6445552

[ref202] Lee J. E.; Lee S.; Lee H.; Song Y. M.; Lee K.; Han M. J.; Sung J.; Ko G. Association of the Vaginal Microbiota with Human Papillomavirus Infection in a Korean Twin Cohort. PLoS One 2013, 8, e63514 10.1371/journal.pone.0063514. 23717441 PMC3661536

[ref203] Norenhag J.; Du J.; Olovsson M.; Verstraelen H.; Engstrand L.; Brusselaers N. The Vaginal Microbiota, Human Papillomavirus and Cervical Dysplasia: A Systematic Review and Network Meta-Analysis. BJOG 2020, 127, 171–180. 10.1111/1471-0528.15854. 31237400

[ref204] Lev-Sagie A.; Goldman-Wohl D.; Cohen Y.; Dori-Bachash M.; Leshem A.; Mor U.; Strahilevitz J.; Moses A. E.; Shapiro H.; Yagel S.; et al. Vaginal Microbiome Transplantation in Women with Intractable Bacterial Vaginosis. Nat. Med. 2019, 25, 1500–1504. 10.1038/s41591-019-0600-6. 31591599

[ref205] Gilbert J. A.; Quinn R. A.; Debelius J.; Xu Z. Z.; Morton J.; Garg N.; Jansson J. K.; Dorrestein P. C.; Knight R. Microbiome-Wide Association Studies Link Dynamic Microbial Consortia to Disease. Nature 2016, 535, 94–103. 10.1038/nature18850. 27383984

[ref206] Manor O.; Dai C. L.; Kornilov S. A.; Smith B.; Price N. D.; Lovejoy J. C.; Gibbons S. M.; Magis A. T. Health and Disease Markers Correlate with Gut Microbiome Composition across Thousands of People. Nature Commun. 2020, 11, 5206 10.1038/s41467-020-18871-1. 33060586 PMC7562722

[ref207] Falony G.; Joossens M.; Vieira-Silva S.; Wang J.; Darzi Y.; Faust K.; Kurilshikov A.; Bonder M. J.; Valles-Colomer M.; Vandeputte D.; et al. Population-Level Analysis of Gut Microbiome Variation. Science 2016, 352, 560–564. 10.1126/science.aad3503. 27126039

[ref208] Schnorr S. L.; Candela M.; Rampelli S.; Centanni M.; Consolandi C.; Basaglia G.; Turroni S.; Biagi E.; Peano C.; Severgnini M.; et al. Gut Microbiome of the Hadza Hunter-Gatherers. Nature Commun. 2014, 5, 3654 10.1038/ncomms4654. 24736369 PMC3996546

[ref209] Koliada A.; Syzenko G.; Moseiko V.; Budovska L.; Puchkov K.; Perederiy V.; Gavalko Y.; Dorofeyev A.; Romanenko M.; Tkach S.; et al. Association between Body Mass Index and Firmicutes/Bacteroidetes Ratio in an Adult Ukrainian Population. BMC Microbiology 2017, 17, 120 10.1186/s12866-017-1027-1. 28532414 PMC5440985

[ref210] Verdam F. J.; Fuentes S.; de Jonge C.; Zoetendal E. G.; Erbil R.; Greve J. W.; Buurman W. A.; de Vos W. M.; Rensen S. S. Human Intestinal Microbiota Composition Is Associated with Local and Systemic Inflammation in Obesity. Obesity 2013, 21, E607–E615. 10.1002/oby.20466. 23526699

[ref211] Kasai C.; Sugimoto K.; Moritani I.; Tanaka J.; Oya Y.; Inoue H.; Tameda M.; Shiraki K.; Ito M.; Takei Y.; et al. Comparison of the Gut Microbiota Composition between Obese and Non-Obese Individuals in a Japanese Population, as Analyzed by Terminal Restriction Fragment Length Polymorphism and Next-Generation Sequencing. BMC Gastroenterol. 2015, 15, 100 10.1186/s12876-015-0330-2. 26261039 PMC4531509

[ref212] Crovesy L.; Masterson D.; Rosado E. L. Profile of the Gut Microbiota of Adults with Obesity: A Systematic Review. Eur. J. Clin. Nutr. 2020, 74, 1251–1262. 10.1038/s41430-020-0607-6. 32231226

[ref213] Manichanh C.; Rigottier-Gois L.; Bonnaud E.; Gloux K.; Pelletier E.; Frangeul L.; Nalin R.; Jarrin C.; Chardon P.; Marteau P.; et al. Reduced Diversity of Faecal Microbiota in Crohn’s Disease Revealed by a Metagenomic Approach. Gut 2006, 55, 205 10.1136/gut.2005.073817. 16188921 PMC1856500

[ref214] Vester-Andersen M. K.; Mirsepasi-Lauridsen H. C.; Prosberg M. V.; Mortensen C. O.; Träger C.; Skovsen K.; Thorkilgaard T.; Nøjgaard C.; Vind I.; Krogfelt K. A.; et al. Increased Abundance of Proteobacteria in Aggressive Crohn’s Disease Seven Years after Diagnosis. Sci. Rep. 2019, 9, 13473 10.1038/s41598-019-49833-3. 31530835 PMC6748953

[ref215] Huang T.-T.; Lai J.-B.; Du Y.-L.; Xu Y.; Ruan L.-M.; Hu S.-H. Current Understanding of Gut Microbiota in Mood Disorders: An Update of Human Studies. Front. Genet. 2019, 10, 98 10.3389/fgene.2019.00098. 30838027 PMC6389720

[ref216] Giloteaux L.; Goodrich J. K.; Walters W. A.; Levine S. M.; Ley R. E.; Hanson M. R. Reduced Diversity and Altered Composition of the Gut Microbiome in Individuals with Myalgic Encephalomyelitis/Chronic Fatigue Syndrome. Microbiome 2016, 4, 30 10.1186/s40168-016-0171-4. 27338587 PMC4918027

[ref217] Golfetto L.; de Senna F. D.; Hermes J.; Beserra B. T.; França F. d. S.; Martinello F. Lower Bifidobacteria Counts in Adult Patients with Celiac Disease on a Gluten-Free Diet. Arq. Gastroenterol. 2014, 51, 139–143. 10.1590/S0004-28032014000200013. 25003267

[ref218] Kerckhoffs A. P.; Samsom M.; van der Rest M. E.; de Vogel J.; Knol J.; Ben-Amor K.; Akkermans L. M. Lower Bifidobacteria Counts in Both Duodenal Mucosa-Associated and Fecal Microbiota in Irritable Bowel Syndrome Patients. World J. Gastroenterol. 2009, 15, 2887–2892. 10.3748/wjg.15.2887. 19533811 PMC2699007

[ref219] Lee H.-J.; Lee K.-E.; Kim J.-K.; Kim D.-H. Suppression of Gut Dysbiosis by Bifidobacterium Longum Alleviates Cognitive Decline in 5xfad Transgenic and Aged Mice. Sci. Rep. 2019, 9, 11814 10.1038/s41598-019-48342-7. 31413350 PMC6694197

[ref220] Kong H. H.; Oh J.; Deming C.; Conlan S.; Grice E. A.; Beatson M. A.; Nomicos E.; Polley E. C.; Komarow H. D.; et al. Temporal Shifts in the Skin Microbiome Associated with Disease Flares and Treatment in Children with Atopic Dermatitis. Genome Res. 2012, 22, 850–859. 10.1101/gr.131029.111. 22310478 PMC3337431

[ref221] Takahashi Y.; Saito A.; Chiba H.; Kuronuma K.; Ikeda K.; Kobayashi T.; Ariki S.; Takahashi M.; Sasaki Y.; Takahashi H. Impaired Diversity of the Lung Microbiome Predicts Progression of Idiopathic Pulmonary Fibrosis. Respir. Res. 2018, 19, 34 10.1186/s12931-018-0736-9. 29486761 PMC6389110

[ref222] Thomas-White K. J.; Gao X.; Lin H.; Fok C. S.; Ghanayem K.; Mueller E. R.; Dong Q.; Brubaker L.; Wolfe A. J. Urinary Microbes and Postoperative Urinary Tract Infection Risk in Urogynecologic Surgical Patients. Int.Urogynecol. J. 2018, 29, 1797–1805. 10.1007/s00192-018-3767-3. 30267143 PMC6527134

[ref223] Curtiss N.; Balachandran A.; Krska L.; Peppiatt-Wildman C.; Wildman S.; Duckett J. A Case Controlled Study Examining the Bladder Microbiome in Women with Overactive Bladder (Oab) and Healthy Controls. Eur. J. Obstet. Gynecol. Reprod. Biol. 2017, 214, 31–35. 10.1016/j.ejogrb.2017.04.040. 28463826

[ref224] Joung H.; Chu J.; Kim B.-K.; Choi I.-S.; Kim W.; Park T.-S. Probiotics Ameliorate Chronic Low-Grade Inflammation and Fat Accumulation with Gut Microbiota Composition Change in Diet-Induced Obese Mice Models. Appl. Microbiol. Biotechnol. 2021, 105, 1203–1213. 10.1007/s00253-020-11060-6. 33443636

[ref225] Cheng Y.-C.; Liu J.-R. Effect of Lactobacillus Rhamnosus Gg on Energy Metabolism, Leptin Resistance, and Gut Microbiota in Mice with Diet-Induced Obesity. Nutrients 2020, 12, 2557 10.3390/nu12092557. 32846917 PMC7551584

[ref226] Borgeraas H.; Johnson L. K.; Skattebu J.; Hertel J. K.; Hjelmesæth J. Effects of Probiotics on Body Weight, Body Mass Index, Fat Mass and Fat Percentage in Subjects with Overweight or Obesity: A Systematic Review and Meta-Analysis of Randomized Controlled Trials. Obes. Rev. 2018, 19, 219–232. 10.1111/obr.12626. 29047207

[ref227] Larsen N.; Vogensen F. K.; Gøbel R. J.; Michaelsen K. F.; Forssten S. D.; Lahtinen S. J.; Jakobsen M. Effect of Lactobacillus Salivarius Ls-33 on Fecal Microbiota in Obese Adolescents. Clin. Nutr. 2013, 32, 935–940. 10.1016/j.clnu.2013.02.007. 23510724

[ref228] Gøbel R. J.; Larsen N.; Jakobsen M.; Mølgaard C.; Michaelsen K. F. Probiotics to Adolescents with Obesity: Effects on Inflammation and Metabolic Syndrome. J. Pediatr. Gastroenterol. Nutr. 2012, 55, 673–678. 10.1097/MPG.0b013e318263066c. 22695039

[ref229] Cani P. D.; de Vos W. M. Next-Generation Beneficial Microbes: The Case of Akkermansia Muciniphila. Front. Microbiol. 2017, 8, 1765 10.3389/fmicb.2017.01765. 29018410 PMC5614963

[ref230] Depommier C.; Everard A.; Druart C.; Plovier H.; Van Hul M.; Vieira-Silva S.; Falony G.; Raes J.; Maiter D.; Delzenne N. M.; et al. Supplementation with Akkermansia Muciniphila in Overweight and Obese Human Volunteers: A Proof-of-Concept Exploratory Study. Nat. Med. 2019, 25, 1096–1103. 10.1038/s41591-019-0495-2. 31263284 PMC6699990

[ref231] Plovier H.; Everard A.; Druart C.; Depommier C.; Van Hul M.; Geurts L.; Chilloux J.; Ottman N.; Duparc T.; Lichtenstein L.; et al. A Purified Membrane Protein from Akkermansia Muciniphila or the Pasteurized Bacterium Improves Metabolism in Obese and Diabetic Mice. Nat. Med. 2017, 23, 107–113. 10.1038/nm.4236. 27892954

[ref232] Wang Y.; Guo Y.; Chen H.; Wei H.; Wan C. Potential of Lactobacillus Plantarum Zdy2013 and Bifidobacterium Bifidum Wbin03 in Relieving Colitis by Gut Microbiota, Immune, and Anti-Oxidative Stress. Can. J. Microbiol. 2018, 64, 327–337. 10.1139/cjm-2017-0716. 29401402

[ref233] Jang Y. J.; Kim W.-K.; Han D. H.; Lee K.; Ko G. Lactobacillus Fermentum Species Ameliorate Dextran Sulfate Sodium-Induced Colitis by Regulating the Immune Response and Altering Gut Microbiota. Gut Microbes 2019, 10, 696–711. 10.1080/19490976.2019.1589281. 30939976 PMC6866707

[ref234] Bjarnason I.; Sission G.; Hayee B. A Randomised, Double-Blind, Placebo-Controlled Trial of a Multi-Strain Probiotic in Patients with Asymptomatic Ulcerative Colitis and Crohn’s Disease. Inflammopharmacology 2019, 27, 465–473. 10.1007/s10787-019-00595-4. 31054010 PMC6554453

[ref235] Tamaki H.; Nakase H.; Inoue S.; Kawanami C.; Itani T.; Ohana M.; Kusaka T.; Uose S.; Hisatsune H.; Tojo M.; et al. Efficacy of Probiotic Treatment with Bifidobacterium Longum 536 for Induction of Remission in Active Ulcerative Colitis: A Randomized, Double-Blinded, Placebo-Controlled Multicenter Trial. Dig. Endosc. 2016, 28, 67–74. 10.1111/den.12553. 26418574

[ref236] Martyniak A.; Medyńska-Przęczek A.; Wędrychowicz A.; Skoczeń S.; Tomasik P. J. Prebiotics, Probiotics, Synbiotics, Paraprobiotics and Postbiotic Compounds in Ibd. Biomolecules 2021, 11, 1903 10.3390/biom11121903. 34944546 PMC8699341

[ref237] Glassner K. L.; Abraham B. P.; Quigley E. M. M. The Microbiome and Inflammatory Bowel Disease. J. Allergy Clin. Immunol. 2020, 145, 16–27. 10.1016/j.jaci.2019.11.003. 31910984

[ref238] Cao Y.; Shen J.; Ran Z. H. Association between Faecalibacterium Prausnitzii Reduction and Inflammatory Bowel Disease: A Meta-Analysis and Systematic Review of the Literature. Gastroenterol. Res. Pract. 2014, 2014, 872725 10.1155/2014/872725. 24799893 PMC3985188

[ref239] Zhao H.; Xu H.; Chen S.; He J.; Zhou Y.; Nie Y. Systematic Review and Meta-Analysis of the Role of Faecalibacterium Prausnitzii Alteration in Inflammatory Bowel Disease. J. Gastroenterol. Hepatol. 2021, 36, 320–328. 10.1111/jgh.15222. 32815163

[ref240] Qiu X.; Zhang M.; Yang X.; Hong N.; Yu C. Faecalibacterium Prausnitzii Upregulates Regulatory T Cells and Anti-Inflammatory Cytokines in Treating Tnbs-Induced Colitis. J. Crohns Colitis 2013, 7, e558–e568. 10.1016/j.crohns.2013.04.002. 23643066

[ref241] Martín R.; Chain F.; Miquel S.; Lu J.; Gratadoux J.-J.; Sokol H.; Verdu E. F.; Bercik P.; Bermúdez-Humarán L. G.; Langella P. The Commensal Bacterium Faecalibacterium Prausnitzii Is Protective in Dnbs-Induced Chronic Moderate and Severe Colitis Models. Inflamm. Bowel Dis. 2014, 20, 417–430. 10.1097/01.MIB.0000440815.76627.64. 24418903

[ref242] He X.; Zhao S.; Li Y. Faecalibacterium Prausnitzii: A Next-Generation Probiotic in Gut Disease Improvement. Can. J. Infect. Dis. Med. Microbiol. 2021, 2021, 6666114 10.1155/2021/6666114.

[ref243] Sokol H.; Leducq V.; Aschard H.; Pham H.-P.; Jegou S.; Landman C.; Cohen D.; Liguori G.; Bourrier A.; Nion-Larmurier I.; et al. Fungal Microbiota Dysbiosis in Ibd. Gut 2017, 66, 1039 10.1136/gutjnl-2015-310746. 26843508 PMC5532459

[ref244] Limon J. J.; Tang J.; Li D.; Wolf A. J.; Michelsen K. S.; Funari V.; Gargus M.; Nguyen C.; Sharma P.; Maymi V. I.; et al. Malassezia Is Associated with Crohn’s Disease and Exacerbates Colitis in Mouse Models. Cell Host Microbe 2019, 25, 377–388. 10.1016/j.chom.2019.01.007. 30850233 PMC6417942

[ref245] Norman J. M.; Handley S. A.; Baldridge M. T.; Droit L.; Liu C. Y.; Keller B. C.; Kambal A.; Monaco C. L.; Zhao G.; Fleshner P.; et al. Disease-Specific Alterations in the Enteric Virome in Inflammatory Bowel Disease. Cell 2015, 160, 447–460. 10.1016/j.cell.2015.01.002. 25619688 PMC4312520

[ref246] Kelesidis T.; Pothoulakis C. Efficacy and Safety of the Probiotic Saccharomyces Boulardii for the Prevention and Therapy of Gastrointestinal Disorders. Therap. Adv. Gastroenterol. 2012, 5, 111–125. 10.1177/1756283X11428502. PMC3296087 22423260

[ref247] Myles I. A.; Williams K. W.; Reckhow J. D.; Jammeh M. L.; Pincus N. B.; Sastalla I.; Saleem D.; Stone K. D.; Datta S. K. Transplantation of Human Skin Microbiota in Models of Atopic Dermatitis. JCI Insight 2016, 1, e86955 10.1172/jci.insight.86955. 27478874 PMC4963067

[ref248] Myles I. A.; Castillo C. R.; Barbian K. D.; Kanakabandi K.; Virtaneva K.; Fitzmeyer E.; Paneru M.; Otaizo-Carrasquero F.; Myers T. G.; Markowitz T. E.; et al. Therapeutic Responses to Roseomonas Mucosa in Atopic Dermatitis May Involve Lipid-Mediated Tnf-Related Epithelial Repair. Sci. Transl. Med. 2020, 12, eaaz8631 10.1126/scitranslmed.aaz8631. 32908007 PMC8571514

[ref249] Gupta K.; Stapleton A. E.; Hooton T. M.; Roberts P. L.; Fennell C. L.; Stamm W. E. Inverse Association of H2o2-Producing Lactobacilli and Vaginal Escherichia Coli Colonization in Women with Recurrent Urinary Tract Infections. J. Infect. Dis. 1998, 178, 446–450. 10.1086/515635. 9697725

[ref250] Kwok L.; Stapleton A. E.; Stamm W. E.; Hillier S. L.; Wobbe C. L.; Gupta K. Adherence of Lactobacillus Crispatus to Vaginal Epithelial Cells from Women with or without a History of Recurrent Urinary Tract Infection. J. Urol. 2006, 176, 2050–2054. 10.1016/j.juro.2006.07.014. 17070251

[ref251] Stapleton A. E.; Au-Yeung M.; Hooton T. M.; Fredricks D. N.; Roberts P. L.; Czaja C. A.; Yarova-Yarovaya Y.; Fiedler T.; Cox M.; Stamm W. E. Randomized, Placebo-Controlled Phase 2 Trial of a Lactobacillus Crispatus Probiotic Given Intravaginally for Prevention of Recurrent Urinary Tract Infection. Clin. Infect. Dis. 2011, 52, 1212–1217. 10.1093/cid/cir183. 21498386 PMC3079401

[ref252] Sanders M. E.; Merenstein D. J.; Reid G.; Gibson G. R.; Rastall R. A. Probiotics and Prebiotics in Intestinal Health and Disease: From Biology to the Clinic. Nat. Rev. Gastroenterol. Hepatol. 2019, 16, 605–616. 10.1038/s41575-019-0173-3. 31296969

[ref253] Macfarlane S.; Macfarlane G. T.; Cummings J. H. Review Article: Prebiotics in the Gastrointestinal Tract. Aliment Pharmacol Ther. 2006, 24, 701–714. 10.1111/j.1365-2036.2006.03042.x. 16918875

[ref254] Liu F.; Li P.; Chen M.; Luo Y.; Prabhakar M.; Zheng H.; He Y.; Qi Q.; Long H.; Zhang Y.; et al. Fructooligosaccharide (FOS) and Galactooligosaccharide (GOS) Increase Bifidobacterium but Reduce Butyrate Producing Bacteria with Adverse Glycemic Metabolism in Healthy Young Population. Sci. Rep. 2017, 7, 11789 10.1038/s41598-017-10722-2. 28924143 PMC5603605

[ref255] Tandon D.; Haque M. M.; Gote M.; Jain M.; Bhaduri A.; Dubey A. K.; Mande S. S. A Prospective Randomized, Double-Blind, Placebo-Controlled, Dose-Response Relationship Study to Investigate Efficacy of Fructo-Oligosaccharides (Fos) on Human Gut Microflora. Sci. Rep. 2019, 9, 5473 10.1038/s41598-019-41837-3. 30940833 PMC6445088

[ref256] Finegold S. M.; Li Z.; Summanen P. H.; Downes J.; Thames G.; Corbett K.; Dowd S.; Krak M.; Heber D. Xylooligosaccharide Increases Bifidobacteria but Not Lactobacilli in Human Gut Microbiota. Food Funct. 2014, 5, 436–445. 10.1039/c3fo60348b. 24513849

[ref257] Björkstén B.; Sepp E.; Julge K.; Voor T.; Mikelsaar M. Allergy Development and the Intestinal Microflora During the First Year of Life. J. Allergy. Clin. Immunol. 2001, 108, 516–520. 10.1067/mai.2001.118130. 11590374

[ref258] Moro G.; Arslanoglu S.; Stahl B.; Jelinek J.; Wahn U.; Boehm G. A Mixture of Prebiotic Oligosaccharides Reduces the Incidence of Atopic Dermatitis During the First Six Months of Age. Arch. Dis. Child 2006, 91, 814–819. 10.1136/adc.2006.098251. 16873437 PMC2066015

[ref259] Arslanoglu S.; Moro G. E.; Schmitt J.; Tandoi L.; Rizzardi S.; Boehm G. Early Dietary Intervention with a Mixture of Prebiotic Oligosaccharides Reduces the Incidence of Allergic Manifestations and Infections During the First Two Years of Life. J. Nut. 2008, 138, 1091–1095. 10.1093/jn/138.6.1091. 18492839

[ref260] Lindsay J. O.; Whelan K.; Stagg A. J.; Gobin P.; Al-Hassi H. O.; Rayment N.; Kamm M. A.; Knight S. C.; Forbes A. Clinical, Microbiological, and Immunological Effects of Fructo-Oligosaccharide in Patients with Crohn’s Disease. Gut 2006, 55, 348–355. 10.1136/gut.2005.074971. 16162680 PMC1856087

[ref261] Han K.; Nam J.; Xu J.; Sun X.; Huang X.; Animasahun O.; Achreja A.; Jeon J. H.; Pursley B.; Kamada N.; et al. Generation of Systemic Antitumour Immunity Via the in Situ Modulation of the Gut Microbiome by an Orally Administered Inulin Gel. Nat. Biomed. Eng. 2021, 5, 1377–1388. 10.1038/s41551-021-00749-2. 34168321 PMC8595497

[ref262] Routy B.; Le Chatelier E.; Derosa L.; Duong C. P. M.; Alou M. T.; Daillère R.; Fluckiger A.; Messaoudene M.; Rauber C.; Roberti M. P.; et al. Gut Microbiome Influences Efficacy of Pd-1–Based Immunotherapy against Epithelial Tumors. Science 2018, 359, 91–97. 10.1126/science.aan3706. 29097494

[ref263] Gopalakrishnan V.; Spencer C. N.; Nezi L.; Reuben A.; Andrews M. C.; Karpinets T. V.; Prieto P. A.; Vicente D.; Hoffman K.; Wei S. C.; et al. Gut Microbiome Modulates Response to Anti–Pd-1 Immunotherapy in Melanoma Patients. Science 2018, 359, 97–103. 10.1126/science.aan4236. 29097493 PMC5827966

[ref264] Matson V.; Fessler J.; Bao R.; Chongsuwat T.; Zha Y.; Alegre M.-L.; Luke J. J.; Gajewski T. F. The Commensal Microbiome Is Associated with Anti–Pd-1 Efficacy in Metastatic Melanoma Patients. Science 2018, 359, 104–108. 10.1126/science.aao3290. 29302014 PMC6707353

[ref265] Dethlefsen L.; Relman D. A. Incomplete Recovery and Individualized Responses of the Human Distal Gut Microbiota to Repeated Antibiotic Perturbation. Proc. Natl. Acad. Sci. U.S.A. 2011, 108, 4554–4561. 10.1073/pnas.1000087107. 20847294 PMC3063582

[ref266] Francino M. P. Antibiotics and the Human Gut Microbiome: Dysbioses and Accumulation of Resistances. Front. Microbiol. 2016, 6, 1543 10.3389/fmicb.2015.01543. 26793178 PMC4709861

[ref267] Raymond F.; Ouameur A. A.; Déraspe M.; Iqbal N.; Gingras H.; Dridi B.; Leprohon P.; Plante P.-L.; Giroux R.; Bérubé È.; et al. The Initial State of the Human Gut Microbiome Determines Its Reshaping by Antibiotics. ISME J. 2016, 10, 707–720. 10.1038/ismej.2015.148. 26359913 PMC4817689

[ref268] Vickers R. J.; Tillotson G.; Goldstein E. J. C.; Citron D. M.; Garey K. W.; Wilcox M. H. Ridinilazole: A Novel Therapy for Clostridium Difficile Infection. Int. J. Antimicrob. Agents 2016, 48, 137–143. 10.1016/j.ijantimicag.2016.04.026. 27283730

[ref269] Vickers R. J.; Tillotson G. S.; Nathan R.; Hazan S.; Pullman J.; Lucasti C.; Deck K.; Yacyshyn B.; Maliakkal B.; Pesant Y.; et al. Efficacy and Safety of Ridinilazole Compared with Vancomycin for the Treatment of Clostridium Difficile Infection: A Phase 2, Randomised, Double-Blind, Active-Controlled, Non-Inferiority Study. Lancet Infect. Dis. 2017, 17, 735–744. 10.1016/S1473-3099(17)30235-9. 28461207 PMC5483507

[ref270] Thorpe C. M.; Kane A. V.; Chang J.; Tai A.; Vickers R. J.; Snydman D. R. Enhanced Preservation of the Human Intestinal Microbiota by Ridinilazole, a Novel Clostridium Difficile-Targeting Antibacterial, Compared to Vancomycin. PLoS One 2018, 13, e0199810 10.1371/journal.pone.0199810. 30071046 PMC6071993

[ref271] Avis T.; Wilson F. X.; Khan N.; Mason C. S.; Powell D. J. Targeted Microbiome-Sparing Antibiotics. Drug Discovery 2021, 26, 2198–2203. 10.1016/j.drudis.2021.07.016. 34329771

[ref272] Machado A.; Cerca N. Influence of Biofilm Formation by Gardnerella Vaginalis and Other Anaerobes on Bacterial Vaginosis. J. Infect. Dis. 2015, 212, 1856–1861. 10.1093/infdis/jiv338. 26080369

[ref273] Bradshaw C. S.; Sobel J. D. Current Treatment of Bacterial Vaginosis—Limitations and Need for Innovation. J. Infect. Dis. 2016, 214, S14–S20. 10.1093/infdis/jiw159. 27449869 PMC4957510

[ref274] Landlinger C.; Tisakova L.; Oberbauer V.; Schwebs T.; Muhammad A.; Latka A.; Van Simaey L.; Vaneechoutte M.; Guschin A.; Resch G.; et al. Engineered Phage Endolysin Eliminates Gardnerella Biofilm without Damaging Beneficial Bacteria in Bacterial Vaginosis Ex Vivo. Pathogens 2021, 10, 54 10.3390/pathogens10010054. 33435575 PMC7830407

[ref275] Whitney C. G.; Farley M. M.; Hadler J.; Harrison L. H.; Bennett N. M.; Lynfield R.; Reingold A.; Cieslak P. R.; Pilishvili T.; Jackson D.; et al. Decline in Invasive Pneumococcal Disease after the Introduction of Protein–Polysaccharide Conjugate Vaccine. N. Engl. J. Med. 2003, 348, 1737–1746. 10.1056/NEJMoa022823. 12724479

[ref276] Biesbroek G.; Wang X.; Keijser B. J.; Eijkemans R. M.; Trzciński K.; Rots N. Y.; Veenhoven R. H.; Sanders E. A.; Bogaert D. Seven-Valent Pneumococcal Conjugate Vaccine and Nasopharyngeal Microbiota in Healthy Children. Emerg. Infect. Dis. 2014, 20, 201–210. 10.3201/eid2002.131220. 24447437 PMC3901477

[ref277] Blaser M. J.; Falkow S. What Are the Consequences of the Disappearing Human Microbiota?. Nat. Rev. Microbiol. 2009, 7, 887–894. 10.1038/nrmicro2245. 19898491 PMC9354563

[ref278] Mika M.; Maurer J.; Korten I.; Allemann A.; Aebi S.; Brugger S. D.; Qi W.; Frey U.; Latzin P.; Hilty M. Influence of the Pneumococcal Conjugate Vaccines on the Temporal Variation of Pneumococcal Carriage and the Nasal Microbiota in Healthy Infants: A Longitudinal Analysis of a Case–Control Study. Microbiome 2017, 5, 85 10.1186/s40168-017-0302-6. 28738889 PMC5525364

[ref279] Relman D. A.; Lipsitch M. Microbiome as a Tool and a Target in the Effort to Address Antimicrobial Resistance. Proc. Natl. Acad. Sci. U.S.A. 2018, 115, 12902–12910. 10.1073/pnas.1717163115. 30559176 PMC6304941

[ref280] Brown R.; Lengeling A.; Wang B. Phage Engineering: How Advances in Molecular Biology and Synthetic Biology Are Being Utilized to Enhance the Therapeutic Potential of Bacteriophages. Quant. Biol. 2017, 5, 42–54. 10.1007/s40484-017-0094-5.

[ref281] Zhang Y.; Li C.-X.; Zhang X.-Z. Bacteriophage-Mediated Modulation of Microbiota for Diseases Treatment. Adv. Drug Delivery Rev. 2021, 176, 113856 10.1016/j.addr.2021.113856. 34237403

[ref282] Duan Y.; Llorente C.; Lang S.; Brandl K.; Chu H.; Jiang L.; White R. C.; Clarke T. H.; Nguyen K.; Torralba M.; et al. Bacteriophage Targeting of Gut Bacterium Attenuates Alcoholic Liver Disease. Nature 2019, 575, 505–511. 10.1038/s41586-019-1742-x. 31723265 PMC6872939

[ref283] Zheng D.-W.; Dong X.; Pan P.; Chen K.-W.; Fan J.-X.; Cheng S.-X.; Zhang X.-Z. Phage-Guided Therapeutic System for Cancer Therapy by Modulating Gut Microbiota. Nat. Biomed. Eng. 2019, 3, 717–728. 10.1038/s41551-019-0423-2. 31332342

[ref284] Yu T.; Guo F.; Yu Y.; Sun T.; Ma D.; Han J.; Qian Y.; Kryczek I.; Sun D.; Nagarsheth N. Fusobacterium Nucleatum Promotes Chemoresistance to Colorectal Cancer by Modulating Autophagy. Cell 2017, 170, 548–563. 10.1016/j.cell.2017.07.008. 28753429 PMC5767127

[ref285] Castillo D. E.; Nanda S.; Keri J. E. Propionibacterium (Cutibacterium) Acnes Bacteriophage Therapy in Acne: Current Evidence and Future Perspectives. Dermatol. Ther. 2019, 9, 19–31. 10.1007/s13555-018-0275-9. PMC6380980 30539425

[ref286] Brown T. L.; Petrovski S.; Dyson Z. A.; Seviour R.; Tucci J. The Formulation of Bacteriophage in a Semi Solid Preparation for Control of Propionibacterium Acnes Growth. PLoS One 2016, 11, e0151184 10.1371/journal.pone.0151184. 26964063 PMC4786141

[ref287] Bermudez-Brito M.; Plaza-Díaz J.; Muñoz-Quezada S.; Gómez-Llorente C.; Gil A. Probiotic Mechanisms of Action. Ann. Nutr. Metab. 2012, 61, 160–174. 10.1159/000342079. 23037511

[ref288] Hwang I. Y.; Koh E.; Wong A.; March J. C.; Bentley W. E.; Lee Y. S.; Chang M. W. Engineered Probiotic Escherichia Coli Can Eliminate and Prevent Pseudomonas Aeruginosa Gut Infection in Animal Models. Nat. Commun. 2017, 8, 15028 10.1038/ncomms15028. 28398304 PMC5394271

[ref289] Lubkowicz D.; Ho C. L.; Hwang I. Y.; Yew W. S.; Lee Y. S.; Chang M. W. Reprogramming Probiotic Lactobacillus Reuteri as a Biosensor for Staphylococcus Aureus Derived Aip-I Detection. ACS Synth. Biol. 2018, 7, 1229–1237. 10.1021/acssynbio.8b00063. 29652493

[ref290] Jayaraman P.; Holowko M. B.; Yeoh J. W.; Lim S.; Poh C. L. Repurposing a Two-Component System-Based Biosensor for the Killing of Vibrio Cholerae. ACS Synth. Biol. 2017, 6, 1403–1415. 10.1021/acssynbio.7b00058. 28441472

[ref291] Sommer F.; Anderson J. M.; Bharti R.; Raes J.; Rosenstiel P. The Resilience of the Intestinal Microbiota Influences Health and Disease. Nat. Rev. Microbiol. 2017, 15, 630–638. 10.1038/nrmicro.2017.58. 28626231

[ref292] Bikard D.; Euler C. W.; Jiang W.; Nussenzweig P. M.; Goldberg G. W.; Duportet X.; Fischetti V. A.; Marraffini L. A. Exploiting Crispr-Cas Nucleases to Produce Sequence-Specific Antimicrobials. Nat. Biotechnol. 2014, 32, 1146–1150. 10.1038/nbt.3043. 25282355 PMC4317352

[ref293] Citorik R. J.; Mimee M.; Lu T. K. Sequence-Specific Antimicrobials Using Efficiently Delivered Rna-Guided Nucleases. Nat. Biotechnol. 2014, 32, 1141–1145. 10.1038/nbt.3011. 25240928 PMC4237163

[ref294] Lam K. N.; Spanogiannopoulos P.; Soto-Perez P.; Alexander M.; Nalley M. J.; Bisanz J. E.; Nayak R. R.; Weakley A. M.; Yu F. B.; Turnbaugh P. J. Phage-Delivered Crispr-Cas9 for Strain-Specific Depletion and Genomic Deletions in the Gut Microbiome. Cell Rep. 2021, 37, 109930 10.1016/j.celrep.2021.109930. 34731631 PMC8591988

[ref295] Ting S.-Y.; Martínez-García E.; Huang S.; Bertolli S. K.; Kelly K. A.; Cutler K. J.; Su E. D.; Zhi H.; Tang Q.; Radey M. C.; et al. Targeted Depletion of Bacteria from Mixed Populations by Programmable Adhesion with Antagonistic Competitor Cells. Cell Host Microbe 2020, 28, 313–321. 10.1016/j.chom.2020.05.006. 32470328 PMC7725374

[ref296] Turnbaugh P. J.; Hamady M.; Yatsunenko T.; Cantarel B. L.; Duncan A.; Ley R. E.; Sogin M. L.; Jones W. J.; Roe B. A.; Affourtit J. P.; et al. A Core Gut Microbiome in Obese and Lean Twins. Nature 2009, 457, 480–484. 10.1038/nature07540. 19043404 PMC2677729

[ref297] Moya A.; Ferrer M. Functional Redundancy-Induced Stability of Gut Microbiota Subjected to Disturbance. Trends Microbiol. 2016, 24, 402–413. 10.1016/j.tim.2016.02.002. 26996765

[ref298] Morgan X. C.; Tickle T. L.; Sokol H.; Gevers D.; Devaney K. L.; Ward D. V.; Reyes J. A.; Shah S. A.; LeLeiko N.; Snapper S. B.; et al. Dysfunction of the Intestinal Microbiome in Inflammatory Bowel Disease and Treatment. Genome Biol. 2012, 13, R79 10.1186/gb-2012-13-9-r79. 23013615 PMC3506950

[ref299] Vázquez-Castellanos J. F.; Serrano-Villar S.; Latorre A.; Artacho A.; Ferrús M. L.; Madrid N.; Vallejo A.; Sainz T.; Martínez-Botas J.; Ferrando-Martínez S.; et al. Altered Metabolism of Gut Microbiota Contributes to Chronic Immune Activation in Hiv-Infected Individuals. Mucosal Immunol. 2015, 8, 760–772. 10.1038/mi.2014.107. 25407519

[ref300] Sheth R. U.; Cabral V.; Chen S. P.; Wang H. H. Manipulating Bacterial Communities by in Situ Microbiome Engineering. Trends Genet. 2016, 32, 189–200. 10.1016/j.tig.2016.01.005. 26916078 PMC4828914

[ref301] Brophy J. A. N.; Triassi A. J.; Adams B. L.; Renberg R. L.; Stratis-Cullum D. N.; Grossman A. D.; Voigt C. A. Engineered Integrative and Conjugative Elements for Efficient and Inducible DNA Transfer to Undomesticated Bacteria. Nat. Microbiol. 2018, 3, 1043–1053. 10.1038/s41564-018-0216-5. 30127494

[ref302] Ronda C.; Chen S. P.; Cabral V.; Yaung S. J.; Wang H. H. Metagenomic Engineering of the Mammalian Gut Microbiome in Situ. Nat. Methods 2019, 16, 167–170. 10.1038/s41592-018-0301-y. 30643213 PMC6467691

[ref303] Jin W.-B.; Li T.-T.; Huo D.; Qu S.; Li X. V.; Arifuzzaman M.; Lima S. F.; Shi H.-Q.; Wang A.; Putzel G. G.; et al. Genetic Manipulation of Gut Microbes Enables Single-Gene Interrogation in a Complex Microbiome. Cell 2022, 185, 547–562. 10.1016/j.cell.2021.12.035. 35051369 PMC8919858

[ref304] Liu L.; Chen X.; Skogerbø G.; Zhang P.; Chen R.; He S.; Huang D.-W. The Human Microbiome: A Hot Spot of Microbial Horizontal Gene Transfer. Genomics 2012, 100, 265–270. 10.1016/j.ygeno.2012.07.012. 22841660

[ref305] Wilson I. D.; Nicholson J. K. Gut Microbiome Interactions with Drug Metabolism, Efficacy, and Toxicity. Transl. Res. 2017, 179, 204–222. 10.1016/j.trsl.2016.08.002. 27591027 PMC5718288

[ref306] Smith N. F.; Figg W. D.; Sparreboom A. Pharmacogenetics of Irinotecan Metabolism and Transport: An Update. Toxicol. In Vitro 2006, 20, 163–175. 10.1016/j.tiv.2005.06.045. 16271446

[ref307] Wallace B. D.; Wang H.; Lane K. T.; Scott J. E.; Orans J.; Koo J. S.; Venkatesh M.; Jobin C.; Yeh L.-A.; Mani S.; et al. Alleviating Cancer Drug Toxicity by Inhibiting a Bacterial Enzyme. Science 2010, 330, 831–835. 10.1126/science.1191175. 21051639 PMC3110694

[ref308] Wang Z.; Roberts A. B.; Buffa J. A.; Levison B. S.; Zhu W.; Org E.; Gu X.; Huang Y.; Zamanian-Daryoush M.; Culley M. K.; et al. Non-Lethal Inhibition of Gut Microbial Trimethylamine Production for the Treatment of Atherosclerosis. Cell 2015, 163, 1585–1595. 10.1016/j.cell.2015.11.055. 26687352 PMC4871610

[ref309] Roberts A. B.; Gu X.; Buffa J. A.; Hurd A. G.; Wang Z.; Zhu W.; Gupta N.; Skye S. M.; Cody D. B.; Levison B. S.; et al. Development of a Gut Microbe–Targeted Nonlethal Therapeutic to Inhibit Thrombosis Potential. Nat. Med. 2018, 24, 1407–1417. 10.1038/s41591-018-0128-1. 30082863 PMC6129214

[ref310] Craciun S.; Balskus E. P. Microbial Conversion of Choline to Trimethylamine Requires a Glycyl Radical Enzyme. Proc. Natl. Acad. Sci. U. S. A. 2012, 109, 21307–21312. 10.1073/pnas.1215689109. 23151509 PMC3535645

[ref311] Wang Z.; Klipfell E.; Bennett B. J.; Koeth R.; Levison B. S.; DuGar B.; Feldstein A. E.; Britt E. B.; Fu X.; Chung Y.-M.; et al. Gut Flora Metabolism of Phosphatidylcholine Promotes Cardiovascular Disease. Nature 2011, 472, 57–63. 10.1038/nature09922. 21475195 PMC3086762

[ref312] Isabella V. M.; Ha B. N.; Castillo M. J.; Lubkowicz D. J.; Rowe S. E.; Millet Y. A.; Anderson C. L.; Li N.; Fisher A. B.; West K. A.; et al. Development of a Synthetic Live Bacterial Therapeutic for the Human Metabolic Disease Phenylketonuria. Nat. Biotechnol. 2018, 36, 857 10.1038/nbt.4222. 30102294

[ref313] Bai L.; Gao M.; Cheng X.; Kang G.; Cao X.; Huang H. Engineered Butyrate-Producing Bacteria Prevents High Fat Diet-Induced Obesity in Mice. Microb. Cell Fact. 2020, 19, 94 10.1186/s12934-020-01350-z. 32334588 PMC7183672

[ref314] Lubkowicz D.; Horvath N. G.; James M. J.; Cantarella P.; Renaud L.; Bergeron C. G.; Shmueli R. B.; Anderson C.; Gao J.-R.; Kurtz C. B.; et al. An Engineered Bacterial Therapeutic Lowers Urinary Oxalate in Preclinical Models and in Silico Simulations of Enteric Hyperoxaluria. Mol. Syst. Biol. 2022, 18, e10539 10.15252/msb.202110539. 35253995 PMC8899768

[ref315] Ho C. L.; Tan H. Q.; Chua K. J.; Kang A.; Lim K. H.; Ling K. L.; Yew W. S.; Lee Y. S.; Thiery J. P.; Chang M. W. Engineered Commensal Microbes for Diet-Mediated Colorectal-Cancer Chemoprevention. Nat. Biomed. Eng. 2018, 2, 27–37. 10.1038/s41551-017-0181-y. 31015663

[ref316] Canale F. P.; Basso C.; Antonini G.; Perotti M.; Li N.; Sokolovska A.; Neumann J.; James M. J.; Geiger S.; Jin W.; et al. Metabolic Modulation of Tumours with Engineered Bacteria for Immunotherapy. Nature 2021, 598, 662–666. 10.1038/s41586-021-04003-2. 34616044

[ref317] Abedi M. H.; Yao M. S.; Mittelstein D. R.; Bar-Zion A.; Swift M. B.; Lee-Gosselin A.; Barturen-Larrea P.; Buss M. T.; Shapiro M. G. Ultrasound-Controllable Engineered Bacteria for Cancer Immunotherapy. Nat. Commun. 2022, 13, 1585 10.1038/s41467-022-29065-2. 35332124 PMC8948203

[ref318] López-Igual R.; Bernal-Bayard J.; Rodríguez-Patón A.; Ghigo J.-M.; Mazel D. Engineered Toxin–Intein Antimicrobials Can Selectively Target and Kill Antibiotic-Resistant Bacteria in Mixed Populations. Nat. Biotechnol. 2019, 37, 755–760. 10.1038/s41587-019-0105-3. 30988505

[ref319] Tscherner M.; Giessen T. W.; Markey L.; Kumamoto C. A.; Silver P. A. A Synthetic System That Senses Candida Albicans and Inhibits Virulence Factors. ACS Synth. Biol. 2019, 8, 434–444. 10.1021/acssynbio.8b00457. 30608638

[ref320] Cubillos-Ruiz A.; Alcantar M. A.; Donghia N. M.; Cárdenas P.; Avila-Pacheco J.; Collins J. J. An Engineered Live Biotherapeutic for the Prevention of Antibiotic-Induced Dysbiosis. Nat. Biomed. Eng. 2022, 6, 910–921. 10.1038/s41551-022-00871-9. 35411114

[ref321] Vågesjö E.; Öhnstedt E.; Mortier A.; Lofton H.; Huss F.; Proost P.; Roos S.; Phillipson M. Accelerated Wound Healing in Mice by on-Site Production and Delivery of Cxcl12 by Transformed Lactic Acid Bacteria. Proc. Natl. Acad. Sci. U. S. A. 2018, 115, 1895 10.1073/pnas.1716580115. 29432190 PMC5828606

[ref322] Praveschotinunt P.; Duraj-Thatte A. M.; Gelfat I.; Bahl F.; Chou D. B.; Joshi N. S. Engineered E. Coli Nissle 1917 for the Delivery of Matrix-Tethered Therapeutic Domains to the Gut. Nat. Commun. 2019, 10, 5580 10.1038/s41467-019-13336-6. 31811125 PMC6898321

[ref323] Aggarwal N.; Breedon A. M. E.; Davis C. M.; Hwang I. Y.; Chang M. W. Engineering Probiotics for Therapeutic Applications: Recent Examples and Translational Outlook. Curr. Opin. Biotechnol. 2020, 65, 171–179. 10.1016/j.copbio.2020.02.016. 32304955

[ref324] Zhou Z.; Chen X.; Sheng H.; Shen X.; Sun X.; Yan Y.; Wang J.; Yuan Q. Engineering Probiotics as Living Diagnostics and Therapeutics for Improving Human Health. Microb. Cell Fact. 2020, 19, 56 10.1186/s12934-020-01318-z. 32131831 PMC7055047

[ref325] Barra M.; Danino T.; Garrido D. Engineered Probiotics for Detection and Treatment of Inflammatory Intestinal Diseases. Front. Bioeng. Biotechnol. 2020, 8, 265 10.3389/fbioe.2020.00265. 32296696 PMC7137092

[ref326] Shen H.; Aggarwal N.; Wun K. S.; Lee Y. S.; Hwang I. Y.; Chang M. W. Engineered Microbial Systems for Advanced Drug Delivery. Adv. Drug Delivery Rev. 2022, 187, 114364 10.1016/j.addr.2022.114364. 35654214

[ref327] Visconti A.; Le Roy C. I.; Rosa F.; Rossi N.; Martin T. C.; Mohney R. P.; Li W.; de Rinaldis E.; Bell J. T.; Venter J. C.; et al. Interplay between the Human Gut Microbiome and Host Metabolism. Nat. Commun. 2019, 10, 4505 10.1038/s41467-019-12476-z. 31582752 PMC6776654

[ref328] Strong A.; Gold J.; Gold N. B.; Yudkoff M. Hepatic Manifestations of Urea Cycle Disorders. Clin. Liver Dis. 2021, 18, 198–203. 10.1002/cld.1115. PMC8549711 34745578

[ref329] Kurtz C. B.; Millet Y. A.; Puurunen M. K.; Perreault M.; Charbonneau M. R.; Isabella V. M.; Kotula J. W.; Antipov E.; Dagon Y.; Denney W. S.; et al. An Engineered E. Coli Nissle Improves Hyperammonemia and Survival in Mice and Shows Dose-Dependent Exposure in Healthy Humans. Sci. Transl. Med. 2019, 11, eaau7975 10.1126/scitranslmed.aau7975. 30651324

[ref330] Synlogic. Synlogic Discontinues Development of Synb1020 to Treat Hyperammonemia. https://investor.synlogictx.com/news-releases/news-release-details/synlogic-discontinues-development-synb1020-treat-hyperammonemia (accessed May 7, 2022).

[ref331] Silva Y. P.; Bernardi A.; Frozza R. L. The Role of Short-Chain Fatty Acids from Gut Microbiota in Gut-Brain Communication. Front. Endocrinol. 2020, 11, 25 10.3389/fendo.2020.00025. PMC7005631 32082260

[ref332] Li Z.; Yi C.-X.; Katiraei S.; Kooijman S.; Zhou E.; Chung C. K.; Gao Y.; van den Heuvel J. K.; Meijer O. C.; Berbée J. F. P.; et al. Butyrate Reduces Appetite and Activates Brown Adipose Tissue Via the Gut-Brain Neural Circuit. Gut 2018, 67, 1269 10.1136/gutjnl-2017-314050. 29101261

[ref333] Puddu A.; Sanguineti R.; Montecucco F.; Viviani G. L. Evidence for the Gut Microbiota Short-Chain Fatty Acids as Key Pathophysiological Molecules Improving Diabetes. Mediators Inflammation 2014, 2014, 162021 10.1155/2014/162021. PMC4151858 25214711

[ref334] Wang L.; Cheng X.; Bai L.; Gao M.; Kang G.; Cao X.; Huang H. Positive Interventional Effect of Engineered Butyrate-Producing Bacteria on Metabolic Disorders and Intestinal Flora Disruption in Obese Mice. Microbiol. Spectr. 2022, 10, e0114721 10.1128/spectrum.01147-21. 35293806 PMC9045090

[ref335] Bai Y.; Mansell T. J. Production and Sensing of Butyrate in a Probiotic E. Coli Strain. Int. J. Mol. Sci. 2020, 21, 3615 10.3390/ijms21103615. 32443851 PMC7279287

[ref336] Hwang I. Y.; Kim H. R.; De Sotto R.; Chang M. W. Engineered Probiotics Modulate the Endocannabinoid System. Biotechnology Notes 2021, 2, 33–38. 10.1016/j.biotno.2021.08.001.

[ref337] Scott B. M.; Gutiérrez-Vázquez C.; Sanmarco L. M.; da Silva Pereira J. A.; Li Z.; Plasencia A.; Hewson P.; Cox L. M.; O’Brien M.; Chen S. K.; et al. Self-Tunable Engineered Yeast Probiotics for the Treatment of Inflammatory Bowel Disease. Nat. Med. 2021, 27, 1212–1222. 10.1038/s41591-021-01390-x. 34183837

[ref338] Brenner K.; You L.; Arnold F. H. Engineering Microbial Consortia: A New Frontier in Synthetic Biology. Trends Biotechnol. 2008, 26, 483–489. 10.1016/j.tibtech.2008.05.004. 18675483

[ref339] Hanssen N. M. J.; de Vos W. M.; Nieuwdorp M. Fecal Microbiota Transplantation in Human Metabolic Diseases: From a Murky Past to a Bright Future?. Cell Metab. 2021, 33, 1098–1110. 10.1016/j.cmet.2021.05.005. 34077717

[ref340] Cammarota G.; Ianiro G.; Tilg H.; Rajilić-Stojanović M.; Kump P.; Satokari R.; Sokol H.; Arkkila P.; Pintus C.; Hart A.; et al. European Consensus Conference on Faecal Microbiota Transplantation in Clinical Practice. Gut 2017, 66, 569 10.1136/gutjnl-2016-313017. 28087657 PMC5529972

[ref341] van Nood E.; Vrieze A.; Nieuwdorp M.; Fuentes S.; Zoetendal E. G.; de Vos W. M.; Visser C. E.; Kuijper E. J.; Bartelsman J. F. W. M.; Tijssen J. G. P.; et al. Duodenal Infusion of Donor Feces for Recurrent Clostridium Difficile. N. Engl. J. Med. 2013, 368, 407–415. 10.1056/NEJMoa1205037. 23323867

[ref342] Suez J.; Zmora N.; Zilberman-Schapira G.; Mor U.; Dori-Bachash M.; Bashiardes S.; Zur M.; Regev-Lehavi D.; Ben-Zeev Brik R.; Federici S.; et al. Post-Antibiotic Gut Mucosal Microbiome Reconstitution Is Impaired by Probiotics and Improved by Autologous Fmt. Cell 2018, 174, 1406–1423. 10.1016/j.cell.2018.08.047. 30193113

[ref343] Costello S. P.; Hughes P. A.; Waters O.; Bryant R. V.; Vincent A. D.; Blatchford P.; Katsikeros R.; Makanyanga J.; Campaniello M. A.; Mavrangelos C.; et al. Effect of Fecal Microbiota Transplantation on 8-Week Remission in Patients with Ulcerative Colitis: A Randomized Clinical Trial. JAMA 2019, 321, 156–164. 10.1001/jama.2018.20046. 30644982 PMC6439766

[ref344] Shen Z.-H.; Zhu C.-X.; Quan Y.-S.; Yang Z.-Y.; Wu S.; Luo W.-W.; Tan B.; Wang X.-Y. Relationship between Intestinal Microbiota and Ulcerative Colitis: Mechanisms and Clinical Application of Probiotics and Fecal Microbiota Transplantation. World J. Gastroenterol. 2018, 24, 5–14. 10.3748/wjg.v24.i1.5. 29358877 PMC5757125

[ref345] Ng S. C.; Xu Z.; Mak J. W. Y.; Yang K.; Liu Q.; Zuo T.; Tang W.; Lau L.; Lui R. N.; Wong S. H. Microbiota Engraftment after Faecal Microbiota Transplantation in Obese Subjects with Type 2 Diabetes: A 24-Week, Double-Blind, Randomised Controlled Trial. Gut 2022, 71, 716–723. 10.1136/gutjnl-2020-323617. 33785557

[ref346] Xu D.; Chen V. L.; Steiner C. A.; Berinstein J. A.; Eswaran S.; Waljee A. K.; Higgins P. D. R.; Owyang C. Efficacy of Fecal Microbiota Transplantation in Irritable Bowel Syndrome: A Systematic Review and Meta-Analysis. Am. J. Gastroenterol. 2019, 114, 1043–1050. 10.14309/ajg.0000000000000198. 30908299 PMC7257434

[ref347] Chen D.; Wu J.; Jin D.; Wang B.; Cao H. Fecal Microbiota Transplantation in Cancer Management: Current Status and Perspectives. Int. J. Cancer 2019, 145, 2021–2031. 10.1002/ijc.32003. 30458058 PMC6767494

[ref348] Park S.-K.; Lee C.-W.; Park D.-I.; Woo H.-Y.; Cheong H. S.; Shin H. C.; Ahn K.; Kwon M.-J.; Joo E.-J. Detection of Sars-Cov-2 in Fecal Samples from Patients with Asymptomatic and Mild Covid-19 in Korea. Clin. Gastroenterol. Hepatol. 2021, 19, 1387–1394. 10.1016/j.cgh.2020.06.005. 32534042 PMC7286243

[ref349] Wilson B. C.; Vatanen T.; Cutfield W. S.; O’Sullivan J. M. The Super-Donor Phenomenon in Fecal Microbiota Transplantation. Front. Cell. Infect. Microbiol. 2019, 9, 2 10.3389/fcimb.2019.00002. 30719428 PMC6348388

[ref350] McGovern B. H.; Ford C. B.; Henn M. R.; Pardi D. S.; Khanna S.; Hohmann E. L.; O’Brien E. J.; Desjardins C. A.; Bernardo P.; Wortman J. R.; et al. Ser-109, an Investigational Microbiome Drug to Reduce Recurrence after Clostridioides Difficile Infection: Lessons Learned from a Phase 2 Trial. Clin. Infect. Dis. 2021, 72, 2132–2140. 10.1093/cid/ciaa387. 32255488 PMC8204772

[ref351] Weingarden A. R.; Dosa P. I.; DeWinter E.; Steer C. J.; Shaughnessy M. K.; Johnson J. R.; Khoruts A.; Sadowsky M. J. Changes in Colonic Bile Acid Composition Following Fecal Microbiota Transplantation Are Sufficient to Control Clostridium Difficile Germination and Growth. PLoS One 2016, 11, e0147210 10.1371/journal.pone.0147210. 26789728 PMC4720481

[ref352] Feuerstadt P.; Louie T. J.; Lashner B.; Wang E. E. L.; Diao L.; Bryant J. A.; Sims M.; Kraft C. S.; Cohen S. H.; Berenson C. S.; et al. Ser-109, an Oral Microbiome Therapy for Recurrent Clostridioides Difficile Infection. N. Engl. J. Med. 2022, 386, 220–229. 10.1056/NEJMoa2106516. 35045228

[ref353] Tanoue T.; Morita S.; Plichta D. R.; Skelly A. N.; Suda W.; Sugiura Y.; Narushima S.; Vlamakis H.; Motoo I.; Sugita K.; et al. A Defined Commensal Consortium Elicits Cd8 T Cells and Anti-Cancer Immunity. Nature 2019, 565, 600–605. 10.1038/s41586-019-0878-z. 30675064

[ref354] Hofer U. The Next Step Towards Anticancer Microbiota Therapeutics. Nature Rev. Microbiol. 2019, 17, 125–125. 10.1038/s41579-019-0152-2. 30705418

[ref355] van der Lelie D.; Oka A.; Taghavi S.; Umeno J.; Fan T.-J.; Merrell K. E.; Watson S. D.; Ouellette L.; Liu B.; Awoniyi M.; et al. Rationally Designed Bacterial Consortia to Treat Chronic Immune-Mediated Colitis and Restore Intestinal Homeostasis. Nat. Commun. 2021, 12, 3105 10.1038/s41467-021-23460-x. 34050144 PMC8163890

[ref356] Lloyd-Price J.; Arze C.; Ananthakrishnan A. N.; Schirmer M.; Avila-Pacheco J.; Poon T. W.; Andrews E.; Ajami N. J.; Bonham K. S.; Brislawn C. J.; et al. Multi-Omics of the Gut Microbial Ecosystem in Inflammatory Bowel Diseases. Nature 2019, 569, 655–662. 10.1038/s41586-019-1237-9. 31142855 PMC6650278

[ref357] Alexeev E. E.; Lanis J. M.; Kao D. J.; Campbell E. L.; Kelly C. J.; Battista K. D.; Gerich M. E.; Jenkins B. R.; Walk S. T.; Kominsky D. J.; et al. Microbiota-Derived Indole Metabolites Promote Human and Murine Intestinal Homeostasis through Regulation of Interleukin-10 Receptor. Am. J. Pathol. 2018, 188, 1183–1194. 10.1016/j.ajpath.2018.01.011. 29454749 PMC5906738

[ref358] Kong W.; Meldgin D. R.; Collins J. J.; Lu T. Designing Microbial Consortia with Defined Social Interactions. Nature Chem. Biol. 2018, 14, 821–829. 10.1038/s41589-018-0091-7. 29942078

[ref359] Balagaddé F. K.; Song H.; Ozaki J.; Collins C. H.; Barnet M.; Arnold F. H.; Quake S. R.; You L. A Synthetic Escherichia Coli Predator–Prey Ecosystem. Mol. Syst. Biol. 2008, 4, 187 10.1038/msb.2008.24. 18414488 PMC2387235

[ref360] Lindemann S. R.; Bernstein H. C.; Song H.-S.; Fredrickson J. K.; Fields M. W.; Shou W.; Johnson D. R.; Beliaev A. S. Engineering Microbial Consortia for Controllable Outputs. ISME J. 2016, 10, 2077–2084. 10.1038/ismej.2016.26. 26967105 PMC4989317

[ref361] Lloyd-Price J.; Mahurkar A.; Rahnavard G.; Crabtree J.; Orvis J.; Hall A. B.; Brady A.; Creasy H. H.; McCracken C.; Giglio M. G.; et al. Strains, Functions and Dynamics in the Expanded Human Microbiome Project. Nature 2017, 550, 61–66. 10.1038/nature23889. 28953883 PMC5831082

[ref362] The Integrative Human Microbiome Project: Dynamic Analysis of Microbiome-Host Omics Profiles During Periods of Human Health and Disease. Cell Host Microbe 2014, 16, 276–289. 10.1016/j.chom.2014.08.014. 25211071 PMC5109542

[ref363] Mark Welch J. L.; Hasegawa Y.; McNulty N. P.; Gordon J. I.; Borisy G. G. Spatial Organization of a Model 15-Member Human Gut Microbiota Established in Gnotobiotic Mice. Proc. Natl. Acad. Sci. U. S. A. 2017, 114, E9105–E9114. 10.1073/pnas.1711596114. 29073107 PMC5664539

[ref364] Cao X.; Hamilton J. J.; Venturelli O. S. Understanding and Engineering Distributed Biochemical Pathways in Microbial Communities. Biochem. 2019, 58, 94–107. 10.1021/acs.biochem.8b01006. 30457843 PMC6733022

[ref365] Sheth R. U.; Li M.; Jiang W.; Sims P. A.; Leong K. W.; Wang H. H. Spatial Metagenomic Characterization of Microbial Biogeography in the Gut. Nat. Biotechnol. 2019, 37, 877–883. 10.1038/s41587-019-0183-2. 31332325 PMC6679743

[ref366] Zhao S.; Lieberman T. D.; Poyet M.; Kauffman K. M.; Gibbons S. M.; Groussin M.; Xavier R. J.; Alm E. J. Adaptive Evolution within Gut Microbiomes of Healthy People. Cell Host Microbe 2019, 25, 656–667. 10.1016/j.chom.2019.03.007. 31028005 PMC6749991

[ref367] Guo C.-J.; Allen B. M.; Hiam K. J.; Dodd D.; Van Treuren W.; Higginbottom S.; Nagashima K.; Fischer C. R.; Sonnenburg J. L.; Spitzer M. H.; et al. Depletion of Microbiome-Derived Molecules in the Host Using Clostridium Genetics. Science 2019, 366, eaav1282 10.1126/science.aav1282. 31831639 PMC7141153

[ref368] Backhed F.; Manchester J. K.; Semenkovich C. F.; Gordon J. I. Mechanisms Underlying the Resistance to Diet-Induced Obesity in Germ-Free Mice. Proc. Natl. Acad. Sci. U. S. A. 2007, 104, 979–984. 10.1073/pnas.0605374104. 17210919 PMC1764762

[ref369] Turnbaugh P. J.; Ley R. E.; Mahowald M. A.; Magrini V.; Mardis E. R.; Gordon J. I. An Obesity-Associated Gut Microbiome with Increased Capacity for Energy Harvest. Nature 2006, 444, 1027–1031. 10.1038/nature05414. 17183312

[ref370] Ridaura V. K.; Faith J. J.; Rey F. E.; Cheng J.; Duncan A. E.; Kau A. L.; Griffin N. W.; Lombard V.; Henrissat B.; Bain J. R.; et al. Gut Microbiota from Twins Discordant for Obesity Modulate Metabolism in Mice. Science 2013, 341, 1241214 10.1126/science.1241214. 24009397 PMC3829625

[ref371] Alang N.; Kelly C. R. Weight Gain after Fecal Microbiota Transplantation. Open Forum Infect. Dis. 2015, 2, ofv004 10.1093/ofid/ofv004. 26034755 PMC4438885

[ref372] Donia M. S.; Fischbach M. A. Small Molecules from the Human Microbiota. Science 2015, 349, 1254766 10.1126/science.1254766. 26206939 PMC4641445

[ref373] Smith P. M.; Howitt M. R.; Panikov N.; Michaud M.; Gallini C. A.; Bohlooly-Y Y. M.; Glickman J. N.; Garrett W. S. The Microbial Metabolites, Short-Chain Fatty Acids, Regulate Colonic Treg Cell Homeostasis. Science 2013, 341, 569–573. 10.1126/science.1241165. 23828891 PMC3807819

[ref374] Arpaia N.; Campbell C.; Fan X.; Dikiy S.; van der Veeken J.; deRoos P.; Liu H.; Cross J. R.; Pfeffer K.; Coffer P. J.; et al. Metabolites Produced by Commensal Bacteria Promote Peripheral Regulatory T-Cell Generation. Nature 2013, 504, 451–455. 10.1038/nature12726. 24226773 PMC3869884

[ref375] O’Connell T. M. The Application of Metabolomics to Probiotic and Prebiotic Interventions in Human Clinical Studies. Metabolites 2020, 10, 120 10.3390/metabo10030120. 32213886 PMC7143099

[ref376] Han S.; Van Treuren W.; Fischer C. R.; Merrill B. D.; DeFelice B. C.; Sanchez J. M.; Higginbottom S. K.; Guthrie L.; Fall L. A.; Dodd D.; et al. A Metabolomics Pipeline for the Mechanistic Interrogation of the Gut Microbiome. Nature 2021, 595, 415–420. 10.1038/s41586-021-03707-9. 34262212 PMC8939302

[ref377] Koh A.; Molinaro A.; Stahlman M.; Khan M. T.; Schmidt C.; Manneras-Holm L.; Wu H.; Carreras A.; Jeong H.; Olofsson L. E.; et al. Microbially Produced Imidazole Propionate Impairs Insulin Signaling through Mtorc1. Cell 2018, 175, 947–961. 10.1016/j.cell.2018.09.055. 30401435

[ref378] Nemet I.; Saha P. P.; Gupta N.; Zhu W.; Romano K. A.; Skye S. M.; Cajka T.; Mohan M. L.; Li L.; Wu Y.; et al. A Cardiovascular Disease-Linked Gut Microbial Metabolite Acts Via Adrenergic Receptors. Cell 2020, 180, 862–877. 10.1016/j.cell.2020.02.016. 32142679 PMC7402401

[ref379] Peisl B. Y. L.; Schymanski E. L.; Wilmes P. Dark Matter in Host-Microbiome Metabolomics: Tackling the Unknowns-a Review. Anal. Chim. Acta 2018, 1037, 13–27. 10.1016/j.aca.2017.12.034. 30292286

[ref380] Greener J. G.; Kandathil S. M.; Moffat L.; Jones D. T. A Guide to Machine Learning for Biologists. Nat. Rev. Mol. Cell. Biol. 2022, 23, 40–55. 10.1038/s41580-021-00407-0. 34518686

[ref381] Pomyen Y.; Wanichthanarak K.; Poungsombat P.; Fahrmann J.; Grapov D.; Khoomrung S. Deep Metabolome: Applications of Deep Learning in Metabolomics. Comput. Struct. Biotechnol. J. 2020, 18, 2818–2825. 10.1016/j.csbj.2020.09.033. 33133423 PMC7575644

[ref382] Colby S. M.; Nunez J. R.; Hodas N. O.; Corley C. D.; Renslow R. R. Deep Learning to Generate in Silico Chemical Property Libraries and Candidate Molecules for Small Molecule Identification in Complex Samples. Anal. Chem. 2020, 92, 1720–1729. 10.1021/acs.analchem.9b02348. 31661259

[ref383] Shakya M.; Lo C. C.; Chain P. S. G. Advances and Challenges in Metatranscriptomic Analysis. Front. Genet. 2019, 10, 904 10.3389/fgene.2019.00904. 31608125 PMC6774269

[ref384] Berg C.; Dupont C. L.; Asplund-Samuelsson J.; Celepli N. A.; Eiler A.; Allen A. E.; Ekman M.; Bergman B.; Ininbergs K. Dissection of Microbial Community Functions During a Cyanobacterial Bloom in the Baltic Sea Via Metatranscriptomics. Front. Mar. Sci. 2018, 5, 55 10.3389/fmars.2018.00055.

[ref385] Moniruzzaman M.; Wurch L. L.; Alexander H.; Dyhrman S. T.; Gobler C. J.; Wilhelm S. W. Virus-Host Relationships of Marine Single-Celled Eukaryotes Resolved from Metatranscriptomics. Nat. Commun. 2017, 8, 16054 10.1038/ncomms16054. 28656958 PMC5493757

[ref386] White R. A. 3rd; Bottos E. M.; Roy Chowdhury T.; Zucker J. D.; Brislawn C. J.; Nicora C. D.; Fansler S. J.; Glaesemann K. R.; Glass K.; Jansson J. K. Moleculo Long-Read Sequencing Facilitates Assembly and Genomic Binning from Complex Soil Metagenomes. mSystems 2016, 1, e00045-16 10.1128/mSystems.00045-16. 27822530 PMC5069762

[ref387] Damon C.; Lehembre F.; Oger-Desfeux C.; Luis P.; Ranger J.; Fraissinet-Tachet L.; Marmeisse R. Metatranscriptomics Reveals the Diversity of Genes Expressed by Eukaryotes in Forest Soils. PLoS One 2012, 7, e28967 10.1371/journal.pone.0028967. 22238585 PMC3253082

[ref388] Franzosa E. A.; Morgan X. C.; Segata N.; Waldron L.; Reyes J.; Earl A. M.; Giannoukos G.; Boylan M. R.; Ciulla D.; Gevers D.; et al. Relating the Metatranscriptome and Metagenome of the Human Gut. Proc. Natl. Acad. Sci. U. S. A. 2014, 111, E2329–2338. 10.1073/pnas.1319284111. 24843156 PMC4050606

[ref389] Nowicki E. M.; Shroff R.; Singleton J. A.; Renaud D. E.; Wallace D.; Drury J.; Zirnheld J.; Colleti B.; Ellington A. D.; Lamont R. J.; et al. Microbiota and Metatranscriptome Changes Accompanying the Onset of Gingivitis. mBio 2018, 9, e00575-18 10.1128/mBio.00575-18. 29666288 PMC5904416

[ref390] Schirmer M.; Franzosa E. A.; Lloyd-Price J.; McIver L. J.; Schwager R.; Poon T. W.; Ananthakrishnan A. N.; Andrews E.; Barron G.; Lake K.; et al. Dynamics of Metatranscription in the Inflammatory Bowel Disease Gut Microbiome. Nat. Microbiol. 2018, 3, 337–346. 10.1038/s41564-017-0089-z. 29311644 PMC6131705

[ref391] Wilmes P.; Bond P. L. Metaproteomics: Studying Functional Gene Expression in Microbial Ecosystems. Trends Microbiol. 2006, 14, 92–97. 10.1016/j.tim.2005.12.006. 16406790

[ref392] Verberkmoes N. C.; Russell A. L.; Shah M.; Godzik A.; Rosenquist M.; Halfvarson J.; Lefsrud M. G.; Apajalahti J.; Tysk C.; Hettich R. L.; et al. Shotgun Metaproteomics of the Human Distal Gut Microbiota. ISME J. 2009, 3, 179–189. 10.1038/ismej.2008.108. 18971961

[ref393] Tanca A.; Abbondio M.; Palomba A.; Fraumene C.; Manghina V.; Cucca F.; Fiorillo E.; Uzzau S. Potential and Active Functions in the Gut Microbiota of a Healthy Human Cohort. Microbiome 2017, 5, 79 10.1186/s40168-017-0293-3. 28709472 PMC5513205

[ref394] Long S.; Yang Y.; Shen C.; Wang Y.; Deng A.; Qin Q.; Qiao L. Metaproteomics Characterizes Human Gut Microbiome Function in Colorectal Cancer. npj Biofilms Microbiomes 2020, 6, 14 10.1038/s41522-020-0123-4. 32210237 PMC7093434

[ref395] Issa Isaac N.; Philippe D.; Nicholas A.; Raoult D.; Eric C. Metaproteomics of the Human Gut Microbiota: Challenges and Contributions to Other Omics. Clin. Mass. Spectrom. 2019, 14, 18–30. 10.1016/j.clinms.2019.06.001. 34917758 PMC8669434

[ref396] Breton J.; Legrand R.; Achamrah N.; Chan P.; do Rego J. L.; do Rego J. C.; Coeffier M.; Dechelotte P.; Fetissov S. O. Proteome Modifications of Gut Microbiota in Mice with Activity-Based Anorexia and Starvation: Role in Atp Production. Nutrition 2019, 67–68, 110557 10.1016/j.nut.2019.110557. 31563744

[ref397] Lobel L.; Cao Y. G.; Fenn K.; Glickman J. N.; Garrett W. S. Diet Posttranslationally Modifies the Mouse Gut Microbial Proteome to Modulate Renal Function. Science 2020, 369, 1518–1524. 10.1126/science.abb3763. 32943527 PMC8178816

[ref398] Zhang X.; Chen W.; Ning Z.; Mayne J.; Mack D.; Stintzi A.; Tian R.; Figeys D. Deep Metaproteomics Approach for the Study of Human Microbiomes. Anal. Chem. 2017, 89, 9407–9415. 10.1021/acs.analchem.7b02224. 28749657

[ref399] Zhang X.; Deeke S. A.; Ning Z.; Starr A. E.; Butcher J.; Li J.; Mayne J.; Cheng K.; Liao B.; Li L.; et al. Metaproteomics Reveals Associations between Microbiome and Intestinal Extracellular Vesicle Proteins in Pediatric Inflammatory Bowel Disease. Nat. Commun. 2018, 9, 2873 10.1038/s41467-018-05357-4. 30030445 PMC6054643

[ref400] Zhang X.; Ning Z.; Mayne J.; Yang Y.; Deeke S. A.; Walker K.; Farnsworth C. L.; Stokes M. P.; Couture J.-F.; Mack D.; et al. Widespread Protein Lysine Acetylation in Gut Microbiome and Its Alterations in Patients with Crohn’s Disease. Nat. Commun. 2020, 11, 4120 10.1038/s41467-020-17916-9. 32807798 PMC7431864

[ref401] Lloyd-Price J.; Arze C.; Ananthakrishnan A. N.; Schirmer M.; Avila-Pacheco J.; Poon T. W.; Andrews E.; Ajami N. J.; Bonham K. S.; Brislawn C. J.; et al. Multi-Omics of the Gut Microbial Ecosystem in Inflammatory Bowel Diseases. Nature 2019, 569, 655–662. 10.1038/s41586-019-1237-9. 31142855 PMC6650278

[ref402] Mills R. H.; Dulai P. S.; Vazquez-Baeza Y.; Sauceda C.; Daniel N.; Gerner R. R.; Batachari L. E.; Malfavon M.; Zhu Q.; Weldon K.; et al. Multi-Omics Analyses of the Ulcerative Colitis Gut Microbiome Link Bacteroides Vulgatus Proteases with Disease Severity. Nat. Microbiol. 2022, 7, 262–276. 10.1038/s41564-021-01050-3. 35087228 PMC8852248

[ref403] Rothschild D.; Weissbrod O.; Barkan E.; Kurilshikov A.; Korem T.; Zeevi D.; Costea P. I.; Godneva A.; Kalka I. N.; Bar N.; et al. Environment Dominates over Host Genetics in Shaping Human Gut Microbiota. Nature 2018, 555, 210–215. 10.1038/nature25973. 29489753

[ref404] Goodrich J. K.; Davenport E. R.; Beaumont M.; Jackson M. A.; Knight R.; Ober C.; Spector T. D.; Bell J. T.; Clark A. G.; Ley R. E. Genetic Determinants of the Gut Microbiome in Uk Twins. Cell Host Microbe 2016, 19, 731–743. 10.1016/j.chom.2016.04.017. 27173935 PMC4915943

[ref405] Goodrich J. K.; Waters J. L.; Poole A. C.; Sutter J. L.; Koren O.; Blekhman R.; Beaumont M.; Van Treuren W.; Knight R.; Bell J. T.; et al. Human Genetics Shape the Gut Microbiome. Cell 2014, 159, 789–799. 10.1016/j.cell.2014.09.053. 25417156 PMC4255478

[ref406] Turpin W.; Espin-Garcia O.; Xu W.; Silverberg M. S.; Kevans D.; Smith M. I.; Guttman D. S.; Griffiths A.; Panaccione R.; Otley A.; et al. Association of Host Genome with Intestinal Microbial Composition in a Large Healthy Cohort. Nat. Genet. 2016, 48, 1413–1417. 10.1038/ng.3693. 27694960

[ref407] Hove H.; Norgaard H.; Brøbech Mortensen P. Lactic Acid Bacteria and the Human Gastrointestinal Tract. Eur. J. Clin. Nutr. 1999, 53, 339–350. 10.1038/sj.ejcn.1600773. 10369488

[ref408] Hamaoui D.; Subtil A. Atg16l1 Functions in Cell Homeostasis Beyond Autophagy. FEBS J. 2022, 289, 1779–1800. 10.1111/febs.15833. 33752267

[ref409] Hampe J.; Franke A.; Rosenstiel P.; Till A.; Teuber M.; Huse K.; Albrecht M.; Mayr G.; De La Vega F. M.; Briggs J.; et al. A Genome-Wide Association Scan of Nonsynonymous Snps Identifies a Susceptibility Variant for Crohn Disease in Atg16l1. Nat. Genet. 2007, 39, 207–211. 10.1038/ng1954. 17200669

[ref410] Rioux J. D.; Xavier R. J.; Taylor K. D.; Silverberg M. S.; Goyette P.; Huett A.; Green T.; Kuballa P.; Barmada M. M.; Datta L. W.; et al. Genome-Wide Association Study Identifies New Susceptibility Loci for Crohn Disease and Implicates Autophagy in Disease Pathogenesis. Nat. Genet. 2007, 39, 596–604. 10.1038/ng2032. 17435756 PMC2757939

[ref411] Chu H.; Khosravi A.; Kusumawardhani I. P.; Kwon A. H.; Vasconcelos A. C.; Cunha L. D.; Mayer A. E.; Shen Y.; Wu W. L.; Kambal A.; et al. Gene-Microbiota Interactions Contribute to the Pathogenesis of Inflammatory Bowel Disease. Science 2016, 352, 1116–1120. 10.1126/science.aad9948. 27230380 PMC4996125

[ref412] Endy D. Foundations for Engineering Biology. Nature 2005, 438, 449–453. 10.1038/nature04342. 16306983

[ref413] Moe-Behrens G. H. The Biological Microprocessor, or How to Build a Computer with Biological Parts. Comput. Struct. Biotechnol. J. 2013, 7, e201304003 10.5936/csbj.201304003. 24688733 PMC3962179

[ref414] Singh V. Recent Advances and Opportunities in Synthetic Logic Gates Engineering in Living Cells. Syst. Synth. Biol. 2014, 8, 271–282. 10.1007/s11693-014-9154-6. 26396651 PMC4571725

[ref415] Elowitz M. B.; Leibler S. A Synthetic Oscillatory Network of Transcriptional Regulators. Nature 2000, 403, 335–338. 10.1038/35002125. 10659856

[ref416] Gardner T. S.; Cantor C. R.; Collins J. J. Construction of a Genetic Toggle Switch in Escherichia Coli. Nature 2000, 403, 339–342. 10.1038/35002131. 10659857

[ref417] Taketani M.; Zhang J.; Zhang S.; Triassi A. J.; Huang Y. J.; Griffith L. G.; Voigt C. A. Genetic Circuit Design Automation for the Gut Resident Species Bacteroides Thetaiotaomicron. Nat. Biotechnol. 2020, 38, 962–969. 10.1038/s41587-020-0468-5. 32231334 PMC8922546

[ref418] Merk L. N.; Shur A. S.; Pandey A.; Murray R. M.; Green L. N. Engineering Logical Inflammation Sensing Circuit for Gut Modulation. bioRxiv 2020, 10.1101/2020.11.10.377085.

[ref419] Potvin-Trottier L.; Lord N. D.; Vinnicombe G.; Paulsson J. Synchronous Long-Term Oscillations in a Synthetic Gene Circuit. Nature 2016, 538, 514–517. 10.1038/nature19841. 27732583 PMC5637407

[ref420] Riglar D. T.; Giessen T. W.; Baym M.; Kerns S. J.; Niederhuber M. J.; Bronson R. T.; Kotula J. W.; Gerber G. K.; Way J. C.; Silver P. A. Engineered Bacteria Can Function in the Mammalian Gut Long-Term as Live Diagnostics of Inflammation. Nat. Biotechnol. 2017, 35, 653–658. 10.1038/nbt.3879. 28553941 PMC5658125

[ref421] Del Valle I.; Fulk E. M.; Kalvapalle P.; Silberg J. J.; Masiello C. A.; Stadler L. B. Translating New Synthetic Biology Advances for Biosensing into the Earth and Environmental Sciences. Front. Microbiol. 2021, 11, 618373 10.3389/fmicb.2020.618373. 33633695 PMC7901896

[ref422] Ceroni F.; Algar R.; Stan G. B.; Ellis T. Quantifying Cellular Capacity Identifies Gene Expression Designs with Reduced Burden. Nat. Methods. 2015, 12, 415–418. 10.1038/nmeth.3339. 25849635

[ref423] Ceroni F.; Boo A.; Furini S.; Gorochowski T. E.; Borkowski O.; Ladak Y. N.; Awan A. R.; Gilbert C.; Stan G. B.; Ellis T. Burden-Driven Feedback Control of Gene Expression. Nat. Methods. 2018, 15, 387–393. 10.1038/nmeth.4635. 29578536

[ref424] He L.; Yang H.; Tang J.; Liu Z.; Chen Y.; Lu B.; He H.; Tang S.; Sun Y.; Liu F.; et al. Intestinal Probiotics E. Coli Nissle 1917 as a Targeted Vehicle for Delivery of P53 and Tum-5 to Solid Tumors for Cancer Therapy. J. Biol. Eng. 2019, 13, 58 10.1186/s13036-019-0189-9. 31297149 PMC6599283

[ref425] He L.; Yang H.; Liu F.; Chen Y.; Tang S.; Ji W.; Tang J.; Liu Z.; Sun Y.; Hu S.; et al. Escherichia Coli Nissle 1917 Engineered to Express Tum-5 Can Restrain Murine Melanoma Growth. Oncotarget 2017, 8, 85772–85782. 10.18632/oncotarget.20486. 29156755 PMC5689645

[ref426] Sieow B. F.; Wun K. S.; Yong W. P.; Hwang I. Y.; Chang M. W. Tweak to Treat: Reprograming Bacteria for Cancer Treatment. Trends Cancer 2021, 7, 447–464. 10.1016/j.trecan.2020.11.004. 33303401

[ref427] Meyer A. J.; Segall-Shapiro T. H.; Glassey E.; Zhang J.; Voigt C. A. Escherichia Coli ″Marionette″ Strains with 12 Highly Optimized Small-Molecule Sensors. Nat. Chem. Biol. 2019, 15, 196–204. 10.1038/s41589-018-0168-3. 30478458

[ref428] Rottinghaus A. G.; Xi C.; Amrofell M. B.; Yi H.; Moon T. S. Engineering Ligand-Specific Biosensors for Aromatic Amino Acids and Neurochemicals. Cell Syst. 2022, 13, 204–214. 10.1016/j.cels.2021.10.006. 34767760 PMC8930536

[ref429] Wang L.; Walker B. L.; Iannaccone S.; Bhatt D.; Kennedy P. J.; Tse W. T. Bistable Switches Control Memory and Plasticity in Cellular Differentiation. Proc. Natl. Acad. Sci. U. S. A. 2009, 106, 6638–6643. 10.1073/pnas.0806137106. 19366677 PMC2672527

[ref430] Dean C. What Holds Epigenetic Memory?. Nat. Rev. Mol. Cell. Biol. 2017, 18, 140 10.1038/nrm.2017.15. 28220047

[ref431] Boraschi D.; Italiani P. Innate Immune Memory: Time for Adopting a Correct Terminology. Front. Immunol. 2018, 9, 799 10.3389/fimmu.2018.00799. 29725331 PMC5917086

[ref432] Inniss M. C.; Silver P. A. Building Synthetic Memory. Curr. Biol. 2013, 23, R812–816. 10.1016/j.cub.2013.06.047. 24028965 PMC3821973

[ref433] Yang L.; Nielsen A. A.; Fernandez-Rodriguez J.; McClune C. J.; Laub M. T.; Lu T. K.; Voigt C. A. Permanent Genetic Memory with > 1-Byte Capacity. Nat. Methods 2014, 11, 1261–1266. 10.1038/nmeth.3147. 25344638 PMC4245323

[ref434] Deans T. L.; Cantor C. R.; Collins J. J. A Tunable Genetic Switch Based on Rnai and Repressor Proteins for Regulating Gene Expression in Mammalian Cells. Cell 2007, 130, 363–372. 10.1016/j.cell.2007.05.045. 17662949

[ref435] O’Gorman S.; Fox D. T.; Wahl G. M. Recombinase-Mediated Gene Activation and Site-Specific Integration in Mammalian Cells. Science 1991, 251, 1351–1355. 10.1126/science.1900642. 1900642

[ref436] Kim H.; Kim M.; Im S. K.; Fang S. Mouse Cre-Loxp System: General Principles to Determine Tissue-Specific Roles of Target Genes. Lab. Anim. Res. 2018, 34, 147–159. 10.5625/lar.2018.34.4.147. 30671100 PMC6333611

[ref437] He L.; Li Y.; Li Y.; Pu W.; Huang X.; Tian X.; Wang Y.; Zhang H.; Liu Q.; Zhang L.; et al. Enhancing the Precision of Genetic Lineage Tracing Using Dual Recombinases. Nat. Med. 2017, 23, 1488–1498. 10.1038/nm.4437. 29131159 PMC6913096

[ref438] Kotula J. W.; Kerns S. J.; Shaket L. A.; Siraj L.; Collins J. J.; Way J. C.; Silver P. A. Programmable Bacteria Detect and Record an Environmental Signal in the Mammalian Gut. Proc. Natl. Acad. Sci. U. S. A. 2014, 111, 4838–4843. 10.1073/pnas.1321321111. 24639514 PMC3977281

[ref439] Naydich A. D.; Nangle S. N.; Bues J. J.; Trivedi D.; Nissar N.; Inniss M. C.; Niederhuber M. J.; Way J. C.; Silver P. A.; Riglar D. T. Synthetic Gene Circuits Enable Systems-Level Biosensor Trigger Discovery at the Host-Microbe Interface. mSystems 2019, 4, e00125-19 10.1128/mSystems.00125-19. 31186335 PMC6561318

[ref440] Lee J. W.; Chan C. T. Y.; Slomovic S.; Collins J. J. Next-Generation Biocontainment Systems for Engineered Organisms. Nat. Chem. Biol. 2018, 14, 530–537. 10.1038/s41589-018-0056-x. 29769737

[ref441] Molin S.; Klemm P.; Poulsen L. K.; Biehl H.; Gerdes K.; Andersson P. Conditional Suicide System for Containment of Bacteria and Plasmids. Bio/Technology 1987, 5, 1315–1318. 10.1038/nbt1287-1315.

[ref442] Contreras A.; Molin S.; Ramos J. L. Conditional-Suicide Containment System for Bacteria Which Mineralize Aromatics. Appl. Environ. Microbiol. 1991, 57, 1504–1508. 10.1128/aem.57.5.1504-1508.1991. 16348490 PMC182976

[ref443] Chan C. T.; Lee J. W.; Cameron D. E.; Bashor C. J.; Collins J. J. Deadman’ and ’Passcode’ Microbial Kill Switches for Bacterial Containment. Nat. Chem. Biol. 2016, 12, 82–86. 10.1038/nchembio.1979. 26641934 PMC4718764

[ref444] Meinhardt S.; Swint-Kruse L. Experimental Identification of Specificity Determinants in the Domain Linker of a Laci/Galr Protein: Bioinformatics-Based Predictions Generate True Positives and False Negatives. Proteins 2008, 73, 941–957. 10.1002/prot.22121. 18536016 PMC2585155

[ref445] Meinhardt S.; Manley M. W. Jr; Becker N. A.; Hessman J. A.; Maher L. J. 3rd; Swint-Kruse L. Novel Insights from Hybrid Laci/Galr Proteins: Family-Wide Functional Attributes and Biologically Significant Variation in Transcription Repression. Nucleic Acids Res. 2012, 40, 11139–11154. 10.1093/nar/gks806. 22965134 PMC3505978

[ref446] Chavez A.; Pruitt B. W.; Tuttle M.; Shapiro R. S.; Cecchi R. J.; Winston J.; Turczyk B. M.; Tung M.; Collins J. J.; Church G. M. Precise Cas9 Targeting Enables Genomic Mutation Prevention. Proc. Natl. Acad. Sci. U. S. A. 2018, 115, 3669–3673. 10.1073/pnas.1718148115. 29555762 PMC5889643

[ref447] Rottinghaus A. G.; Ferreiro A.; Fishbein S. R. S.; Dantas G.; Moon T. S. Genetically Stable Crispr-Based Kill Switches for Engineered Microbes. Nat. Commun. 2022, 13, 672 10.1038/s41467-022-28163-5. 35115506 PMC8813983

[ref448] Saeidi N.; Wong C. K.; Lo T. M.; Nguyen H. X.; Ling H.; Leong S. S.; Poh C. L.; Chang M. W. Engineering Microbes to Sense and Eradicate Pseudomonas Aeruginosa, a Human Pathogen. Mol. Syst. Biol. 2011, 7, 521 10.1038/msb.2011.55. 21847113 PMC3202794

[ref449] Reardon S. Crispr Gene-Editing Creates Wave of Exotic Model Organisms. Nature 2019, 568, 441–442. 10.1038/d41586-019-01300-9. 31015699

[ref450] Jiang Y.; Chen B.; Duan C.; Sun B.; Yang J.; Yang S. Multigene Editing in the Escherichia Coli Genome Via the Crispr-Cas9 System. Appl. Environ. Microbiol. 2015, 81, 2506–2514. 10.1128/AEM.04023-14. 25636838 PMC4357945

[ref451] Oh J. H.; van Pijkeren J. P. Crispr-Cas9-Assisted Recombineering in Lactobacillus Reuteri. Nucleic Acids Res. 2014, 42, e131 10.1093/nar/gku623. 25074379 PMC4176153

[ref452] Wang Y.; Zhang Z. T.; Seo S. O.; Lynn P.; Lu T.; Jin Y. S.; Blaschek H. P. Bacterial Genome Editing with Crispr-Cas9: Deletion, Integration, Single Nucleotide Modification, and Desirable ″Clean″ Mutant Selection in Clostridium Beijerinckii as an Example. ACS Synth. Biol. 2016, 5, 721–732. 10.1021/acssynbio.6b00060. 27115041

[ref453] Zheng L.; Tan Y.; Hu Y.; Shen J.; Qu Z.; Chen X.; Ho C. L.; Leung E. L.; Zhao W.; Dai L. Crispr/Cas-Based Genome Editing for Human Gut Commensal Bacteroides Species. ACS Synth. Biol. 2022, 11, 464–472. 10.1021/acssynbio.1c00543. 34990118

[ref454] Chen W.; Zhang Y.; Yeo W. S.; Bae T.; Ji Q. Rapid and Efficient Genome Editing in Staphylococcus Aureus by Using an Engineered Crispr/Cas9 System. J. Am. Chem. Soc. 2017, 139, 3790–3795. 10.1021/jacs.6b13317. 28218837

[ref455] Westbrook A. W.; Moo-Young M.; Chou C. P. Development of a Crispr-Cas9 Tool Kit for Comprehensive Engineering of Bacillus Subtilis. Appl. Environ. Microbiol. 2016, 82, 4876–4895. 10.1128/AEM.01159-16. 27260361 PMC4968543

[ref456] DiCarlo J. E.; Norville J. E.; Mali P.; Rios X.; Aach J.; Church G. M. Genome Engineering in Saccharomyces Cerevisiae Using Crispr-Cas Systems. Nucleic Acids Res. 2013, 41, 4336–4343. 10.1093/nar/gkt135. 23460208 PMC3627607

[ref457] Vyas V. K.; Barrasa M. I.; Fink G. R. A Candida Albicans CRISPR System Permits Genetic Engineering of Essential Genes and Gene Families. Sci. Adv. 2015, 1, e1500248 10.1126/sciadv.1500248. 25977940 PMC4428347

[ref458] Peters J. M.; Colavin A.; Shi H.; Czarny T. L.; Larson M. H.; Wong S.; Hawkins J. S.; Lu C. H. S.; Koo B. M.; Marta E.; et al. A Comprehensive, Crispr-Based Functional Analysis of Essential Genes in Bacteria. Cell 2016, 165, 1493–1506. 10.1016/j.cell.2016.05.003. 27238023 PMC4894308

[ref459] Santos-Moreno J.; Schaerli Y. Crispr-Based Gene Expression Control for Synthetic Gene Circuits. Biochem. Soc. Trans. 2020, 48, 1979–1993. 10.1042/BST20200020. 32964920 PMC7609024

[ref460] Nielsen A. A.; Voigt C. A. Multi-Input Crispr/Cas Genetic Circuits That Interface Host Regulatory Networks. Mol. Syst. Biol. 2014, 10, 763 10.15252/msb.20145735. 25422271 PMC4299604

[ref461] Liu Y.; Zhan Y.; Chen Z.; He A.; Li J.; Wu H.; Liu L.; Zhuang C.; Lin J.; Guo X.; et al. Directing Cellular Information Flow Via Crispr Signal Conductors. Nat. Methods 2016, 13, 938–944. 10.1038/nmeth.3994. 27595406

[ref462] Gao Y.; Xiong X.; Wong S.; Charles E. J.; Lim W. A.; Qi L. S. Complex Transcriptional Modulation with Orthogonal and Inducible Dcas9 Regulators. Nat. Methods 2016, 13, 1043–1049. 10.1038/nmeth.4042. 27776111 PMC5436902

[ref463] Gander M. W.; Vrana J. D.; Voje W. E.; Carothers J. M.; Klavins E. Digital Logic Circuits in Yeast with Crispr-Dcas9 nor Gates. Nat. Commun. 2017, 8, 15459 10.1038/ncomms15459. 28541304 PMC5458518

[ref464] Santos-Moreno J.; Tasiudi E.; Stelling J.; Schaerli Y. Multistable and Dynamic Crispri-Based Synthetic Circuits. Nat. Commun. 2020, 11, 2746 10.1038/s41467-020-16574-1. 32488086 PMC7265303

[ref465] Gaudelli N. M.; Komor A. C.; Rees H. A.; Packer M. S.; Badran A. H.; Bryson D. I.; Liu D. R. Programmable Base Editing of a*T to G*C in Genomic DNA without DNA Cleavage. Nature 2017, 551, 464–471. 10.1038/nature24644. 29160308 PMC5726555

[ref466] Komor A. C.; Kim Y. B.; Packer M. S.; Zuris J. A.; Liu D. R. Programmable Editing of a Target Base in Genomic DNA without Double-Stranded DNA Cleavage. Nature 2016, 533, 420–424. 10.1038/nature17946. 27096365 PMC4873371

[ref467] Peters J. E.; Makarova K. S.; Shmakov S.; Koonin E. V. Recruitment of Crispr-Cas Systems by Tn7-Like Transposons. Proc. Natl. Acad. Sci. U. S. A. 2017, 114, E7358–E7366. 10.1073/pnas.1709035114. 28811374 PMC5584455

[ref468] Faure G.; Shmakov S. A.; Yan W. X.; Cheng D. R.; Scott D. A.; Peters J. E.; Makarova K. S.; Koonin E. V. Crispr-Cas in Mobile Genetic Elements: Counter-Defence and Beyond. Nat. Rev. Microbiol. 2019, 17, 513–525. 10.1038/s41579-019-0204-7. 31165781 PMC11165670

[ref469] Strecker J.; Ladha A.; Gardner Z.; Schmid-Burgk J. L.; Makarova K. S.; Koonin E. V.; Zhang F. Rna-Guided DNA Insertion with Crispr-Associated Transposases. Science 2019, 365, 48–53. 10.1126/science.aax9181. 31171706 PMC6659118

[ref470] Klompe S. E.; Vo P. L. H.; Halpin-Healy T. S.; Sternberg S. H. Transposon-Encoded Crispr-Cas Systems Direct Rna-Guided DNA Integration. Nature 2019, 571, 219–225. 10.1038/s41586-019-1323-z. 31189177

[ref471] Rubin B. E.; Diamond S.; Cress B. F.; Crits-Christoph A.; Lou Y. C.; Borges A. L.; Shivram H.; He C.; Xu M.; Zhou Z.; et al. Species- and Site-Specific Genome Editing in Complex Bacterial Communities. Nat. Microbiol. 2022, 7, 34–47. 10.1038/s41564-021-01014-7. 34873292 PMC9261505

[ref472] Marsh J. W.; Ley R. E. Microbiome Engineering: Taming the Untractable. Cell 2022, 185, 416 10.1016/j.cell.2021.12.034. 35081334

[ref473] Soucy S. M.; Huang J.; Gogarten J. P. Horizontal Gene Transfer: Building the Web of Life. Nat. Rev. Genet. 2015, 16, 472–482. 10.1038/nrg3962. 26184597

[ref474] Neil K.; Allard N.; Rodrigue S. Molecular Mechanisms Influencing Bacterial Conjugation in the Intestinal Microbiota. Front. Microbiol. 2021, 12, 673260 10.3389/fmicb.2021.673260. 34149661 PMC8213034

[ref475] Doucet-Populaire F.; Trieu-Cuot P.; Andremont A.; Courvalin P. Conjugal Transfer of Plasmid DNA from Enterococcus Faecalis to Escherichia Coli in Digestive Tracts of Gnotobiotic Mice. Antimicrob. Agents Chemother. 1992, 36, 502–504. 10.1128/AAC.36.2.502. 1605622 PMC188470

[ref476] Igimi S.; Ryu C. H.; Park S. H.; Sasaki Y.; Sasaki T.; Kumagai S. Transfer of Conjugative Plasmid Pam Beta 1 from Lactococcus Lactis to Mouse Intestinal Bacteria. Lett. Appl. Microbiol. 1996, 23, 31–35. 10.1111/j.1472-765X.1996.tb00023.x. 8679141

[ref477] Ronda C.; Chen S. P.; Cabral V.; Yaung S. J.; Wang H. H. Metagenomic Engineering of the Mammalian Gut Microbiome in Situ. Nat. Methods. 2019, 16, 167–170. 10.1038/s41592-018-0301-y. 30643213 PMC6467691

[ref478] Tridgett M.; Ababi M.; Osgerby A.; Ramirez Garcia R.; Jaramillo A. Engineering Bacteria to Produce Pure Phage-Like Particles for Gene Delivery. ACS Synth. Biol. 2021, 10, 107–114. 10.1021/acssynbio.0c00467. 33317264

[ref479] Voorhees P. J.; Cruz-Teran C.; Edelstein J.; Lai S. K. Challenges & Opportunities for Phage-Based in Situ Microbiome Engineering in the Gut. J. Controlled Release 2020, 326, 106–119. 10.1016/j.jconrel.2020.06.016. 32569705

[ref480] Borodovich T.; Shkoporov A. N.; Ross R. P.; Hill C. Phage-Mediated Horizontal Gene Transfer and Its Implications for the Human Gut Microbiome. Gastroenterol. Rep. 2022, 10, goac012 10.1093/gastro/goac012. PMC9006064 35425613

[ref481] Citorik R. J.; Mimee M.; Lu T. K. Sequence-Specific Antimicrobials Using Efficiently Delivered Rna-Guided Nucleases. Nat. Biotechnol. 2014, 32, 1141–1145. 10.1038/nbt.3011. 25240928 PMC4237163

